# Global, regional, and national burden of stroke, 1990–2016: a systematic analysis for the Global Burden of Disease Study 2016

**DOI:** 10.1016/S1474-4422(19)30034-1

**Published:** 2019-05

**Authors:** GBD 2016 Stroke Collaborators, Catherine Owens Johnson, Minh Nguyen, Gregory A Roth, Emma Nichols, Tahiya Alam, Degu Abate, Foad Abd-Allah, Ahmed Abdelalim, Haftom Niguse Abraha, Niveen ME Abu-Rmeileh, Oladimeji M Adebayo, Abiodun Moshood Adeoye, Gina Agarwal, Sutapa Agrawal, Amani Nidhal Aichour, Ibtihel Aichour, Miloud Taki Eddine Aichour, Fares Alahdab, Raghib Ali, Nelson Alvis-Guzman, Nahla Hamed Anber, Mina Anjomshoa, Jalal Arabloo, Antonio Arauz, Johan Ärnlöv, Amit Arora, Ashish Awasthi, Maciej Banach, Miguel A Barboza, Suzanne Lyn Barker-Collo, Till Winfried Bärnighausen, Sanjay Basu, Abate Bekele Belachew, Yaschilal Muche Belayneh, Derrick A. Bennett, Isabela M Bensenor, Krittika Bhattacharyya, Belete Biadgo, Ali Bijani, Boris Bikbov, Muhammad Shahdaat Bin Sayeed, Zahid A Butt, Lucero Cahuana-Hurtado, Juan J Carrero, Félix Carvalho, Carlos A Castañeda-Orjuela, Franz Castro, Ferrán Catalá-López, Yazan Chaiah, Peggy Pei-Chia Chiang, Jee-Young J Choi, Hanne Christensen, Dinh-Toi Chu, Monica Cortinovis, Albertino Antonio Moura Damasceno, Lalit Dandona, Rakhi Dandona, Ahmad Daryani, Kairat Davletov, Barbora de Courten, Vanessa De la Cruz-Góngora, Meaza Girma Degefa, Samath Dhamminda Dharmaratne, Daniel Diaz, Manisha Dubey, Eyasu Ejeta Duken, Dumessa Edessa, Matthias Endres, EMERITO JOSE A FARAON, Farshad Farzadfar, Eduarda Fernandes, Florian Fischer, Luisa Sorio Flor, Morsaleh Ganji, Abadi Kahsu Gebre, Teklu Gebrehiwo Gebremichael, Birhanu Geta, Kebede Embaye Gezae, Paramjit Singh Gill, Elena V. Gnedovskaya, Hector Gómez-Dantés, Alessandra C Goulart, Giuseppe Grosso, Yuming Guo, Rajeev Gupta, Arvin Haj-Mirzaian, Arya Haj-Mirzaian, Samer Hamidi, Graeme J. Hankey, Hamid Yimam Hassen, Simon I. Hay, Mohamed I Hegazy, Behnam Heidari, Nabeel A Herial, Mohammad Ali Hosseini, Sorin Hostiuc, Seyed Sina Naghibi Irvani, Sheikh Mohammed Shariful Islam, Nader Jahanmehr, Mehdi Javanbakht, Ravi Prakash Jha, Jost B. Jonas, Jacek Jerzy Jozwiak, Mikk Jürisson, Amaha Kahsay, Rizwan Kalani, Yogeshwar Kalkonde, Teshome Abegaz Kamil, Tanuj Kanchan, André Karch, Narges Karimi, Hamidreza Karimi-Sari, Amir Kasaeian, Tesfaye Dessale Kassa, Hossein Kazemeini, Adane Teshome Kefale, Yousef Saleh Khader, Ibrahim A. Khalil, Ejaz Ahmad Khan, Young-Ho Khang, Jagdish Khubchandani, Daniel Kim, Yun Jin Kim, Adnan Kisa, Mika Kivimäki, Ai Koyanagi, Rita K Krishnamurthi, G Anil Kumar, Alessandra Lafranconi, Sarah Lewington, Shanshan Li, Warren David Lo, Alan D Lopez, Stefan Lorkowski, Paulo A. Lotufo, Mark T Mackay, Marek Majdan, Reza Majdzadeh, Azeem Majeed, Reza Malekzadeh, Navid Manafi, Mohammad Ali Mansournia, Man Mohan Mehndiratta, Varshil Mehta, Getnet Mengistu, Atte Meretoja, Tuomo J Meretoja, Bartosz Miazgowski, Tomasz Miazgowski, Ted R Miller, Erkin M Mirrakhimov, Bahram Mohajer, Yousef Mohammad, Milad Mohammadoo-khorasani, Shafiu Mohammed, Farnam Mohebi, Ali H Mokdad, Yaser Mokhayeri, Ghobad Moradi, Lidia Morawska, Ilais Moreno Velásquez, Seyyed Meysam Mousavi, Oumer Sada S Muhammed, Walter Muruet, Mehdi Naderi, Mohsen Naghavi, Gurudatta Naik, Bruno Ramos Nascimento, Ruxandra Irina Negoi, Cuong Tat Nguyen, Long Hoang Nguyen, Yirga Legesse Nirayo, Bo Norrving, Jean Jacques Noubiap, Richard Ofori-Asenso, Felix Akpojene Ogbo, Andrew T Olagunju, Tinuke O Olagunju, Mayowa Ojo Owolabi, Jeyaraj Durai Pandian, Shanti Patel, Norberto Perico, Michael A Piradov, Suzanne Polinder, Maarten J Postma, Hossein Poustchi, V Prakash, Mostafa Qorbani, Alireza Rafiei, Fakher Rahim, Kazem Rahimi, Vafa Rahimi-Movaghar, Mahfuzar Rahman, Muhammad Aziz Rahman, Cesar Reis, Giuseppe Remuzzi, Andre M.N. Renzaho, Stefano Ricci, Nicholas L S Roberts, Stephen R Robinson, Leonardo Roever, Gholamreza Roshandel, Parisa Sabbagh, Hosein Safari, Saeed Safari, Saeid Safiri, Amirhossein Sahebkar, Saleh Salehi Zahabi, Abdallah M. Samy, Paola Santalucia, Itamar S Santos, João Vasco Santos, Milena M Santric Milicevic, Benn Sartorius, Arundhati R Sawant, Aletta Elisabeth Schutte, Sadaf G. Sepanlou, Azadeh Shafieesabet, Masood Ali Shaikh, Mehran Shams-Beyranvand, Aziz Sheikh, Kevin N. Sheth, Kenji Shibuya, Mika Shigematsu, Min-Jeong Shin, Ivy Shiue, Soraya Siabani, Badr Hasan Sobaih, Luciano A. Sposato, Ipsita Sutradhar, PN Sylaja, Cassandra E I Szoeke, Braden James Te Ao, Mohamad-Hani Temsah, Omar Temsah, Amanda G Thrift, Marcello Tonelli, Roman Topor-Madry, Bach Xuan Tran, Khanh Bao Tran, Thomas Clement Truelsen, Afewerki Gebremeskel Tsadik, Irfan Ullah, Olalekan A Uthman, Muthiah Vaduganathan, Pascual R Valdez, Tommi Juhani Vasankari, Rajagopalan Vasanthan, Narayanaswamy Venketasubramanian, Kia Vosoughi, Giang Thu Vu, Yasir Waheed, Elisabete Weiderpass, Kidu Gidey Weldegwergs, Ronny Westerman, Charles D A Wolfe, Dawit Zewdu Wondafrash, Gelin Xu, Ali Yadollahpour, Tomohide Yamada, Hiroshi Yatsuya, Ebrahim M Yimer, Naohiro Yonemoto, Mahmoud Yousefifard, Chuanhua Yu, Zoubida Zaidi, Mohammad Zamani, Afshin Zarghi, Yunquan Zhang, Sanjay Zodpey, Valery L. Feigin, Theo Vos, Christopher J L Murray

## Abstract

**Background:**

Stroke is a leading cause of mortality and disability worldwide and the economic costs of treatment and post-stroke care are substantial. The Global Burden of Diseases, Injuries, and Risk Factors Study (GBD) provides a systematic, comparable method of quantifying health loss by disease, age, sex, year, and location to provide information to health systems and policy makers on more than 300 causes of disease and injury, including stroke. The results presented here are the estimates of burden due to overall stroke and ischaemic and haemorrhagic stroke from GBD 2016.

**Methods:**

We report estimates and corresponding uncertainty intervals (UIs), from 1990 to 2016, for incidence, prevalence, deaths, years of life lost (YLLs), years lived with disability (YLDs), and disability-adjusted life-years (DALYs). DALYs were generated by summing YLLs and YLDs. Cause-specific mortality was estimated using an ensemble modelling process with vital registration and verbal autopsy data as inputs. Non-fatal estimates were generated using Bayesian meta-regression incorporating data from registries, scientific literature, administrative records, and surveys. The Socio-demographic Index (SDI), a summary indicator generated using educational attainment, lagged distributed income, and total fertility rate, was used to group countries into quintiles.

**Findings:**

In 2016, there were 5·5 million (95% UI 5·3 to 5·7) deaths and 116·4 million (111·4 to 121·4) DALYs due to stroke. The global age-standardised mortality rate decreased by 36·2% (−39·3 to −33·6) from 1990 to 2016, with decreases in all SDI quintiles. Over the same period, the global age-standardised DALY rate declined by 34·2% (−37·2 to −31·5), also with decreases in all SDI quintiles. There were 13·7 million (12·7 to 14·7) new stroke cases in 2016. Global age-standardised incidence declined by 8·1% (−10·7 to −5·5) from 1990 to 2016 and decreased in all SDI quintiles except the middle SDI group. There were 80·1 million (74·1 to 86·3) prevalent cases of stroke globally in 2016; 41·1 million (38·0 to 44·3) in women and 39·0 million (36·1 to 42·1) in men.

**Interpretation:**

Although age-standardised mortality rates have decreased sharply from 1990 to 2016, the decrease in age-standardised incidence has been less steep, indicating that the burden of stroke is likely to remain high. Planned updates to future GBD iterations include generating separate estimates for subarachnoid haemorrhage and intracerebral haemorrhage, generating estimates of transient ischaemic attack, and including atrial fibrillation as a risk factor.

**Funding:**

Bill & Melinda Gates Foundation

## Introduction

Globally, stroke is a leading cause of mortality and disability and there are substantial economic costs for post-stroke care.^1^ Results from the 2015 iteration of the Global Burden of Diseases, Injuries, and Risk Factors Study (GBD) showed that although the age-standardised death rates and prevalence of stroke have decreased over time, the overall burden of stroke has remained high.^2^ As populations age, and low-income and middle-income countries go through the epidemiological transition from infectious to non-communicable diseases as the predominant cause of morbidity, together with concomitant increases in modifiable risk factors, it is expected that the burden of stroke will further increase until effective stroke prevention strategies are more widely implemented.^3^

Although estimates of disease burden for stroke have been produced by other research groups by meta-analysing data in the literature on incidence and deaths,^4^, ^5^, ^6^ GBD is unique in its approach to generating estimates for all locations, including those with scarce or no epidemiological data, by using all available data from the literature, administrative hospital and medical claims records, and cause of death records. Additionally, the methods used by GBD allow unspecified stroke to contribute to both fatal and non-fatal estimates. These methods allow GBD to document disease burden from stroke in the most comprehensive way over time and to provide the necessary information for priority setting and planning of health services. The results provided here are the most up-to-date estimates of death, prevalence, incidence, and disability for overall stroke and the pathological types of ischaemic and haemorrhagic stroke, using the standard GBD metrics of deaths, prevalence, incidence, years of life lost (YLLs), years lived with disability (YLDs), and disability-adjusted life-years (DALYs).

**Research in context**

**Evidence before this study**

The Global Burden of Diseases, Injuries, and Risk Factors Study (GBD) produces the only comprehensive estimates of global, regional, and country-specific burden due to stroke from 1990 to 2016. Other sources of population-level estimates include reports from WHO and independent scientific publications of global or regional estimates of deaths or incidence. GBD is the only peer-reviewed, comprehensive assessment of stroke by age, sex, and location that is updated annually. This study quantifies stroke burden in terms of incidence, prevalence, deaths, years lived with disability, years of life lost, and disability-adjusted life-years, updating the estimates previously presented in the GBD 2013 and GBD 2015 studies to include results through 2016. The results presented here are also the source data for the recently published estimates of the lifetime risk of stroke.

**Added value of this study**

There were several important updates to this iteration of GBD, enabling generation of improved estimates. First, we developed new approaches for our inpatient hospital data processing, which allowed us to include data for several locations that had previously been excluded because of insufficient information about the catchment population. Second, we extended the terminal age group of 80 years and older into 80–84 years, 85–89 years, 90–94 years, and 95 years and older. We updated the previous systematic literature review performed as part of GBD 2013 (search terms “stroke” or “hemorrhagic stroke” combined with “incidence”, “prevalence”, “epidemiology”, or “mortality”) to capture any substantial new sources of data on stroke prevalence, incidence, and mortality. We also performed a literature review to inform the models used to generate severity information (search terms “stroke”, “cerebral infarction”, “cerebral hemorrhage”, or “subarachnoid hemorrhage” combined with “Rankin”). Furthermore, we generated expected values for all measures on the basis of sociodemographic development, allowing us to visualise comparisons between observed GBD results and these expected values.

**Implications of all the available evidence**

The findings presented in this manuscript provide crucial information that could serve as the basis for resource allocation for stroke prevention, evidence-based planning for acute stroke care, and stroke rehabilitation facilities. Additionally, we provide evidence that most of the burden of stroke can be attributed to modifiable risk factors and identified risk clusters that can be targeted to reduce the incidence of stroke. Because stroke has been identified as one of the priority areas for WHO and the UN in their actions to reduce the burden of non-communicable diseases, global estimates such as those generated by GBD are essential for appropriately targeting such efforts.

## Methods

### Overview

Methods used to generate estimates of stroke incidence, mortality, prevalence, YLDs, YLLs, and DALYs have been described in previously; additional details are in the appendix.^7^, ^8^ Sources included in all models can be accessed via the GBD 2016 Data Input Sources Tool. For all models, point estimates were calculated from the mean of 1000 draws from the posterior distribution by age, sex, location, and year. 95% uncertainty intervals (UIs) were the 25th and 975th values of the ordered draws. The study was compliant with GATHER guidelines.^9^

### Case definition

Stroke was defined according to WHO criteria as rapidly developing clinical signs of focal (at times global) disturbance of cerebral function lasting more than 24 h or leading to death with no apparent cause other than that of vascular origin.^10^ Data on transient ischaemic attack were not included because of the very short period of disability and no associated mortality for these events. We modelled acute and chronic stroke separately. Stroke cases were considered acute from the day of incidence of a first-ever stroke through day 28 after the event. Stroke cases were considered chronic (prevalent) from 29 days after the occurrence of an event. Chronic stroke included the sequelae of an acute stroke and all recurrent stroke events. 28 days was selected as the cutoff between acute and chronic stroke because this corresponds to the period of early case fatality.^11^

Incident strokes were defined as the occurrence of first-ever stroke on the basis of a clinical diagnosis by a physician according to the WHO criteria described above. Ischaemic strokes were defined as all atherosclerotic and thromboembolic events that resulted in compromised blood flow to brain tissue and subsequent infarction. Haemorrhagic strokes were defined as all non-traumatic events due to subarachnoid or intracerebral haemorrhage identified by neuroimaging.

### Mortality

Standard Cause of Death Ensemble modelling (CODEm) methods were used to estimate cause-specific mortality.^7^ The International Classification of Diseases (ICD) 9 and 10 codes that we used are listed in the appendix. For overall stroke, we included verbal autopsy data in addition to vital registration data; for the stroke type models, we used vital registration data only because accurate assessment of stroke type requires imaging studies that are not commonly available in populations where causes of death are ascertained by verbal autopsy. Covariates included in the models were chosen on the basis of an assessment of causal associations for the risk factors or markers of access to care (appendix).

### Non-fatal disease modelling

We used DisMod-MR 2.1, a Bayesian meta-regression tool, to model the non-fatal burden of stroke.^8^ Estimates were generated using a two-stage modelling approach. In the first stage, we ran four models (acute ischaemic, chronic ischaemic, acute haemorrhagic, and chronic haemorrhagic stroke) using only incidence, prevalence, and excess mortality data as inputs. We then used the ratio of acute to chronic cause-specific mortality estimated by these models to divide the ischaemic-specific and haemorrhagic-specific stroke deaths estimated in CODEm into acute and chronic proportions. The four models were then re-run using the same incidence, prevalence, and excess mortality data as well as the custom cause-specific mortality as input data, thus generating internally consistent fatal and non-fatal estimates.

For the acute models, we used all available high-quality incidence and case fatality data from registries and published literature along with inpatient hospital data on incident events. Acute unspecified stroke was split according to the ratio of ischaemic to haemorrhagic stroke for each combination of sex, age group, and geographic location. The ICD codes used for data from inpatient hospital data sources are listed in the appendix. First-ever, type-specific (ischaemic *vs* haemorrhagic) data from stroke registries were the reference. Datapoints that did not meet our reference case definition, such as those that included recurrent stroke, did not report type-specific data, or only included hospital admissions, were adjusted in DisMod. Prevalence data from surveys, along with the incidence of those surviving the first 28 days calculated from the acute models were included as input data for the chronic models. Counts of data points and covariates and model settings for DisMod are in the appendix. Detailed descriptions of health states, lay descriptions, distributions of functional and cognitive disability, and disability weights for stroke sequelae in GBD 2016 are in the appendix.

### Socio-demographic Index (SDI)

SDI was developed for GBD 2015 as a metric of overall development that positions all locations on a spectrum of socioeconomic development, using educational attainment, lagged distributed income, and total fertility rate. For GBD 2016, this index was updated such that minimum scores are the lowest observed level of GDP per capita or educational attainment or highest observed level of total fertility rate in known datasets. Maximum scores are now plateaus in the relationships between the component parts of the index and selected mortality or health outcomes, indicating no additional benefit to increases in education or lagged distributed income or decreases in total fertility rate. Gaussian process regression was used to establish the average relationship between cause-specific, age-standardised DALY rates and SDI for all locations from 1990 to 2016. These rates were used as the expected values for DALYs in comparisons between observed and expected rates.

### Risk factor estimation

The comparative risk assessment framework developed for GBD was used to estimate levels and trends in attributable burden of stroke due to risk factors that satisfied the criteria of sufficient evidence of a causal relationship, availability of exposure data, and potential for modification.^12^ Four components were incorporated into estimating attributable burden using this approach: (1) burden estimates for stroke; (2) exposure levels for each risk factor; (3) relative risk of stroke as an outcome of exposure to the risk factor; and (4) theoretical minimum risk exposure level—ie, the level of exposure that minimises risk for each individual in the population. The population attributable fraction, estimated independently for each risk factor, is the proportion of the cause that would be decreased if the exposure to the risk factor in the past had been reduced to the counterfactual level of the theoretical minimum risk exposure level. Estimates of attributable burden for each risk–outcome pair were established by multiplying the relevant cause measure by the population attributable fraction. All estimates of attributable burden are generated at the most detailed level and estimates for risk groupings or all risk factors combined are generated via an aggregation process that accounts for the fact that the effect of one risk factor might be partly or completely mediated through the effect of another. This mediation analysis is informed by individual-level data from prospective cohort studies on the joint effects of combinations of risk factors.

### Role of the funding source

The funder of the study had no role in study design, data collection, data analysis, data interpretation, or the writing of the report. All authors had full access to the data in the study and had final responsibility for the decision to submit for publication.

## Results

GBD stroke estimates for 1990–2016 are available for download from the GBD Results Tool at the Global Health Data Exchange. In 2016, stroke was the second largest cause of death globally (5·5 million [95% UI 5·3–5·7] deaths) after ischaemic heart disease (table). Fewer women died as a result of stroke (2·6 million [2·5–2·7] deaths) than did men (2·9 million [2·8–3·0] deaths). The number of global deaths due to ischaemic stroke (2·7 million [2·6–2·8]) was slightly lower than the number due to haemorrhagic stroke (2·8 million [2·7–2·9] deaths; appendix). Stroke was also the second most common cause of global DALYs (116·4 million [111·4 −121·4]), an increase from 1990 (95·3 million [91·6–100·6]). Women had fewer stroke DALYs (50·8 million [47·6–53·7]) than men (65·6 million [63·1–68·2]). The number of DALYs due to ischaemic stroke (51·9 million [47·9–55·6]) was lower than the number due to haemorrhagic stroke (64·5 million [62·6–66·5]; appendix). There were 80·1 million (74·1–86·3) prevalent cases of stroke globally in 2016: 41·1 million (38·0–44·3) prevalent cases in women and 39·0 million (36·1–42·1) prevalent cases in men. Of the total number of prevalent strokes, 84·4% (82·1–86·4) were ischaemic. There were 13·7 million (12·7–14·7) new stroke cases in 2016.

**Table:** Deaths, Incident cases, and DALYs for stroke in 2016 and percentage change of age-standardised rates for 1990–2016, by location

	**Deaths (95% uncertainty interval)**		**Incident cases (95% uncertainty interval)**		**DALYs (95% uncertainty interval)**	
	2016 counts	Percentage change in age-standardised rates, 1990–2016	2016 counts	Percentage change in age-standardised rates, 1990–2016	2016 counts	Percentage change in age-standardised rates, 1990–2016
**Global**	**5 528 232 (5 334 609 to 5 734 681)**	**−36·2% (−39·3 to −33·6)**	**13 676 761 (12 713 488 to 14 692 386)**	**−8·1% (−10·7 to −5·5)**	**116 445 136 (111 385 357 to 121 406 862)**	**−34·2% (−37·2 to −31·5)**
High SDI	721 049 (674 368 to 770 105)	−51·9% (−53·5 to −50·4)	2 496 143 (2 325 267 to 2 672 119)	−20·3% (−22·8 to −17·8)	11 428 239 (10 474 984 to 12 313 359)	−49·3% (−51·4 to −47·5)
High-middle SDI	1 082 392 (989 070 to 1 191 869)	−44·7% (−49·3 to −39·2)	3 218 009 (2 966 203 to 3 470 057)	−15·9% (−19·1 to −12·5)	20 886 507 (19 041 515 to 22 862 878)	−42·3% (−46·7 to −37·1)
Middle SDI	2 229 002 (2 156 876 to 2 302 482)	−38·2% (−43·5 to −34·3)	5 394 853 (5 006 115 to 5 782 067)	0·3% (−2·6 to 3·0)	48 552 584 (46 534 278 to 50 601 668)	−37·3% (−42·1 to −33·6)
Low-middle SDI	1 181 709 (1 124 199 to 1 234 945)	−22·7% (−28·4 to −16·9)	2 062 294 (1 900 903 to 2 221 193)	−2·8% (−5·1 to −0·4)	27 582 829 (26 339 529 to 28 769 222)	−24·6% (−29·5 to −19·4)
Low SDI	311 001 (290 881 to 331 322)	−20·8% (−25·9 to −14·0)	445 405 (408 264 to 481 496)	−8·0% (−10·6 to −5·4)	7 886 374 (7 409 345 to 8 335 178)	−23·2% (−27·8 to −17·0)
**High-income North America**	**195 661 (185 354 to 206 778)**	**−21·1% (−24·0 to −18·3)**	**812 285 (756 263 to 873 750)**	**−14·0% (−16·5 to −11·4)**	**3 451 975 (3 161 432 to 3 716 553)**	**−21·8% (−24·6 to −19·3)**
Canada	18 433 (16 889 to 20 236)	−38·3% (−43·3 to −32·8)	80 683 (73 776 to 88 270)	−17·2% (−21·7 to −12·6)	288 427 (254 406 to 318 530)	−37·2% (−41·5 to −32·9)
Greenland	33 (26 to 42)	−55·3% (−62·7 to −46·7)	87 (78 to 96)	−33·4% (−36·8 to −29·5)	758 (614 to 933)	−53·2% (−61·1 to −43·7)
USA	177 196 (167 723 to 187 486)	−19·1% (−22·2 to −16·0)	731 256 (680 564 to 785 696)	−13·6% (−16·2 to −10·9)	3 162 485 (2 900 887 to 3 404 445)	−20·1% (−22·9 to −17·4)
**Australasia**	**16 070 (14 600 to 17 490)**	**−48·7% (−52·7 to −44·6)**	**46 733 (43 598 to 50 167)**	**−23·5% (−26·8 to −20·0)**	**206 799 (187 319 to 225 047)**	**−51·1% (−54·4 to −47·9)**
Australia	13 480 (12 092 to 14 832)	−49·3% (−53·9 to −44·5)	37 091 (34 266 to 40 137)	−27·5% (−31·0 to −23·5)	170 962 (153 916 to 186 120)	−51·2% (−55·0 to −47·6)
New Zealand	2589 (2292 to 2924)	−46·1% (−52·0 to −40·0)	9642 (8923 to 10 183)	−3·0% (−9·8 to 2·9)	35 837 (32 033 to 39 969)	−50·2% (−54·6 to −45·2)
**High-income Asia Pacific**	**160 610 (146 873 to 174 157)**	**−66·3% (−68·8 to −63·4)**	**448 853 (414 801 to 483 777)**	**−33·1% (−35·5 to −30·4)**	**2 489 972 (2 245 941 to 2 724 866)**	**−61·5% (−64·9 to −58·1)**
Brunei	131 (110 to 150)	−40·9% (−49·1 to −32·1)	393 (356 to 427)	−26·8% (−30·4 to −23·4)	3443 (2882 to 4016)	−42·9% (−51·1 to −34·1)
Japan	122 032 (112 574 to 131 181)	−64·2% (−65·8 to −62·5)	353 551 (326 496 to 381 049)	−28·6% (−31·2 to −25·8)	1 797 708 (1 636 262 to 1 947 883)	−55·9% (−58·0 to −53·8)
Singapore	1162 (951 to 1400)	−74·3% (−79·1 to −68·3)	5915 (5399 to 6473)	−37·8% (−41·1 to −34·2)	27 116 (22 845 to 31 866)	−68·2% (−73·3 to −62·4)
South Korea	37 285 (28 773 to 46 655)	−73·8% (−80·1 to −65·8)	88 993 (81 334 to 97 072)	−49·6% (−52·9 to −46·1)	661 705 (527 331 to 810 431)	−74·5% (−80·0 to −67·7)
**Western Europe**	**310 011 (284 276 to 339 482)**	**−58·4% (−60·4 to −56·2)**	**1 036 438 (964 975 to 1 108 323)**	**−22·7% (−25·6 to −19·8)**	**4 350 012 (3 952 234 to 4 707 683)**	**−56·4% (−58·6 to −54·4)**
Andorra	48 (39 to 59)	−41·8% (−54·8 to −25·7)	188 (172 to 206)	−15·7% (−19·4 to −12·0)	700 (580 to 829)	−35·9% (−47·5 to −22·0)
Austria	3888 (3361 to 4500)	−74·9% (−77·2 to −72·2)	23 698 (21 898 to 25 595)	−28·3% (−32·4 to −23·8)	68 833 (59 863 to 77 120)	−67·1% (−70·2 to −64·0)
Belgium	7825 (6806 to 8886)	−55·1% (−60·2 to −50·0)	28 085 (25 721 to 30 517)	−17·3% (−22·4 to −11·1)	116 340 (103 430 to 130 062)	−51·5% (−56·3 to −46·8)
Cyprus	500 (439 to 562)	−60·0% (−65·8 to −53·2)	1573 (1459 to 1701)	−27·7% (−31·1 to −24·1)	7522 (6731 to 8290)	−56·8% (−62·5 to −51·1)
Denmark	4013 (3538 to 4533)	−46·4% (−53·0 to −39·1)	12 540 (11 542 to 13 642)	−24·3% (−28·2 to −20·1)	60 016 (53 417 to 67 325)	−49·2% (−54·9 to −43·3)
Finland	5130 (4430 to 5912)	−53·5% (−58·6 to −47·7)	17 429 (15 988 to 18 989)	−18·0% (−22·9 to −12·7)	75 047 (66 486 to 84 678)	−54·5% (−58·7 to −50·1)
France	38 557 (34 514 to 43 256)	−55·9% (−59·6 to −52·3)	131 416 (121 049 to 142 111)	−21·2% (−25·6 to −16·7)	548 745 (491 599 to 601 426)	−50·7% (−54·3 to −47·1)
Germany	57 717 (50 943 to 65 847)	−62·3% (−66·3 to −57·4)	242 497 (221 808 to 265 229)	−14·4% (−20·4 to −7·6)	926 146 (817 905 to 1 028 186)	−57·8% (−61·6 to −53·6)
Greece	15 891 (14 179 to 17 757)	−54·7% (−58·9 to −50·3)	34 149 (31 480 to 36 859)	−27·5% (−31·5 to −23·2)	200 543 (181 556 to 220 325)	−51·6% (−55·4 to −47·7)
Iceland	163 (143 to 184)	−42·8% (−48·9 to −36·5)	603 (556 to 656)	−16·0% (−20·5 to −11·4)	2342 (2051 to 2610)	−46·2% (−51·1 to −41·2)
Ireland	1915 (1654 to 2181)	−60·8% (−65·8 to −55·0)	7462 (6854 to 8100)	−30·4% (−34·3 to −26·5)	31 653 (27 546 to 35 902)	−58·4% (−63·2 to −53·5)
Israel	2740 (2318 to 3226)	−63·4% (−69·8 to −56·1)	11 390 (10 473 to 12 351)	−31·0% (−34·7 to −27·0)	45 122 (38 699 to 52 316)	−60·9% (−67·1 to −54·4)
Italy	52 327 (45 538 to 60 768)	−57·9% (−62·2 to −53·1)	166 015 (158 060 to 172 946)	−22·2% (−25·8 to −18·3)	641 405 (574 753 to 712 130)	−58·7% (−62·3 to −55·1)
Luxembourg	334 (294 to 377)	−69·0% (−72·5 to −65·2)	1074 (1007 to 1142)	−37·7% (−40·9 to −34·2)	4966 (4407 to 5514)	−66·7% (−70·0 to −63·1)
Malta	250 (212 to 293)	−60·5% (−66·7 to −53·1)	892 (819 to 968)	−30·7% (−34·4 to −26·7)	4105 (3517 to 4722)	−59·2% (−64·7 to −52·9)
Netherlands	11 132 (9950 to 12 434)	−41·4% (−47·0 to −34·6)	35 385 (32 575 to 38 469)	−16·4% (−21·1 to −11·4)	162 107 (145 787 to 178 649)	−42·9% (−47·7 to −37·8)
Norway	2947 (2560 to 3353)	−58·2% (−63·4 to −52·7)	12 254 (11 292 to 13 321)	−18·6% (−23·3 to −13·7)	43 207 (37 977 to 48 478)	−54·6% (−59·2 to −50·0)
Portugal	14 112 (12 858 to 15 478)	−69·6% (−71·9 to −67·1)	27 447 (25 466 to 29 628)	−51·0% (−53·5 to −48·3)	187 018 (171 638 to 202 338)	−68·8% (−71·2 to −66·4)
Spain	29 646 (26 209 to 33 330)	−64·3% (−67·7 to −61·1)	101 845 (93 604 to 110 539)	−31·0% (−34·8 to −26·9)	389 291 (348 750 to 425 747)	−62·3% (−65·3 to −59·5)
Sweden	7810 (6755 to 8965)	−39·8% (−47·0 to −32·2)	24 807 (22 713 to 27 014)	−11·5% (−16·0 to −6·6)	103 126 (90 822 to 115 994)	−42·3% (−47·9 to −36·3)
Switzerland	4439 (3482 to 5643)	−61·9% (−69·7 to −52·8)	19 766 (18 156 to 21 319)	−13·0% (−18·3 to −6·0)	63 410 (51 656 to 75 635)	−56·9% (−63·6 to −49·4)
UK	48 628 (45 348 to 51 909)	−52·6% (−54·3 to −50·9)	134 979 (125 162 to 145 532)	−26·9% (−29·4 to −24·2)	667 392 (615 643 to 717 146)	−52·7% (−54·7 to −50·9)
**Southern Latin America**	**35 357 (32 341 to 38 404)**	**−53·2% (−57·0 to −48·8)**	**95 250 (87 970 to 102 544)**	**−33·3% (−36·4 to −29·7)**	**666 622 (607 737 to 724 526)**	**−54·1% (−57·7 to −50·0)**
Argentina	22 010 (20 003 to 23 935)	−54·5% (−58·5 to −50·1)	59 608 (55 163 to 64 328)	−35·4% (−39·1 to −31·3)	434 748 (395 748 to 470 803)	−55·2% (−59·0 to −51·0)
Chile	9869 (7957 to 12 096)	−51·9% (−61·5 to −40·6)	28 412 (26 063 to 30 948)	−28·9% (−33·1 to −24·2)	179 122 (146 291 to 216 151)	−53·1% (−61·9 to −43·5)
Uruguay	3478 (3210 to 3758)	−45·3% (−49·4 to −40·6)	7223 (6650 to 7831)	−29·5% (−33·3 to −25·6)	52 744 (48 724 to 56 787)	−46·3% (−50·0 to −41·9)
Eastern Europe	461 418 (377 592 to 561 768)	−29·0% (−41·4 to −13·2)	962 562 (866 533 to 1 055 913)	−15·3% (−20·3 to −9·8)	8 235 892 (6 888 360 to 9 951 417)	−24·8% (−37·5 to −9·5)
Belarus	14 437 (12 228 to 16 700)	−26·8% (−38·0 to −15·3)	37 939 (34 401 to 41 493)	−13·8% (−19·0 to −7·9)	281 651 (241 304 to 321 826)	−26·9% (−37·4 to −16·4)
Estonia	1200 (978 to 1471)	−74·3% (−78·9 to −68·1)	4610 (4158 to 5073)	−37·3% (−41·6 to −32·5)	23 179 (19 458 to 27 383)	−68·5% (−73·0 to −63·0)
Latvia	4512 (3962 to 5102)	−46·8% (−53·0 to −39·3)	12 188 (10 842 to 13 537)	−16·0% (−22·7 to −7·6)	73 098 (64 510 to 82 141)	−43·7% (−49·8 to −36·6)
Lithuania	4435 (4018 to 4859)	−20·3% (−27·6 to −12·4)	15 035 (13 648 to 16 300)	−1·3% (−7·3 to 5·0)	77 217 (69 927 to 84 557)	−24·1% (−30·3 to −17·3)
Moldova	55904995 to 6235)	−34·0% (−41·8 to −25·2)	12 925 (11 830 to 13 966)	−17·2% (−21·6 to −12·5)	119 356 (106 888 to 132 707)	−29·6% (−37·8 to −20·4)
Russia	345 861 (267 315 to 444 861)	−26·5% (−43·5 to −4·8)	676 846 (607 894 to 746 828)	−14·6% (−20·4 to −8·3)	6 082 727 (4 773 920 to 7 736 480)	−22·4% (−39·4 to −0·5)
Ukraine	85 383 (69 613 to 105 349)	−37·8% (−49·4 to −23·4)	203 018 (183 022 to 223 100)	−19·0% (−24·5 to −13·0)	1 578 664 (1 313 971 to 1 902 057)	−31·4% (−43·1 to −16·7)
**Central Europe**	**177 467 (166 446 to 191 258)**	**−43·8% (−46·6 to −40·7)**	**467 197 (432 780 to 499 536)**	**−14·9% (−18·6 to −11·1)**	**2 970 660 (2 770 447 to 3 170 163)**	**−44·4% (−47·0 to −41·6)**
Albania	4751 (4108 to 5374)	−8·7% (−20·7 to 3·1)	8436 (7768 to 9130)	0·5% (−4·2 to 5·5)	73 918 (63 986 to 82 726)	−12·9% (−23·3 to −3·1)
Bosnia and Herzegovina	6446 (5608 to 7434)	−37·9% (−47·8 to −26·4)	16 687 (15 103 to 18 272)	−1·8% (−7·5 to 3·7)	112 114 (98 480 to 127 965)	−37·2% (−46·4 to −26·6)
Bulgaria	20 458 (17 924 to 23 249)	−34·4% (−43·3 to −24·6)	38 368 (34 899 to 41 894)	−14·8% (−19·9 to −9·7)	327 622 (287 167 to 369 674)	−36·5% (−44·7 to −27·8)
Croatia	7585 (6608 to 8547)	−43·2% (−51·1 to −35·0)	20 469 (19 234 to 21 532)	−10·4% (−15·8 to −4·3)	118 848 (105 388 to 132 189)	−46·5% (−53·1 to −39·4)
Czech Republic	10 169 (9355 to 11 037)	−70·6% (−73·0 to −67·8)	38 959 (35 267 to 42 806)	−30·0% (−34·7 to −24·3)	165 197 (149 489 to 181 357)	−68·5% (−71·1 to −65·6)
Hungary	13 188 (11 698 to 14 703)	−55·8% (−60·9 to −50·3)	40 003 (36 296 to 43 822)	−26·0% (−30·7 to −20·8)	232 778 (207 864 to 257 571)	−55·0% (−59·6 to −50·1)
Macedonia	4596 (4085 to 5567)	−22·2% (−29·4 to −14·9)	8147 (7377 to 8881)	−15·0% (−19·9 to −10·2)	79 720 (72 394 to 91 380)	−26·0% (−32·2 to −19·6)
Montenegro	1500 (1319 to 1662)	−12·1% (−24·0 to 1·4)	2346 (2162 to 2556)	−6·1% (−10·3 to −1·8)	23 140 (20 482 to 25 575)	−19·5% (−29·4 to −8·5)
Poland	35 815 (31 974 to 40 055)	−49·1% (−54·4 to −43·4)	124 540 (113 864 to 132 877)	−7·4% (−13·8 to 0·2)	653 330 (585 572 to 720 870)	−47·9% (−52·5 to −42·4)
Romania	49 042 (44 527 to 54 190)	−27·0% (−33·8 to −19·6)	103 102 (93 806 to 112 993)	−11·3% (−17·4 to −3·8)	776 798 (704 821 to 853 437)	−30·6% (−36·7 to −24·1)
Serbia	17 092 (14 972 to 20 989)	−34·5% (−42·1 to −26·2)	39 375 (37 480 to 41 089)	−14·6% (−20·1 to −8·7)	284 448 (254 852 to 329 446)	−36·7% (−43·1 to −29·5)
Slovakia	5056 (4456 to 5673)	−49·5% (−56·2 to −42·4)	20 560 (18 662 to 22 686)	−4·0% (−10·5 to 3·7)	95 249 (83 904 to 106 547)	−48·3% (−54·6 to −41·6)
Slovenia	1767 (1497 to 2079)	−68·1% (−72·7 to −63·2)	6204 (5829 to 6591)	−34·3% (−38·0 to −30·5)	27 499 (23 813 to 31 113)	−66·5% (−71·0 to −62·0)
**Central Asia**	**73 150 (68 710 to 78 547)**	**−25·6% (−29·9 to −20·7)**	**141 713 (131 302 to 151 650)**	**−14·1% (−17·0 to −10·9)**	**1 606 521 (1 500 356 to 1 725 247)**	**−24·0% (−28·1 to −19·3)**
Armenia	2355 (2097 to 2615)	−48·6% (−54·5 to −42·4)	6639 (6073 to 7203)	−20·5% (−24·9 to −16·1)	45 047 (40 282 to 49 428)	−44·8% (−50·3 to −38·9)
Azerbaijan	8022 (6726 to 9484)	−22·4% (−34·8 to −8·4)	17 221 (15 765 to 18 656)	−3·0% (−7·3 to 2·1)	171 457 (145 438 to 200 888)	−23·2% (−35·8 to −9·6)
Georgia	8978 (7770 to 10 276)	−27·4% (−38·5 to −15·4)	14 229 (13 415 to 15 012)	−10·9% (−15·4 to −6·0)	146 412 (126 819 to 168 149)	−29·7% (−40·1 to −18·2)
Kazakhstan	17 699 (15 216 to 20 895)	−25·6% (−36·4 to −11·6)	35 801 (32 918 to 38 755)	−15·2% (−19·7 to −9·8)	389 587 (335 807 to 458 020)	−24·2% (−34·7 to −10·1)
Kyrgyzstan	4588 (4180 to 5023)	−35·6% (−41·6 to −29·3)	8113 (7483 to 8759)	−24·6% (−28·4 to −20·4)	107 238 (97 607 to 117 291)	−30·4% (−36·6 to −23·7)
Mongolia	3338 (2918 to 3785)	55·7% (32·8 to 84·7)	4495 (4161 to 4837)	22·3% (17·5 to 27·1)	89 526 (77 373 to 102 783)	44·3% (22·5 to 70·7)
Tajikistan	4801 (4214 to 5562)	−10·3% (−21·7 to 4·4)	8791 (8109 to 9465)	−10·7% (−15·0 to −6·3)	102 986 (89 879 to 120 358)	−15·0% (−26·2 to −0·9)
Turkmenistan	4145 (3843 to 4436)	−12·3% (−19·0 to −4·9)	6850 (6306 to 7389)	−0·2% (−4·3 to 4·3)	108 988 (101 370 to 116 400)	−11·3% (−17·8 to −4·2)
Uzbekistan	19 223 (16 854 to 22 305)	−25·5% (−34·7 to −16·4)	39 574 (36 333 to 42 740)	−15·0% (−19·2 to −11·0)	445 281 (390 470 to 516 727)	−24·2% (−33·6 to −15·2)
**Central Latin America**	**60 687 (56 477 to 64 599)**	**−42·6% (−45·9 to −39·4)**	**210 120 (191 977 to 227 671)**	**−13·5% (−16·6 to −10·2)**	**1 370 692 (1 292 563 to 1 445 939)**	**−43·2% (−46·1 to −40·2)**
Colombia	11 830 (10 388 to 13 213)	−54·4% (−60·0 to −48·6)	42 277 (38 454 to 46 181)	−25·4% (−29·0 to −21·1)	249 664 (221 520 to 275 392)	−58·1% (−63·2 to −53·2)
Costa Rica	989 (878 to 1120)	−52·2% (−56·9 to −47·0)	4696 (4273 to 5151)	−13·7% (−18·1 to −8·6)	19 996 (18 023 to 22 104)	−47·2% (−51·9 to −42·1)
El Salvador	1330 (1167 to 1510)	−67·1% (−71·3 to −62·6)	5109 (4639 to 5561)	−25·3% (−29·2 to −20·6)	29 566 (26 459 to 33 305)	−68·8% (−72·4 to −64·6)
Guatemala	3397 (2694 to 4177)	−20·5% (−37·1 to −1·0)	10 008 (9154 to 10 863)	−2·7% (−7·7 to 2·8)	87 714 (69 883 to 107 167)	−26·6% (−41·8 to −8·1)
Honduras	2698 (2167 to 3373)	−39·3% (−52·3 to −21·9)	6283 (5790 to 6813)	−12·7% (−17·0 to −8·2)	81 325 (66 609 to 99 797)	−47·5% (−57·9 to −32·4)
Mexico	27 738 (25 840 to 29 530)	−36·4% (−39·5 to −33·6)	104 877 (95 902 to 113 520)	−6·5% (−9·5 to −3·1)	626 689 (592 408 to 657 379)	−34·3% (−37·2 to −31·5)
Nicaragua	1333 (1134 to 1564)	−37·8% (−47·2 to −26·9)	4641 (4242 to 5066)	−11·4%–15·5 to −6·8)	28 456 (24 433 to 32 957)	−40·0% (−48·8 to −29·9)
Panama	1448 (1271 to 1635)	−47·1% (−54·3 to −39·2)	3996 (3678 to 4316)	−24·5% (−28·2 to −20·8)	26 905 (23 720 to 30 390)	−46·8% (−54·0 to −38·8)
Venezuela	9922 (8452 to 11 653)	−39·4% (−48·7 to −28·2)	28 233 (25 871 to 30 579)	−18·1% (−22·3 to −13·4)	220 376 (188 959 to 259 518)	−39·5% (−48·6 to −28·4)
**Andean Latin America**	**14 122 (12 682 to 15 704)**	**−54·9% (−59·9 to −49·3)**	**49 970 (45 817 to 54 162)**	**−20·5% (−23·9 to −16·7)**	**330 016 (298 765 to 369 404)**	**−57·1% (−61·9 to −51·6)**
Bolivia	4214 (3423 to 5168)	−49·6% (−59·3 to −37·2)	10 349 (9482 to 11 278)	−18·2% (−22·0 to −13·8)	96 482 (78 381 to 119 222)	−53·5% (−62·7 to −41·8)
Ecuador	4035 (3681 to 4440)	−49·3% (−54·0 to −43·9)	13 309 (12 170 to 14 431)	−21·6% (−25·8 to −17·2)	93 289 (84 922 to 101 568)	−52·0% (−56·5 to −47·2)
Peru	5873 (4904 to 7017)	−60·8% (−68·2 to −51·4)	26 312 (24 013 to 28 662)	−21·0% (−25·1 to −16·3)	140 244 (117 392 to 167 236)	−61·6% (−68·5 to −52·6)
**Caribbean**	**33 297 (30 836 to 35 833)**	**−28·3% (−33·3 to −23·0)**	**63 459 (58 850 to 68 445)**	**−15·6% (−18·5 to −12·4)**	**659 354 (599 695 to 720 059)**	**−34·9% (−40·6 to −29·2)**
Antigua and Barbuda	46 (40 to 52)	−51·6% (−58·0 to −44·4)	110 (101 to 119)	−27·0% (−30·7 to −23·0)	930 (819 to 1043)	−50·3% (−56·6 to −43·2)
The Bahamas	246 (210 to 277)	−26·9% (−35·8 to −17·9)	515 (475 to 558)	−15·6% (−19·8 to −11·6)	5048 (4404 to 5630)	−30·8% (−38·3 to −22·9)
Barbados	272 (245 to 299)	−42·8% (−49·0 to −36·4)	553 (509 to 598)	−22·9% (−27·0 to −18·7)	4429 (4020 to 4873)	−40·6% (−46·4 to −33·7)
Belize	118 (102 to 134)	−6·1% (−19·2 to 7·9)	255 (234 to 276)	−7·1% (−11·3 to −3·0)	2662 (2330 to 3017)	−15·7% (−27·3 to −3·3)
Bermuda	31 (26 to 35)	−60·6% (−66·2 to −54·1)	81 (74 to 88)	−35·2% (−38·5 to −31·4)	516 (453 to 587)	−62·9% (−68·1 to −57·2)
Cuba	9684 (8801 to 10 585)	−23·0% (−30·5 to −15·0)	21 416 (19 729 to 23 177)	−13·3% (−17·4 to −8·9)	161 026 (147 535 to 175 492)	−30·6% (−37·1 to −24·0)
Dominica	50 (43 to 58)	−28·3% (−38·9 to −16·1)	97 (90 to 105)	−13·1% (−17·3 to −8·1)	883 (769 to 1007)	−26·8% (−36·6 to −14·6)
Dominican Republic	5395 (4588 to 6191)	−30·7% (−41·5 to −20·0)	11 365 (10 440 to 12 334)	−16·1% (−20·5 to −11·2)	101 780 (86 334 to 116 349)	−37·4% (−47·0 to −27·7)
Grenada	92 (81 to 104)	−31·3% (−41·1 to −21·4)	141 (131 to 152)	−18·8% (−22·8 to −14·5)	1686 (1474 to 1914)	−32·5% (−42·7 to −22·0)
Guyana	688 (608 to 766)	−38·9% (−46·6 to −31·3)	953 (877 to 1032)	−26·7% (−30·1 to −23·2)	16 562 (14 631 to 18 448)	−45·2% (−52·0 to −38·5)
Haiti	9944 (7864 to 12 110)	−31·4% (−43·4 to −18·1)	10 982 (10 111 to 11 846)	−23·0% (−26·5 to −19·2)	246 846 (193 785 to 303 079)	−38·3% (−49·6 to −24·9)
Jamaica	3021 (2599 to 3462)	−17·4% (−30·6 to −1·6)	4568 (4222 to 4945)	−14·1% (−18·6 to −9·9)	48 039 (41 181 to 55 041)	−26·7% (−38·3 to −13·3)
Puerto Rico	1917 (1711 to 2135)	−18·0% (−26·9 to −8·3)	5869 (5392 to 6391)	0·1% (−4·7 to 5·2)	30 944 (27 904 to 34 054)	−22·1% (−29·7 to −13·8)
Saint Lucia	124 (111 to 134)	−48·6% (−53·5 to −43·9)	252 (233 to 271)	−26·3% (−29·7 to −22·6)	2331 (2130 to 2516)	−47·9% (−52·7 to −43·3)
Saint Vincent and the Grenadines	91 (81 to 100)	−16·2% (−25·4 to −6·3)	150 (138 to 161)	−10·1% (−14·0 to −5·3)	1774 (1601 to 1934)	−18·5% (−27·0 to −9·2)
Suriname	482437 to 526)	−0·2%–9·3 to 9·8)	775 (717 to 833)	−7·9% (−12·1 to −3·6)	10 079 (9183 to 10 953)	−9·4% (−17·4 to −0·4)
Trinidad and Tobago	1002916 to 1097)	−43·7%–48·9 to −38·1)	2117 (1949 to 2284)	−26·7% (−30·4 to −23·2)	19 968 (18 222 to 21 809)	−45·4% (−50·0 to −40·0)
Virgin Islands	95 (83 to 107)	−25·2% (−35·4 to −13·2)	224 (206 to 245)	−8·2% (−12·3 to −3·6)	1675 (1464 to 1886)	−29·8% (−39·2 to −19·0)
**Tropical Latin America**	**111 098 (105 139 to 116 401)**	**−55·5% (−57·3 to −53·8)**	**264 861 (243 959 to 285 940)**	**−30·8% (−33·2 to −28·3)**	**2 306 945 (2 188 842 to 2 413 612)**	**−56·5% (−58·2 to −54·9)**
Brazil	107 656 (101 751 to 113 029)	−56·2% (−58·0 to −54·4)	258 021 (237 631 to 278 470)	−31·2% (−33·7 to −28·7)	2 236 740 (2 122 674 to 2 341 269)	−57·2% (−58·8 to −55·5)
Paraguay	3442 (3005 to 3897)	−21·9% (−32·0 to −10·5)	6840 (6301 to 7366)	−11·1% (−15·0 to −6·8)	70 204 (62 424 to 78 686)	−22·6% (−31·9 to −11·2)
**East Asia**	**1 848 933 (1 782 311 to 1 917 491)**	**−42·3% (−48·5 to −38·3)**	**5 619 517 (5 226 581 to 6 008 490)**	**4·9% (1·3 to 8·1)**	**39 931 397 (37 820 465 to 41 958 598)**	**−41·0% (−46·5 to −36·9)**
China	1 790 033 (1 725 729 to 1 857 796)	−42·6% (−48·9 to −38·3)	5 510 276 (5 123 307 to 5 891 047)	5·4% (1·7 to 8·7)	38 623 565 (36 559 198 to 40 646 436)	−41·5% (−47·0 to −37·3)
North Korea	46 224 (40 908 to 51 736)	25·7% (8·2 to 46·2)	64 094 (59 359 to 68 754)	10·3% (5·9 to 15·1)	1 021 113 (909 221 to 1 130 202)	26·7% (10·3 to 46·1)
Taiwan (province of China)	12 675 (10 878 to 14 526)	−69·9% (−74·2 to −65·4)	45 147 (41 139 to 49 211)	−31·7% (−35·1 to −27·8)	286 720 (246 488 to 326 346)	−63·3% (−67·7 to −58·8)
**Southeast Asia**	**504 522 (481 493 to 529 723)**	**−22·0% (−27·5 to −15·0)**	**811 510 (747 194 to 871 724)**	**−7·1% (−9·4 to −4·5)**	**11 693 267 (11 134 566 to 12 283 903)**	**−20·1% (−25·1 to −14·2)**
Cambodia	11 791 (10 749 to 12 889)	−24·4% (−35·8 to −5·8)	14 604 (13 484 to 15 730)	−13·0% (−16·2 to −9·0)	275 137 (249 972 to 301 718)	−28·4% (−38·8 to −12·8)
Indonesia	212 963 (200 341 to 227 120)	6·3% (−4·3 to 20·3)	334 295 (306 106 to 360 209)	8·8% (6·2 to 11·6)	5 175 449 (4 890 704 to 5 488 841)	6·0% (−2·4 to 16·8)
Laos	4834 (4194 to 5378)	−27·6% (−36·8 to −14·1)	6242 (5756 to 6732)	−13·1% (−16·6 to −9·6)	125 305 (109 568 to 138 953)	−32·2% (−40·8 to −21·3)
Malaysia	14 302 (13 076 to 15 506)	−46·0% (−51·4 to −39·9)	33 628 (30 741 to 36 542)	−16·8% (−20·2 to −13·1)	337 935 (307 813 to 367 078)	−46·5% (−51·5 to −41·2)
Maldives	83 (67 to 102)	−65·3% (−72·9 to −55·7)	283 (256 to 310)	−25·1% (−28·9 to −21·0)	1940 (1601 to 2343)	−66·2% (−74·2 to −57·3)
Mauritius	820 (715 to 930)	−63·6% (−68·2 to −58·7)	1838 (1674 to 1991)	−35·6% (−38·8 to −32·1)	17 939 (15 761 to 20 312)	−62·4% (−66·9 to −57·5)
Myanmar	41 374 (37 280 to 46 280)	−36·8% (−45·7 to −26·2)	64 242 (58 671 to 69 263)	−16·9% (−20·3 to −13·2)	953 822 (856 417 to 1 076 497)	−38·9% (−47·1 to −29·1)
Philippines	67 163 (58 684 to 76 412)	14·1% (−0·2 to 30·9)	100 293 (92 259 to 108 067)	15·0% (9·8 to 20·7)	1 726 655 (1 507 201 to 1 962 345)	15·7% (1·1 to 32·5)
Sri Lanka	11 010 (8982 to 13 339)	−35·9% (−48·3 to −21·4)	27 450 (25 024 to 29 855)	−8·3% (−12·6 to −3·8)	221 114 (183 518 to 264 238)	−28·3% (−41·2 to −13·3)
Seychelles	47 (41 to 54)	−37·7% (−46·5 to −28·0)	123 (112 to 133)	−10·1% (−14·1 to −5·7)	1109 (965 to 1276)	−38·0% (−47·2 to −28·0)
Thailand	36 819 (33 056 to 41 334)	−51·5% (−57·1 to −45·4)	98 499 (89 733 to 107 097)	−18·3% (−21·9 to −14·2)	874 535 (773 422 to 982 234)	−47·3% (−53·6 to −40·9)
Timor-Leste	570 (447 to 718)	−32·2% (−47·8 to −5·2)	965 (879 to 1052)	−7·9% (−11·6 to −3·8)	13 083 (10 095 to 16 518)	−36·9% (−52·1 to −13·9)
Vietnam	102 745 (92 847 to 113 544)	−33·0% (−43·2 to −19·7)	127 433 (118 570 to 136 677)	−21·1% (−24·4 to −17·8)	1 967 359 (1 755 905 to 2 225 169)	−34·6% (−44·9 to −21·4)
**Oceania**	**8675 (7362 to 10 038)**	**−19·8% (−29·9 to −7·4)**	**12 562 (11 607 to 13 480)**	**−8·1% (−11·0 to −5·2)**	**272 605 (230 796 to 319 285)**	**−18·6% (−30·0 to −5·3)**
American Samoa	27 (23 to 32)	−33·3% (−43·7 to −20·3)	76 (69 to 82)	−12·4% (−16·0 to −8·2)	831 (700 to 973)	−33·3% (−43·6 to −21·0)
Federated States of Micronesia	100 (79 to 125)	−17·5% (−34·7 to 3·7)	123 (113 to 133)	−8·9% (−12·6 to −4·5)	2572 (2025 to 3265)	−17·6% (−36·6 to 5·1)
Fiji	569 (449 to 710)	−21·3% (−40·0 to 3·4)	1176 (1069 to 1280)	−6·5% (−10·8 to −2·2)	15 533 (12 384 to 19 147)	−21·1% (−39·5 to 4·1)
Guam	121 (107 to 138)	−9·0% (−22·6 to 6·7)	294 (271 to 320)	1·6% (−2·7 to 6·5)	2903 (2550 to 3304)	−6·4% (−19·4 to 9·1)
Kiribati	113 (99 to 128)	−14·0% (−25·3 to −0·7)	139 (128 to 150)	−8·7% (−12·5 to −4·7)	3442 (2994 to 3937)	−13·3% (−25·5 to 1·3)
Marshall Islands	42 (35 to 50)	−18·6% (−30·6 to −4·4)	69 (63 to 74)	−5·4% (−9·5 to −1·1)	1312 (1097 to 1543)	−16·6% (−29·3 to −1·6)
Northern Mariana Islands	25 (20 to 31)	−36·7% (−49·5 to −20·3)	80 (72 to 89)	−14·5% (−17·9 to −10·6)	905 (727 to 1103)	−34·4% (−47·7 to −18·0)
Papua New Guinea	6620 (5320 to 7980)	−18·1% (−31·3 to −0·3)	7867 (7271 to 8436)	−8·5% (−12·1 to −4·8)	214 129 (172 917 to 260 821)	−19·8% (−33·7 to −2·0)
Samoa	136 (112 to 159)	−29·0% (−40·8 to −16·6)	234 (217 to 253)	−11·9% (−15·6 to −7·9)	3132 (2588 to 3696)	−29·7% (−41·4 to −17·1)
Solomon Islands	578 (481 to 703)	−13·1% (−26·3 to 3·4)	625 (577 to 671)	−8·7% (−12·2 to −4·8)	16 739 (13 771 to 20 527)	−13·1% (−27·9 to 7·3)
Tonga	57 (50 to 65)	−21·8% (−35·5 to −5·1)	115 (106 to 124)	−4·9% (−8·7 to −1·2)	1257 (1087 to 1436)	−22·0% (−35·6 to −6·3)
Vanuatu	287 (233 to 351)	−13·4% (−28·0 to 3·5)	345 (318 to 371)	−11·5% (−15·4 to −7·4)	8226 (6663 to 10 200)	−13·2% (−28·8 to 5·9)
**North Africa and Middle East**	**238 747 (219 467 to 259 910)**	**−23·8% (−29·3 to −15·9)**	**586 080 (535 384 to 637 793)**	**−6·0% (−9·0 to −2·9)**	**5 655 638 (5 205 166 to 6 152 964)**	**−26·8% (−31·3 to −20·8)**
Afghanistan	23 132 (18 798 to 27 674)	1·9% (−9·7 to 18·1)	27 042 (24 921 to 29 329)	−3·3% (−7·5 to 1·3)	650 879 (528 442 to 797 591)	−1·8% (−14·0 to 12·8)
Algeria	16 682 (14 193 to 19 374)	−31·2% (−39·5 to −21·4)	44 590 (40 627 to 48 799)	−12·9% (−17·2 to −8·3)	332 855 (288 218 to 383 258)	−37·4% (−44·6 to −28·7)
Bahrain	139 (114 to 169)	−53·1% (−61·9 to −42·3)	937 (834 to 1041)	−16·7% (−21·0 to −12·3)	4213 (3538 to 5059)	−54·1% (−61·9 to −44·5)
Egypt	52 093 (44 633 to 60 475)	−26·9% (−36·8 to −13·3)	107 854 (98 437 to 117 547)	−1·2% (−6·2 to 4·3)	1 193 756 (1 027 522 to 1 387 332)	−24·2% (−34·1 to −11·9)
Iran	28 786 (24 530 to 33 892)	−28·2% (−42·4 to −9·8)	82 516 (74 457 to 90 102)	−15·8% (−20·0 to −11·3)	621 007 (529 942 to 723 486)	−30·8% (−43·9 to −13·5)
Iraq	17 080 (14 064 to 20 294)	−16·4% (−32·9 to 2·3)	33 390 (30 583 to 36 358)	−8·8% (−13·0 to −3·3)	457 517 (377 396 to 551 247)	−18·2% (−35·1 to 0·9)
Jordan	1991 (1573 to 2518)	−42·1% (−54·6 to −26·8)	6302 (5729 to 6895)	−18·0% (−22·9 to −12·9)	42 916 (34 918 to 53 263)	−44·2% (−56·2 to −29·7)
Kuwait	445 (335 to 580)	11·7% (−16·1 to 46·1)	2655 (2361 to 2952)	14·8% (8·2 to 21·8)	14 354 (11 318 to 18 114)	5·5% (−16·9 to 31·6)
Lebanon	1174 (895 to 1543)	−68·1% (−76·7 to −55·9)	6745 (6098 to 7450)	−19·1% (−23·8 to −14·2)	25 835 (20 765 to 31 702)	−68·5% (−75·8 to −59·0)
Libya	1914 (1582 to 2353)	−12·7% (−25·6 to 1·5)	6526 (5890 to 7122)	7·8% (2·5 to 13·7)	48 552 (40 901 to 58 338)	−28·0% (−41·4 to −14·3)
Morocco	15 730 (12 655 to 19 431)	−25·4% (−36·3 to −6·1)	42 777 (38 974 to 46 792)	−4·1% (−8·4 to 0·5)	328 748 (269 767 to 398 089)	−30·9% (−39·5 to −17·6)
Oman	755 (670 to 839)	−46·4% (−57·9 to −33·3)	3049 (2750 to 3363)	−6·5% (−10·2 to −2·6)	23 383 (20 770 to 26 003)	−58·6% (−67·6 to −48·0)
Palestine	2240 (2076 to 2407)	20·3% (3·8 to 41·3)	3580 (3288 to 3882)	17·8% (11·0 to 24·5)	44 791 (41 551 to 48 570)	5·8% (−8·8 to 24·3)
Qatar	125 (92 to 169)	−62·7% (−71·8 to −50·2)	1287 (1132 to 1449)	−16·5% (−21·3 to −11·6)	5700 (4436 to 7277)	−59·7% (−68·4 to −47·9)
Saudi Arabia	8539 (7730 to 9449)	−24·5% (−36·8 to −8·7)	26 252 (23 787 to 28 744)	−8·0% (−10·8 to −5·0)	187 808 (169 663 to 208 145)	−27·7% (−38·4 to −14·8)
Sudan	16 573 (14 063 to 19 353)	−25·7% (−34·1 to −16·0)	32 782 (30 033 to 35 601)	−8·7% (−13·0 to −4·3)	451 852 (379 302 to 537 310)	−31·9% (−39·0 to −24·1)
Syria	5305 (4777 to 5905)	−45·1% (−52·0 to −36·2)	15 063 (13 786 to 16 378)	−17·6% (−21·4 to −13·4)	148 597 (134 086 to 165 724)	−49·2% (−55·6 to −41·4)
Tunisia	5959 (4729 to 7333)	−31·2% (−43·6 to −16·2)	16 076 (14 527 to 17 600)	−5·8% (−10·7 to −1·0)	108 182 (87 754 to 131 512)	−34·6% (−45·8 to −22·0)
Turkey	25 495 (21 374 to 30 226)	−17·9% (−33·6 to 1·8)	98 038 (88 873 to 107 510)	3·2% (−1·4 to 7·6)	537 614 (463 673 to 622 616)	−29·1% (−41·3 to −15·1)
United Arab Emirates	2413 (1892 to 3051)	−25·3% (−43·2 to −0·6)	7658 (6813 to 8517)	−20·3% (−24·0 to −16·4)	91 090 (71 446 to 115 501)	−26·8% (−44·1 to −2·8)
Yemen	12 175 (10 243 to 14 120)	−21·3% (−32·1 to −6·8)	20 429 (18 633 to 22 160)	−11·5% (−15·5 to −7·0)	335 469 (280 251 to 395 119)	−28·9% (−38·4 to −16·8)
**South Asia**	**954 892 (901 599 to 1 007 622)**	**−22·1% (−29·6 to −14·7)**	**1 528 321 (1 406 280 to 1 653 049)**	**−4·6% (−7·1 to −2·2)**	**22 220 182 (21 095 366 to 23 345 059)**	**−23·8% (−30·3 to −17·7)**
Bangladesh	126 369 (113 890 to 139 529)	−26·8% (−37·1 to −13·9)	161 709 (148 953 to 173 601)	−11·4% (−15·5 to −6·9)	2 871 080 (2 572 931 to 3 206 620)	−22·5% (−33·4 to −8·7)
Bhutan	301 (250 to 358)	−47·5% (−56·7 to −35·7)	578 (528 to 628)	−18·7% (−22·3 to −14·8)	6343 (5197 to 7628)	−49·9% (−59·1 to −39·2)
India	694 144 (647 980 to 737 239)	−23·7% (−31·2 to −15·3)	1 175 778 (1 076 048 to 1 274 427)	−3·2%–5·6 to −0·7)	16 354 773 (15 392 109 to 17 294 326)	−25·8% (−32·0 to −18·8)
Nepal	14 916 (12 892 to 17 155)	−28·4% (−40·3 to −13·7)	25 307 (23 067 to 27 512)	−6·1% (−10·2 to −2·1)	329 988 (287 815 to 377 913)	−32·9% (−43·4 to −20·4)
Pakistan	119 162 (101 972 to 137 535)	−11·7% (−27·1 to 6·8)	164 948 (150 514 to 179 264)	−8·9% (−13·5 to −4·6)	2 657 998 (2 262 441 to 3 087 650)	−13·9% (−28·3 to 4·3)
**Southern sub-Saharan Africa**	**33 545 (31 364 to 35 578)**	**−3·8% (−11·4 to 6·0)**	**62 096 (56 947 to 67 170)**	**1·9% (−0·5 to 4·7)**	**773 257 (720 981 to 823 722)**	**−7·3% (−14·7 to 1·8)**
Botswana	910 (453 to 1287)	−25·2% (−61·1 to 6·8)	1565 (1418 to 1714)	−10·6% (−14·9 to −5·9)	20 705 (10 646 to 29 571)	−25·5% (−59·9 to 8·1)
Lesotho	1752 (1298 to 2277)	16·3% (−13·4 to 53·4)	1610 (1472 to 1749)	4·9% (0·1 to 9·5)	36 219 (26 956 to 47 080)	19·3% (−12·1 to 61·2)
Namibia	937 (628 to 1217)	−43·8% (−60·8 to −28·3)	1588 (1453 to 1731)	−26·4% (−30·4 to −21·9)	19 827 (13 523 to 25 655)	−47·3% (−63·1 to −32·2)
South Africa	23 906 (22 357 to 25 503)	−0·3% (−8·7 to 9·7)	48 260 (44 245 to 52 262)	2·1% (−0·5 to 4·9)	511 038 (478 470 to 543 233)	−10·8% (−18·3 to −3·1)
Swaziland	581 (380 to 819)	−23·0% (−45·5 to 3·5)	822 (746 to 901)	−6·9% (−11·4 to −1·5)	12 717 (8431 to 17 898)	−23·0% (−46·1 to 5·4)
Zimbabwe	5459 (4438 to 6613)	−11·3% (−32·3 to 37·1)	82517508 to 8958)	5·1% (0·5 to 9·7)	172 751 (144 742 to 204 256)	9·6% (−15·8 to 73·3)
**Western sub-Saharan Africa**	**105 939 (96 170 to 114 435)**	**−18·2% (−26·0 to −10·0)**	**202 647 (185 544 to 220 626)**	**−5·7% (−8·7 to −2·3)**	**2 890 623 (2 631 129 to 3 124 294)**	**−20·6% (−28·0 to −13·2)**
Benin	4062 (3493 to 4629)	−5·0% (−17·3 to 8·8)	6181 (5680 to 6685)	−3·1% (−7·4 to 1·5)	109 438 (94 376 to 124 293)	−8·0% (−19·5 to 4·6)
Burkina Faso	4314 (3615 to 5012)	11·3% (−5·3 to 33·7)	79857260 to 8772)	9·8% (5·3 to 14·8)	117 917 (99 438 to 136 783)	4·6% (−10·6 to 25·4)
Cameroon	9091 (6846 to 11 638)	−3·6% (−24·2 to 20·2)	12 801 (11 739 to 13 890)	−3·5% (−8·1 to 1·0)	234 925 (175 653 to 302 530)	−1·2% (−23·2 to 24·0)
Cape Verde	216 (185 to 246)	−35·4% (−44·4 to −25·7)	426 (391 to 464)	−14·1% (−18·0 to −9·7)	4472 (3814 to 5134)	−38·2% (−47·3 to −27·7)
Chad	4259 (3544 to 5002)	−11·2% (−24·5 to 4·5)	6947 (6344 to 7512)	−2·0% (−6·2 to 2·7)	121 309 (101 074 to 143 343)	−11·9% (−24·6 to 2·5)
Côte d'Ivoire	10 788 (9213 to 12 430)	−4·0% (−16·2 to 10·4)	14 502 (13 329 to 15 718)	−7·3% (−11·2 to −2·9)	306 552 (261 505 to 358 094)	−4·3% (−16·3 to 10·5)
The Gambia	477 (406 to 550)	−14·0% (−28·2 to 1·9)	992 (908 to 1078)	−3·0% (−7·0 to 1·1)	13 083 (11 171 to 15 064)	−15·8% (−29·3 to −1·6)
Ghana	13 922 (11 821 to 15 825)	−13·3% (−28·7 to 5·6)	18 747 (17 257 to 20 270)	−6·9% (−10·9 to −2·3)	334 213 (286 593 to 380 605)	−16·1% (−30·4 to 1·7)
Guinea	5774 (4909 to 6687)	1·9% (−13·9 to 18·9)	7644 (7036 to 8298)	−0·3% (−4·5 to 4·1)	153 562 (132 282 to 178 334)	−0·9% (−16·3 to 15·7)
Guinea-Bissau	1145 (973 to 1339)	−10·8% (−24·9 to 5·6)	1323 (1216 to 1429)	−7·6% (−11·2 to −3·5)	31 694 (26 818 to 37 178)	−13·4% (−26·4 to 3·3)
Liberia	1647 (1454 to 1857)	0·1% (−13·7 to 16·6)	2650 (2443 to 2870)	0·8% (−3·5 to 5·5)	42 873 (37 820 to 47 988)	−5·0% (−18·0 to 9·6)
Mali	5168 (4167 to 6326)	−31·6% (−44·1 to −15·2)	8345 (7620 to 9098)	−14·1% (−18·0 to −9·9)	149 767 (122 153 to 180 785)	−34·7% (−47·1 to −18·7)
Mauritania	1111 (829 to 1464)	−40·3% (−52·9 to −25·3)	2444 (2225 to 2660)	−19·0% (−22·9 to −14·9)	28 170 (21 364 to 36 556)	−43·3% (−54·8 to −29·3)
Niger	6315 (4566 to 7927)	−9·7% (−27·6 to 11·0)	10 280 (9390 to 11 227)	−2·5% (−6·9 to 2·0)	180 124 (133 201 to 222 513)	−14·1% (−31·2 to 6·5)
Nigeria	27 031 (21 600 to 32 789)	−39·4% (−51·2 to −26·7)	84 197 (76 406 to 92 679)	−9·5% (−13·7 to −4·8)	771 086 (640 133 to 917 945)	−40·9% (−52·0 to −28·8)
São Tomé and Príncipe	86 (73 to 100)	−4·1% (−20·6 to 12·8)	127 (117 to 138)	−6·6% (−10·8 to −2·0)	1921 (1597 to 2254)	−12·4% (−27·9 to 5·3)
Senegal	5259 (4531 to 6069)	−0·1% (−11·6 to 13·5)	8966 (8220 to 9725)	0·2% (−4·4 to 4·8)	136 173 (118 011 to 156 851)	−2·4% (−13·2 to 9·8)
Sierra Leone	2499 (2143 to 2841)	5·8% (−8·6 to 23·8)	3870 (3529 to 4210)	4·7% (0·0 to 9·6)	76 367 (65 850 to 86 752)	2·0% (−11·7 to 18·7)
Togo	2775 (2286 to 3280)	−8·5% (−21·6 to 6·4)	4213 (3852 to 4569)	−3·9% (−8·4 to 0·5)	76 969 (64 200 to 90 976)	−8·4% (−21·0 to 6·1)
**Eastern sub-Saharan Africa**	**133 526 (121 230 to 147 971)**	**−35·6% (−41·9 to −27·0)**	**192 119 (176 247 to 208 292)**	**−16·5% (−19·2 to −13·8)**	**3 189 024 (2 910 872 to 3 535 592)**	**−38·2% (−44·3 to −30·8)**
Burundi	4421 (3685 to 5323)	−50·6% (−60·3 to −38·2)	5425 (4961 to 5915)	−31·0% (−34·2 to −27·5)	110 743 (92 014 to 132 609)	−54·7% (−64·1 to −43·0)
Comoros	219 (177 to 273)	−48·1% (−57·5 to −36·8)	388 (354 to 422)	−26·9% (−30·8 to −23·0)	5463 (4419 to 6790)	−52·1% (−60·6 to −41·5)
Djibouti	327 (234 to 437)	−30·6% (−47·2 to −12·4)	588 (536 to 640)	−11·1% (−15·2 to −6·6)	7818 (5554 to 10 582)	−33·6% (−49·6 to −16·9)
Eritrea	1949 (1582 to 2419)	−45·4% (−53·6 to −36·2)	2358 (2154 to 2577)	−24·9% (−28·5 to −20·7)	48 369 (39 196 to 60 145)	−49·8% (−57·3 to −40·7)
Ethiopia	38 353 (32 250 to 45 098)	−44·2% (−54·0 to −29·1)	52 548 (48 042 to 57 217)	−20·9% (−24·5 to −17·1)	887 620 (747 055 to 1 042 900)	−47·4% (−56·8 to −33·9)
Kenya	10 570 (8568 to 13 029)	−19·6% (−30·0 to −4·2)	21 107 (19 206 to 23 058)	−4·0% (−6·4 to −1·7)	248 359 (203 981 to 303 376)	−20·4% (−30·1 to −7·4)
Madagascar	16 862 (13 152 to 20 830)	−15·3% (−33·0 to 2·9)	15 98714 696 to 17 266)	−18·0% (−21·9 to −13·9)	448 528 (351 040 to 560 459)	−18·1% (−34·7 to 0·2)
Malawi	5080 (3969 to 6256)	−24·6% (−41·8 to −2·2)	8704 (7879 to 9516)	−9·4% (−13·6 to −5·1)	114 628 (90 200 to 140 823)	−27·5% (−44·3 to −6·1)
Mozambique	12 066 (10 024 to 14 568)	−35·5% (−47·0 to −21·1)	16 786 (15 330 to 18 225)	−15·7% (−19·9 to −11·6)	276 356 (229 481 to 334 354)	−37·1% (−48·5 to −23·3)
Rwanda	3028 (2349 to 3750)	−59·3% (−69·1 to −48·3)	5051 (4574 to 5524)	−34·0% (−37·3 to −30·5)	70 174 (54 910 to 87 266)	−63·3% (−72·7 to −52·6)
Somalia	5247 (4258 to 6507)	−28·2% (−39·2 to −14·9)	5824 (5329 to 6304)	−16·3% (−20·1 to −12·7)	130 125 (105 830 to 161 583)	−31·7% (−42·6 to −17·0)
South Sudan	4774 (3580 to 6099)	−18·5% (−35·6 to 2·9)	7207 (6587 to 7878)	−9·8% (−13·8 to −5·6)	114 261 (87 008 to 146 428)	−22·2% (−39·1 to 0·1)
Tanzania	14 647 (11 980 to 17 354)	−30·4% (−42·8 to −16·0)	26 556 (24 322 to 28 845)	−8·7% (−12·8 to −4·4)	344 995 (285 299 to 407 059)	−32·3% (−44·2 to −18·1)
Uganda	10 409 (8568 to 12 321)	−38·8% (−49·6 to −25·7)	15 359 (14 022 to 16 747)	−20·0% (−23·5 to −16·4)	247 499 (205 349 to 293 208)	−42·3% (−52·8 to −29·0)
Zambia	5572 (4267 to 6974)	2·7% (−24·8 to 41·0)	8080 (7360 to 8816)	−1·8% (−6·2 to 2·6)	133 977 (103 666 to 167 385)	5·2% (−23·4 to 44·6)
**Central sub-Saharan Africa**	**47 427 (40 448 to 53 380)**	**−17·2% (−25·8 to −7·5)**	**62 468 (57 344 to 67 591)**	**−11·2% (−14·5 to −7·9)**	**1 116 581 (970 019 to 1 241 311)**	**−20·7% (−28·7 to −11·6)**
Angola	7656 (5801 to 10 190)	−29·0% (−45·4 to −6·8)	12 096 (11 051 to 13 153)	−15·7% (−19·4 to −11·9)	190 746 (144 242 to 253 969)	−33·5% (−49·4 to −11·7)
Central African Republic	4110 (3178 to 5045)	−7·5% (−24·9 to 9·9)	3772 (3459 to 4083)	−9·1% (−13·4 to −4·7)	97 536 (76 289 to 120 239)	−8·4% (−26·5 to 10·6)
Congo Brazzaville)	2139 (1678 to 2635)	−41·7% (−54·6 to −27·1)	2978 (2726 to 3239)	−25·2% (−29·2 to −21·2)	48 013 (37 480 to 59 329)	−45·3% (−57·6 to −30·9)
Democratic Republic of the Congo	32 497 (26 643 to 37 306)	−9·0% (−20·0 to 3·6)	41 815 (38 416 to 45 399)	−7·2% (−11·3 to −2·7)	759 216 (636 168 to 865 958)	−12·3% (−22·8 to −0·1)
Equatorial Guinea	190 (112 to 287)	−70·7% (−81·2 to −56·9)	510 (461 to 560)	−31·1% (−34·8 to −26·8)	4553 (2821 to 6760)	−73·7% (−83·2 to −61·0)
Gabon	834 (671 to 1008)	−37·8% (−49·6 to −22·6)	1297 (1189 to 1419)	−24·0% (−27·6 to −20·2)	16 518 (13 375 to 19 884)	−41·3% (−53·1 to −26·4)

DALYs=disability-adjusted life-years. SDI=Socio-demographic Index.

**Figure 1:**
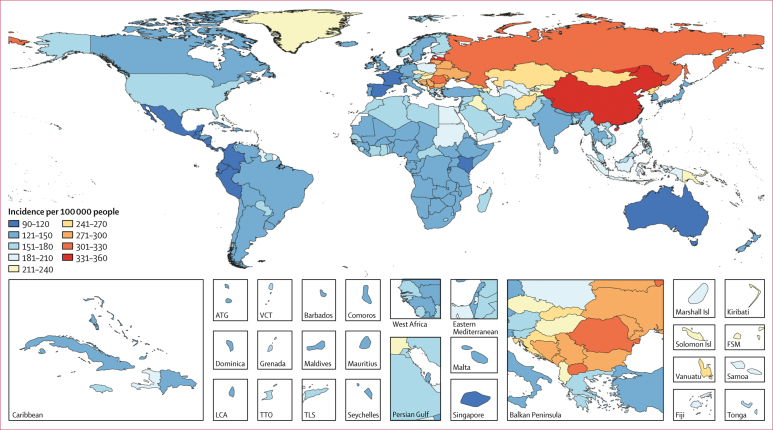
Age-standardised stroke incidence by country, for both sexes, 2016 ATG=Antigua and Barbuda. FSM=Federated States of Micronesia. Isl=Islands. LCA=Saint Lucia. VCT=Saint Vincent and the Grenadines. TLS=Timor-Leste. TTO=Trinidad and Tobago.

**Figure 2:**
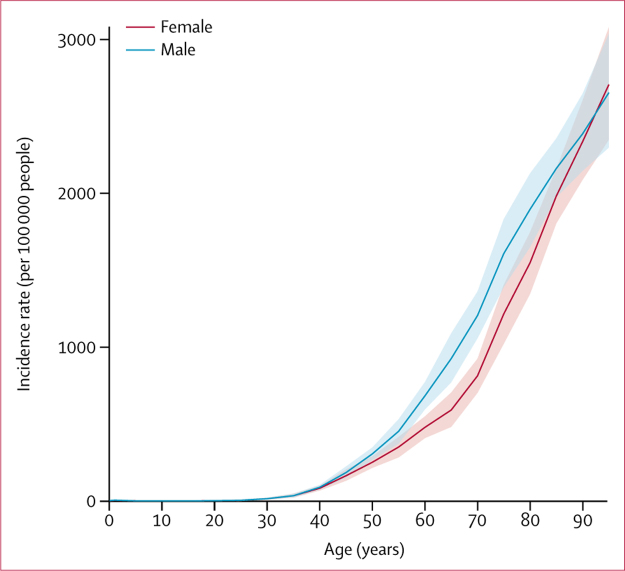
Global incidence of stroke by age and sex, 2016

The highest age-standardised incidences of stroke were observed in east Asia, especially China (354 [95% UI 331–378] per 100 000 person-years), followed by eastern Europe, ranging from 200 (181–218) per 100 000 person-years in Estonia to 335 (301–369) per 100 000 person-years in Latvia (figure 1). The lowest incidences were in central Latin America, especially El Salvador (97 [88–105] per 100 000 person-years). Age-specific stroke incidence was similar between men and women younger than 55 years, but significantly greater for men than women at ages 55–75 years (figure 2).

Age-standardised incidence declined from 1990 to 2016 globally (−8·1% [–10·7 to −5·5]), in all SDI groups except the middle SDI group, and in most regions (table; appendix). The region with the largest decrease in age-standardised stroke incidence was southern Latin America (−33·3% [–36·4 to −29·7]) and the region with the largest increase was east Asia (4·9% [1·3 to 8·1]). For ischaemic stroke, the largest decrease was in southern Latin America (−38·0% [–39·4 to −36·6]), and the largest increase was in east Asia (17·5% [15·8 to 19·2]; appendix). For haemorrhagic stroke, incidence decreased in all regions. The largest decrease was in high-income Asia Pacific (−32·5% [–33·7 to −31·2]), and the smallest decrease was in southern sub-Saharan Africa (−5·1% [–6·2 to −4·0]; appendix).

Globally, the age-standardised rate of deaths due to stroke decreased by 36·2% (−39·3 to −33·6) from 1990 to 2016, with decreases in all five SDI groups. These death rates also declined for all but one region from 1990 to 2016, with the largest decrease in the high-income Asia Pacific region (−66·3% [–68·8 to −63·4]) and no significant change in southern sub-Saharan Africa (−3·8% [–11·4 to 6·0]; table). These results were similar for ischaemic stroke and haemorrhagic stroke, with the largest decrease for both in the high-income Asia Pacific region (−70·2% [–72·3 to −67·8] for ischaemic stroke and −59·8% [–63·1 to −56·1] for haemorrhagic stroke; appendix). Death rates for neither ischaemic nor haemorrhagic stroke changed significantly between 1990 and 2016 in southern sub-Saharan Africa (0·6% [–7·9 to 11·9] for ischaemic stroke and −7·2 [–15·1 to 2·2] for haemorrhagic stroke).

Age-standardised DALY rates for stroke also declined from 1990 to 2016 globally (34·2% [–37·2 to −31·5]) for all SDI quintiles, and for all regions, again with the largest change occurring in high-income Asia Pacific (−61·5% [–64·9 to −58·1]) and the smallest in southern sub-Saharan Africa (−7·3% [–14·7 to 1·8]; table). Southern Latin America was the region with the largest decrease for ischaemic stroke (−63·7% [–66·8 to −60·6]; appendix) and high-income Asia Pacific was the region with the largest decrease for haemorrhagic stroke (−59·9% [–63·4 to −55·8]; appendix). Southern sub-Saharan Africa had no change in ischaemic stroke (−1·9% [–9·9 to 7·4]) and a decrease in haemorrhagic stroke (−10·2% [–17·9 to −1·1]).

**Figure 3:**
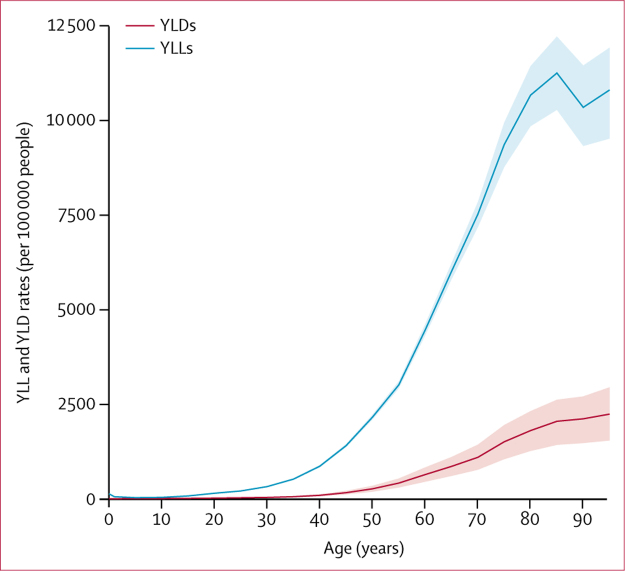
Age-standardised rates of YLLs and YLDs due to stroke for both sexes, by age, 2016 YLDs=years lived with disability. YLLs=years of life lost.

Rates of YLLs and YLDs were very low for the younger age groups (<40 years) and then increased substantially with age, with YLLs increasing much more rapidly than YLDs (figure 3) because of the high mortality burden of stroke.

**Figure 4:**
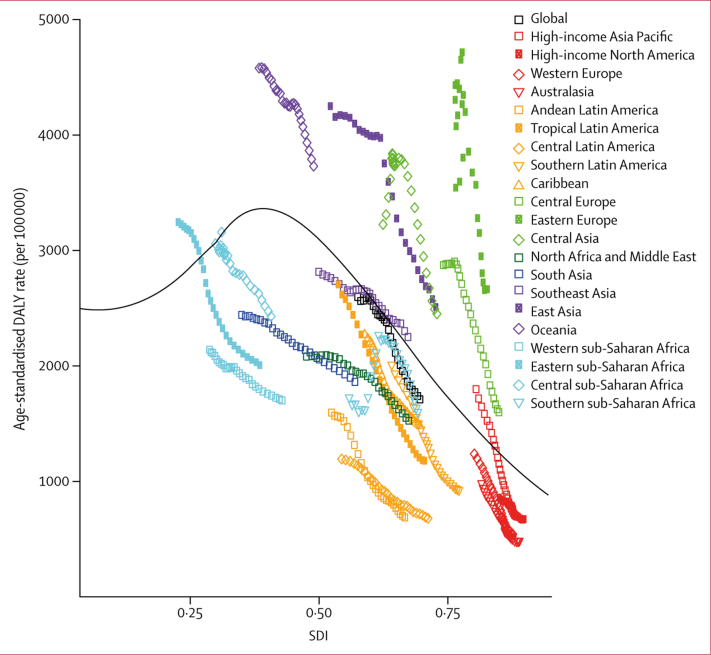
Age-standardised DALY rates for stroke versus SDI for both sexes, by region, 1990–2016 Black line shows the expected values by SDI based on a regression of all years of data for all GBD locations between 1990 and 2016. DALY=disability-adjusted life-year. SDI=Socio-demographic Index.

After an increase in expected DALY rates at the lower end of the SDI scale, these rates decline rapidly for SDI values of 0·35 and higher (figure 4). For most regions, the burden of stroke decreased with increases in SDI over time. However, central and eastern Europe and central Asia saw increased DALY rates in the early 1990s after the dissolution of the Soviet Union, followed by subsequent decreases as SDI increased. Stroke DALY burden in southern sub-Saharan Africa showed a similar pattern, with an initial spike in rates with increasing SDI, followed by a steady decrease. DALY rates were higher than expected early in the time series for eastern and central sub-Saharan Africa and high-income Asia Pacific but have decreased with increasing SDI. Eastern Europe, central Europe, central Asia, Oceania, and east Asia had higher stroke-related DALY rates than would be expected on the basis of comparisons of SDI for all years. Conversely, rates for Latin America, western Europe, Australasia, south Asia, and southern and western sub-Saharan Africa were lower than expected for all timepoints. Although DALY rates in southeast Asia were initially lower than expected, they were in most recent years slightly higher than expected.

Under the comparative risk assessment framework, most stroke DALYs (88·8% [95% UI 86·5–90·9]) can be attributed to risk factors measured in GBD; this percentage is similar for both stroke types (87·9% for ischaemic stroke [84·1–91·6] and 89·5% for haemorrhagic stroke [87·1–91·6]). Metabolic risks (high systolic blood pressure, high body-mass index, high fasting plasma glucose, high total cholesterol, and low glomerular filtration rate) accounted for 72·1% (66·4–77·3) of stroke DALYs. Behavioural factors (smoking, poor diet, and low physical activity) accounted for 66·3% (59·3–73·1) of attributable DALYs, and environmental risks (air pollution and lead exposure) for 28·1% (25·3–30·9). As the effect of many of these risk factors are mediated partly or wholly through another risk factor, the crude sum of the groups is expected to exceed 100%. The aggregation process to generate estimates of overall attributable burden accounts for joint effects of a combination of risk factors, thus the final estimate is less than 100%. The remaining burden is due to unknown or unmeasured risk factors, genetic factors, or the effect of gene–environment interactions. Population attributable fractions and UIs for the top ten risk factors for each stroke subtype by sex in 1990 and 2016 are in the appendix.

## Discussion

Our estimates indicate that the global burden of stroke is high, with more than 80 million stroke survivors in 2016. Age-standardised death rates from stroke have decreased in all regions from 1990 to 2016, whereas incidence has decreased in most regions but increased in east Asia and southern sub-Saharan Africa. The overall burden of stroke, as quantified by age-standardised DALY rates, decreased from 1990 to 2016, but the absolute number of DALYs due to stroke increased over that same period. The increase in absolute numbers is largely due to population growth and ageing resulting in a greater number of people with stroke despite declining incidence and improved stroke survival leading to higher prevalence of chronic stroke.

Studies have shown that much of the burden due to stroke can be attributed to modifiable atherosclerotic risk factors. INTERSTROKE,^13^ a case-control study done at 32 locations, found that the risk factors for stroke in low-income and middle-income countries were similar to those in high-income countries, although the relative contribution of each differed between regions. The high burden of stroke worldwide suggests that primary prevention strategies are either not widely implemented or not sufficiently effective. In addition to targeting behavioural risk factors, effective screening for conditions that increase stroke risk, such as hypertension, atrial fibrillation, and diabetes mellitus, is essential. Many screening strategies use the predicted absolute risk of cardiovascular disease to identify individuals at high risk of cardiovascular disease events and to define therapeutic thresholds for specific interventions.^14^, ^15^ However, these approaches have limitations, including low efficiency and missing data for people with low to moderate cardiovascular disease risk, in whom about 80% of strokes occur.^16^, ^17^ Preliminary evidence suggests that strategies via mobile technologies are effective for healthy lifestyle modification and primary stroke prevention.^18^, ^19^ Treatment with statins and blood pressure medications has been shown to be effective and cost-effective for both primary and secondary prevention of stroke.^20^, ^21^, ^22^ Healthy lifestyle modification and better adherence to recommended medications via an affordable multidrug polypill containing blood pressure and lipid-lowering medications could potentially also enable cost-effective prevention of stroke globally, potentially halving stroke incidence and mortality.^14^, ^23^, ^24^

In addition to prevention efforts, appropriate acute and long-term treatment is essential, given the high recurrence rate of stroke. Highly effective treatments for stroke have been developed over the past few decades and are now considered the standard of care where available.^25^, ^26^ To assist countries in identifying gaps in stroke care, a survey has been done in collaboration with the World Stroke Organization and WHO to obtain data on facilities and providers for acute stroke care and rehabilitation; results from the survey will be available soon.^27^ Studies are also underway to assess different approaches to treatment when the closest medical facilities do not have the resources to provide advanced stroke care (NCT02795962).

Although the attributable burden for most of the risk factors identified for stroke has been quantified in GBD, the effect of atrial fibrillation has not yet been estimated. Atrial fibrillation increases the risk of stroke up to five fold, largely through an increased risk of thrombotic events leading to ischaemic stroke.^28^ Antithrombotic therapy with vitamin K antagonists, antiplatelet drugs, or novel oral anticoagulants have been shown to reduce this risk by up to 60% in a meta-analysis of clinical trials.^29^ Improved diagnosis of and treatment for atrial fibrillation is thus likely to have a substantial impact on stroke burden.

GBD faces several measurement challenges for estimating cause-specific mortality and non-fatal burden of stroke. Although GBD employs spatiotemporal methods that use patterns across time and geographic regions to inform estimates for locations with sparse data, these approaches cannot completely overcome issues when data are missing for some large geographic regions. Although much stroke data is available for some regions, data on incidence, stroke type, and stroke severity is sparse in many low-income and middle-income countries. Adding new sources of data is an ongoing effort of GBD, but we are limited to locations where representative studies have been done or where there is access to administrative data. To ensure that inpatient hospital data capture all events, adjustments are made to these data using correction factors derived from medical claims data, to which we only had access for the USA in GBD 2016. The generalisability of claims data and the derived correction factors have been questioned.^30^, ^31^, ^32^ However, data from administrative sources provide essential information for capturing burden in many locations, and we are able to adjust the estimates during the modelling process using stroke-specific registries and studies as the reference. Using similar methods, we are also able to include data from the literature that do not meet our reference case definition of subtype-specific, first-ever stroke.

Improvements in modelling strategy are made regularly for causes in GBD, including stroke. In GBD 2016, we did not estimate the burden due to subarachnoid haemorrhage and intracerebral haemorrhage separately; however, future iterations of GBD will. We also do not generate estimates for transient ischaemic attacks; however, incidence estimates for these events would be useful for health planning purposes.^33^ The new ICD 11 classifications, which include imaging criteria, could be used along with the original clinical WHO diagnostic criteria in future estimates.^34^

Our findings with regard to stroke burden and modifiable risk factors are consistent with those from GBD 2015 and emphasise the need for effective prevention strategies. Although age-standardised deaths due to stroke have been decreasing, the overall burden of stroke remains high, continues to increase, and is unlikely to decrease without interventions to address stroke risk factors. Stroke has been identified as one of the priorities for WHO and the UN in their actions to reduce the burden of non-communicable diseases; global estimates such as those generated by GBD are essential in appropriately targeting efforts.^35^
