# Supplementary material for: Global, regional, and national burden of stroke, 1990–2016: a systematic analysis for the Global Burden of Disease Study 2016
Source: Lancet Neurol. 2019 May;18(5):439–58. doi: 10.1016/S1474-4422(19)30034-1 (PMC6494974; doi:10.1016/S1474-4422(19)30034-1)
Supplement: Supplementary appendix [file mmc1.pdf]

THE LANCET
Neurology

Supplementary appendix

This appendix formed part of the original submission and has been peer reviewed. We post it as supplied by the authors.

Supplement to: GBD 2016 Stroke Collaborators. Global, regional, and national burden of stroke, 1990–2016: a systematic analysis for the Global Burden of Disease Study 2016. *Lancet Neurol* 2019; published online March 11. http://dx.doi.org/10.1016/S1474-4422(19)30034-1.

Supplementary Methods and Results Appendix to:

Global, regional, and national burden of stroke, 1990 to 2016: a systematic analysis for the Global Burden of Disease Study 2016

Table of Contents

Supplementary Methods and Results Appendix to: 1

Summary of General Global Burden of Disease Study Methods 3

Ischaemic Stroke & Haemorrhagic Stroke 6

- Flowchart 6
- Case definition 6
- Input data 7
- Modelling strategy 10
- Changes in the modelling of stroke for GBD 2016 11

Methods Tables 14

- Table A1: GATHER checklist of information that should be included in reports of global health estimates, with description of compliance and location of information for GBD 2016. 14
- Table A2: ICD Codes used in fatal and nonfatal analysis 17
- Table A3: Selected covariates for CODEm models, overall stroke and subtypes 18
- Table A4: Counts of data points used by measure and stroke model for GBD 2016 19
- Table A5: DisMod covariates 23
- Table A6: Sequelae and disability weights for ischaemic and hemorrhagic stroke 25

Supplementary Tables and Figures 26

- Figure A1: DALYs attributable to risk factors; all ages by sex and stroke subtype for 1990 and 2016; risk factors are ranked by percent attributable fraction shown. 26
- Table A7: Incidence, deaths and DALYs for 2016 and percentage change of age-standardized rates by location for Ischaemic stroke 27
- Table A8: Incidence, deaths and DALYs for 2016 and percentage change of age-standardized rates by location for Haemorrhagic stroke 42

Summary of General Global Burden of Disease Study Methods

The Institute for Health Metrics and Evaluation with a growing collaboration of scientists produces annual updates of the Global Burden of Disease study. Estimates span the period from 1990 to the most recent completed year. By the time of the release of GBD 2016 in September 2017, there were over 2,700 collaborators in 132 countries who contributed to this global public good. Annual updates allow incorporation of new data and method improvements to ensure that the most up-to-date information is available to policy makers in a timely fashion to help make resource allocation decisions. In this analysis, we have aggregated results from GBD 2016 for 15 disease and injury outcomes that are generally cared for by neurological services. These include infectious conditions (tetanus, meningitis, encephalitis), stroke, brain and other nervous system cancers, traumatic brain injury, and spinal cord lesion which are classified outside the more narrowly defined category of neurological disorders in GBD (ie, Alzheimer's disease and other dementias, Parkinson's disease, multiple sclerosis, motor neuron disease, idiopathic epilepsy, migraine, tension-type headache, and a rest category of less common other neurological disorders). Compared to a previous analysis based on GBD 2015, we were able to add the non-fatal outcomes of traumatic brain injury and spinal cord lesion, and medication overuse headache is no longer included as a separate cause but quantified as a consequence of the underlying headache types.

In the methods section of this overview paper we present a summary of the general methods of the global burden of disease. In the accompanying disease-specific papers we concentrate on methods that are specific to each disorder. The guiding principle of GBD is to assess health loss due to mortality and disability comprehensively, where we define disability as any departure from full health. In GBD 2016, estimates were made for 195 countries and territories, and 579 subnational locations, for 27 years starting from 1990, for 23 age groups and both sexes. Deaths were estimated for 264 diseases and injuries, while prevalence and incidence were estimated for 328 diseases and injuries. In order to allow meaningful comparisons between deaths and non-fatal disease outcomes as well as between diseases, the data on deaths and prevalence are summarised in a single indicator, the disability-adjusted life-year (DALY). DALYs are the sum of years of life lost (YLLs) and years lived with disability (YLDs). YLLs are estimated as the multiplication of counts of death and a standard, "ideal", remaining life expectancy at the age of death. The standard life expectancy is derived from the lowest observed mortality rates in any population in the world greater than 5 million.[2] YLDs are estimated as the product of prevalence of individual consequences of disease (or "sequelae") times a disability weight that quantifies the relative severity of a sequela as a number between zero (representing "full health") and 1 (representing death). Disability weights have been estimated in nine population surveys and an open-access internet survey in which respondents are asked to choose the "healthier"[3] between random pairs of health states that are presented with a short description of the main features.

All-cause mortality rates are estimated from vital registration data in countries with complete coverage. For other countries, the probabilities of death before age 5 and between ages 15 and 60 are estimated from censuses and surveys asking mothers to provide a history of children ever born and those still alive, and surveys asking adults about siblings who are alive or have passed away. Using model life tables, these probabilities of death are transformed into age-specific death rates by location, year, and sex. GBD has collated a large database of cause of death data from vital registrations and verbal autopsy surveys in which relatives are asked a standard set of questions to ascertain the likely cause of death, supplemented with police and mortuary data for injury deaths in countries with no other data. For countries with vital registration data, the completeness is assessed with demographic methods based on

comparing recorded deaths with population counts between two successive censuses. The cause of death information is provided in a large number of different classification systems based on versions of the International Classification of Diseases or bespoke classifications in some countries. All data are mapped into the disease and injury categories of GBD. All classification systems contain codes that are less informative because they lack a specific diagnosis (eg, unspecified cancer) or refer to codes that cannot be underlying cause of death (eg, low back pain or senility) or are intermediate causes (eg, heart failure or sepsis). Such deaths are redistributed to more precise underlying causes of death.[4] After these redistributions and corrections for under-registration, the data are analysed in CODEm (cause of death ensemble model), a highly systematised tool that runs many different models on the same data and chooses an ensemble of models that best reflects all the available input data. Models are chosen with variations in the statistical approach ("mixed effects" of spatiotemporal Gaussian Process Regression), in the unit of analysis (rates or cause fractions), and the choice of predictive covariates. The statistical performance of all models is tested by holding out 30% of the data and checking how well a model covers the data that were held out. To enforce consistency from CODEm, the sum of all cause-specific mortality rates is scaled to that of the all-cause mortality rates in each age, sex, location, and year category.

Non-fatal estimates are based on systematic reviews of published papers and unpublished documents, survey microdata, administrative records of health encounters, registries, and disease surveillance systems. Our Global Health Data Exchange (GHDx, http://ghdx.healthdata.org/) is the largest repository of health data globally. We first set a reference case definition and/or study method that best quantifies each disease or injury or consequence thereof. If there is evidence of a systematic bias in data that used different case definitions or methods compared to reference data we adjust those data points to reflect what its value would have been if measured as the reference. This is a necessary step if one wants to use all data pertaining to a particular quantity of interest rather than choosing a small subset of data of the highest quality only. DisMod-MR 2.1, a Bayesian meta-regression tool, is our main method of analyzing non-fatal data. It is designed as a geographical cascade where a first model is run on all the world's data, which produces an initial global fit and estimates coefficients for predictor variables and the adjustments for alternative study characteristics. The global fit adjusted by the values of random effects for each of seven GBD super-regions, the coefficients on sex and country predictors, are passed down as data to a model for each super-region together with the input data for that geography. The same steps are repeated going from super-region to 21 region fits and then to 195 fits by country and where applicable a further level down to subnational units. Below the global fit, all models are run separately by sex and for six time periods: 1990, 1995, 2000, 2005, 2010, and 2016. During each fit all data on prevalence, incidence, remission (ie, cure rate) and mortality are forced to be internally consistent. For most diseases, the bulk of data on prevalence or incidence is at the disease level with fewer studies providing data on the proportions of cases of disease in each of the sequelae defined for the disease. The proportions in each sequela are pooled using DisMod-MR 2.1 or meta-analysis, or derived from analyses of patient-level datasets. The multiplication of prevalent cases for each disease sequela and the appropriate disability weight produces YLD estimates that do not yet take into account comorbidity. To correct for comorbidity, these data are used in a simulation to create hypothetical individuals in each age, sex, location, and year combination who experience no, one, or multiple sequelae simultaneously. We assume that disability weights are multiplicative rather than additive as this avoids assigning a combined disability weight value in any individual to exceed 1, ie, be worse than a

“year lost due to death”. This comorbidity adjustment leads to an average scaling down of disease-specific YLDs ranging from about 2% in young children up to 17% in oldest ages.

All our estimates of causes of death are categorical: each death is assigned to a single underlying cause. This has the attractive property that all estimates add to 100%. For risks, we use a different, “counterfactual” approach, ie, answering the question: “what would the burden have been if the population had been exposed to a theoretical minimum level of exposure to a risk”. Thus, we need to define what level of exposure to a risk factor leads to the lowest amount of disease. We then analyse data on the prevalence of exposure to a risk and derive relative risks for any risk-outcome pair for which we find sufficient evidence of a causal relationship. Prevalence of exposure is estimated in DisMod-MR 2.1, using spatiotemporal Gaussian Process Regression, or from satellite imagery in the case of ambient air pollution. Relative risk data are pooled using meta-analysis of cohort, case-control and/or intervention studies. For each risk and outcome pair, we evaluate the evidence and judge if the evidence falls into the categories of “convincing” or “probable” as defined by the World Cancer Research Fund.[5] From the prevalence and relative risk results, population attributable fractions are estimated relative to the theoretical minimum risk exposure level (TMREL). When we aggregate estimates for clusters of risks, eg, metabolic or behavioural risks, we use a multiplicative function rather than simple addition and take into account how much of each risk is mediated through another risk. For instance, some of the risk of high body mass index is directly onto stroke as an outcome but much of its impact is mediated through high blood pressure, high cholesterol, or high fasting plasma glucose, and we would not want to double count the mediated effects when we estimate aggregates across risk factors.

Uncertainty is propagated throughout all these calculations by creating 1,000 values for each prevalence, death, YLL, YLD, or DALY estimate and performing aggregations across causes and locations at the level of each of the 1,000 values for all intermediate steps in the calculation. The lower and upper bounds of the 95% uncertainty interval are the 25th and 975th values of the ordered 1,000 values. For all age-standardised rates, GBD uses a standard population calculated as the non-weighted average across all countries of the percentage of the population in each five-year age group for the years 2010 to 2035 from the United Nations Population Division’s World Population Prospects (2012 revision).

GBD uses a composite indicator or sociodemographic development, SDI, which reflects the geometric mean of normalised values of a location’s income per capita, the average years of schooling in the population 15 and over, and the total fertility rate. Countries and territories are grouped into five quintiles of high, high-middle, middle, low-middle, and low SDI based on their 2016 values.

Ischaemic Stroke & Haemorrhagic Stroke

Flowchart

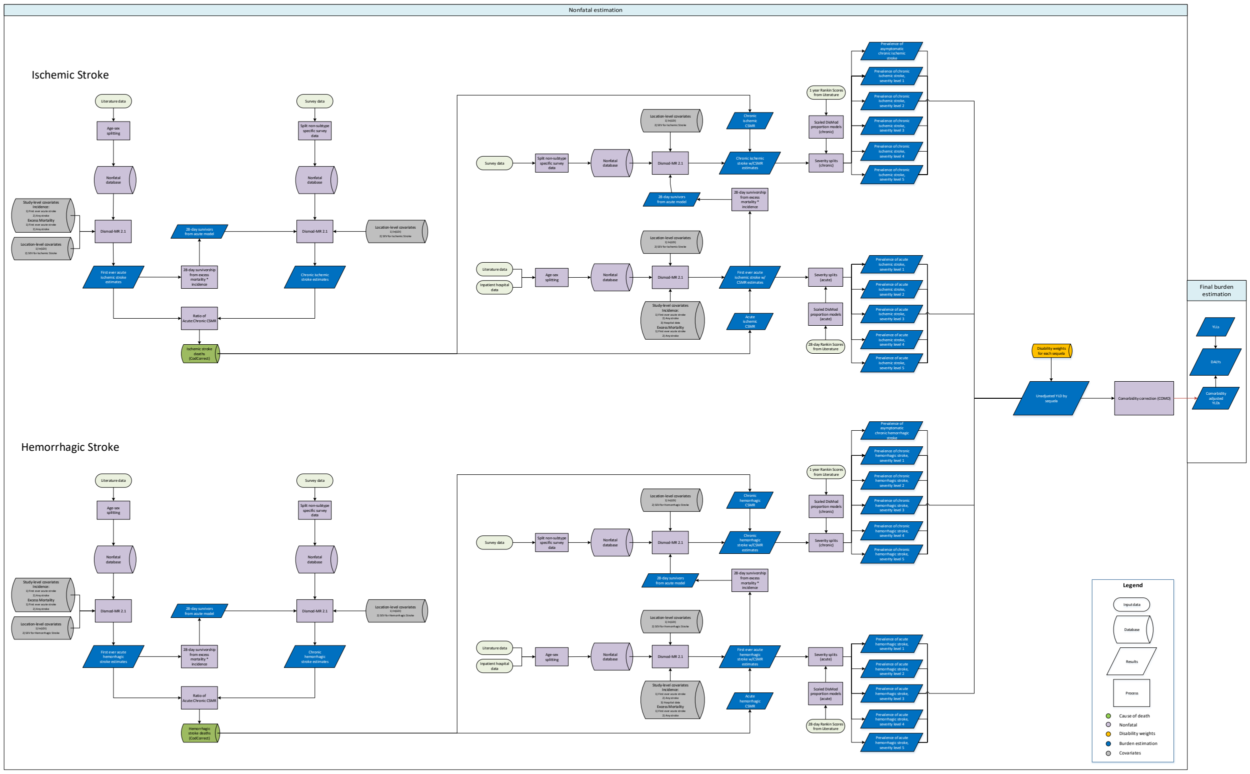

Input data and methodological summary

Case definition

Stroke was defined according to WHO criteria – rapidly developing clinical signs of focal (at times global) disturbance of cerebral function lasting more than 24 hours or leading to death with no apparent cause other than that of vascular origin(1). Data on transient ischaemic attack (TIA) were not included.

> *Acute stroke:* Stroke cases are considered acute from the day of incidence of a first-ever stroke through day 28 following the event.
>
> *Chronic stroke:* Stroke cases are considered chronic beginning 28 days following the occurrence of an event. Chronic stroke includes the sequelae of an acute stroke AND all recurrent stroke events. GBD 2015 adopts this broader definition of chronic stroke than was used in prior iterations in order to model acute strokes using only first-ever incident events.
>
> *Ischaemic stroke:* Incident ischaemic stroke is defined as the occurrence of first-ever ischaemic stroke, based on clinical diagnosis by a physician using diagnostic imaging. Ischaemic strokes are considered to include all vascular events leading to limited blood flow to brain tissue, with resulting infarction, including atherosclerotic and thromboembolic strokes but excluding strokes in which the underlying cause is intracranial haemorrhage.

Haemorrhagic or other strokes: This cause includes all non-ischaemic strokes of a vascular cause including subarachnoid and stroke due to intracranial haemorrhage.

ICD codes used for inclusion of hospital and claims data can be found in Appendix Table 4

Input data

Model inputs

A systematic review was not performed for GBD 2016. Updates to systematic reviews are performed on an ongoing schedule across all GBD causes; an update for cerebrovascular disease will be performed in the next iteration.

A systematic review of the literature was performed in GBD 2013. The search terms used were: (stroke[Mesh]) AND (prevalence[Title/Abstract] OR incidence[Title/Abstract]) AND ("2010"[Date - Publication] : "3000"[Date - Publication]) AND (hasabstract[text] AND Humans[Mesh] AND middle age[MeSH])) (hasabstract[text] AND Humans[Mesh] AND middle age[MeSH])) OR 21) AND ((hemorrhagic stroke/epidemiology[Mesh] OR hemorrhagic stroke/mortality[Mesh]) AND (prevalence[Title/Abstract] OR incidence[Title/Abstract]) AND ("2010"[Date - Publication] : "3000"[Date - Publication]) AND (hasabstract[text] AND Humans[Mesh] AND middle age[MeSH]))

The tables below indicate the number of literature studies included in GBD 2016, as well as the number of countries or subnational units and GBD world regions represented.

Acute Ischaemic stroke

	Prevalence	Incidence	Mortality risk
Studies	0	73	14
Countries/subnationals	0	55	12
GBD world regions	0	14	5

Acute Haemorrhagic or other stroke

	Prevalence	Incidence	Mortality risk
Studies	0	73	10
Countries/subnationals	0	51	11
GBD world regions	0	13	4

Chronic Ischaemic stroke

	Prevalence	Incidence	Mortality risk
Studies	53	0	8
Countries/subnationals	50	0	4
GBD world regions	14	0	2

Chronic Haemorrhagic or other stroke

	Prevalence	Incidence	Mortality risk
Studies	53	0	8
Countries/subnationals	50	0	4
GBD world regions	14	0	2

We included inpatient hospital data, adjusted for readmission and primary to any diagnosis using correction factors estimated from US claims data. We excluded data for locations where the data points were implausibly low (Vietnam, Philippines, India). In addition, we included unpublished stroke registry data for acute ischaemic and acute haemorrhagic strokes. We also included survey data for chronic cerebrovascular disease. These surveys were identified based on expert opinion and review of major survey series focused on world health that included questions regarding self-reported history of stroke.

As with many models in GBD, the diversity of data sources available means that we needed to adjust available data to our preferred or reference case definition (2). For the first ever acute stroke models we used DisMod to estimate the statistical association between measurements taken using different case definitions and then used these estimates to adjust the non-referent datapoints. We included study-level covariates to adjust data points for first and recurrent strokes combined, using data for first strokes only as reference. We also included study-level covariates to adjust ischaemic and haemorrhagic strokes combined (all stroke), using as reference studies with subtype-specific information.

Severity split inputs

The table below illustrates the severity level, lay description, and disability weights for GBD 2016. In previous iterations of the GBD, severity splits for stroke were based on the standard approach described elsewhere (3). For GBD 2016, we undertook a review to identify epidemiologic literature which reported the degree of disability at 28 days (for acute stroke) or one year (for chronic stroke) using the modified Rankin scale (mRS) and the Mini-mental State Examination (MMSE) or the Montreal Cognitive Assessment (MoCA). The mRS assesses functional capabilities, while the MMSE and MoCA tests provide evaluations of cognitive functioning. We then mapped these measures to the existing GBD categories as indicated below. This appproach allowed us to include location-specific information and can be updated as more data on functional or cognitive status become available.

Acute stroke severity splits

Severity level	Lay description	Modified Rankin Score	Cognitive Status	DW (95% CI)
Stroke, mild	Has some difficulty in moving around and some weakness in one hand, but is able to walk without help.	1	N/A	0.019 (0.01–0.032)
Stroke, moderate	Has some difficulty in moving around, and in using the hands for lifting and holding things, dressing, and grooming.	2, 3	MoCA>=24 or MMSE>=26	0.07 (0.046–0.099)

Stroke, moderate plus cognition problems	Has some difficulty in moving around, in using the hands for lifting and holding things, dressing and grooming, and in speaking. The person is often forgetful and confused.	2, 3	MoCA<24 or MMSE<26	0.316 (0.206–0.437)
Stroke, severe	Is confined to bed or a wheelchair, has difficulty speaking, and depends on others for feeding, toileting, and dressing.	4, 5	MoCA>=24 or MMSE>=26	0.552 (0.377–0.707)
Stroke, severe plus cognition problems	Is confined to bed or a wheelchair, depends on others for feeding, toileting, and dressing, and has difficulty speaking, thinking clearly, and remembering things.		MoCA<24 or MMSE<26	0.588 (0.411–0.744)

Chronic stroke severity splits

Severity level	Lay description	Modified Rankin Score	Cognitive Status	DW (95% CI)
Stroke, asymptomatic		0	N/A	N/A
Stroke, long-term consequences, mild	Has some difficulty in moving around and some weakness in one hand, but is able to walk without help.	1	N/A	0.019 (0.01–0.032)
Stroke, long-term consequences, moderate	Has some difficulty in moving around, and in using the hands for lifting and holding things, dressing, and grooming.	2, 3	MoCA>=24 or MMSE>=26	0.07 (0.046–0.099)
Stroke, long-term consequences, moderate plus cognition problems	Has some difficulty in moving around, in using the hands for lifting and holding things, dressing and grooming, and in speaking. The person is often forgetful and confused.	2, 3	MoCA<24 or MMSE<26	0.316 (0.206–0.437)

Stroke, long-term consequences, severe	Is confined to bed or a wheelchair, has difficulty speaking, and depends on others for feeding, toileting, and dressing.	4, 5	MoCA>=24 or MMSE>=26	0.552 (0.377–0.707)
Stroke, long-term consequences, severe plus cognition problems	Is confined to bed or a wheelchair, depends on others for feeding, toileting, and dressing, and has difficulty speaking, thinking clearly, and remembering things.	4, 5	MoCA<24 or MMSE<26	0.588 (0.411–0.744)

Severity split literature data availability

	Acute Proportion	Chronic Proportion
Studies	6	13
Countries/subnationals	5	11
GBD world regions	5	5

We used DisMod MR 2.1, a Bayesian meta-regression tool, to model the six severity levels, with an independent proportion model for each. Reports which grouped mRS scores differently than our mapping (e.g. 0-2) were adjusted in DisMod by estimating the association between these alternate groupings and our preferred mappings. These statistical associations were used to adjust data points to the referent category as necessary. The six models were scaled such that the sum of the proportions for all levels equaled 1.

Modelling strategy

Three general approaches were employed for all of the components of the stroke modelling process, detailed in the table below.

- Data points were adjusted from nonstandard to standard case deinitions using estimates from statistical models generated by DisMod for the acute models. Coefficients for these crosswalks can be found in the tables for fixed effects located below.
- The GBD summary exposure value, which is the relative risk-weighted prevalence of exposure, for ischaemic or haemorrhagic stroke as appropriate and a covariate for country income were used as country-level covariates for all models (4). Coefficients for these covariates can be found in the tables for fixed effects located below.
- Two versions of each stroke model were run, referred to as step 1 and step 2 models. First, we ran the step 1 DisMod-MR models for acute and chronic subtype-specific stroke using only incidence, prevalence, and case fatality data as inputs. We then used the ratio of acute:chronic cause-specific mortality estimated by these models to divide GBD stroke deaths into acute and chronic stroke deaths, using the global average for the proportion of acute:chronic stroke mortality. The acute and chronic models were then run (step 2) using the same incidence, prevalence, and case fatality data as well as the custom cause-specific mortality rates as input data.

Step 1

- We generated estimates for first-ever acute ischaemic and first-ever acute haemorrhagic stroke using DisMod-MR 2.1 with data collected on stroke incidence and excess mortality. We set value priors of 11 to 13 on remission for all ages to establish a one-month duration for these acute sequelae.
- We then calculated the rate of surviving until 28 days after an acute event for both ischaemic and haemorrhagic stroke using the modelled estimates of excess mortality and incidence.
- These survivor data were then used in the chronic ischaemic and chronic haemorrhagic stroke models as incidence inputs.
- We then ran the chronic stroke models, using the survivor incidence data and excess mortality data. Non-subtype-specific prevalence data were split into ischaemic and haemorrhagic components using the ratio of 28-day survivors from the first stage acute models. We set a value prior of 0 on remission for all ages.
- Implausible or extreme outliers in input data were dropped from these estimation results.
- From these four models, we generated the proportions of deaths due to acute ischaemic, chronic ischaemic, acute haemorrhagic, and chronic haemorrhagic stroke, and split the post-CoDCorrect stroke deaths generated from the GBD mortality estimates into these four parts, by multiplying the location-, sex-, age- and year-specific CSMR results by the global proportions estimated from the DisMod models. Thus, the mortality rates due to acute ischaemic, chronic ischaemic, acute haemorrhagic, and chronic haemorrhagic stroke are driven by all available data on incidence, prevalence, and excess mortality data for stroke. These CSMR estimates were then uploaded into the non-fatal database and used as inputs for models in Step 2.

Step 2

- We re-ran the first-ever acute ischaemic and first-ever acute haemorrhagic models with CSMR as derived from CoDCorrect and epidemiologic data as described above. Twenty-eight-day survivorship was recalculated from these models and uploaded into the chronic ischaemic and chronic haemorrhagic stroke with CSMR models. These chronic models also use CSMR as derived from CoDCorrect and epidemiologic data as described above.
- Implausible or extreme outliers were dropped from these estimation results.

Models were evaluated based on expert opinion, comparison with previous iterations, and model fit.

Changes in the modelling of stroke for GBD 2016

Several changes were made to the modelling strategy for stroke for the GBD 2016 study. In GBD 2015 and prior, chronic stroke was modelled for both subtypes (ischaemic and haemorrhagic or other) together to estimate the total prevalence of chronic stroke. For the GBD 2016 study, each stroke subtype was modelled independently, resulting in separate acute and chronic stroke models for each subtype. This change was made in order to simplify the stroke modeling process and to ensure that both subtypes were estimated correctly. In the GBD2015 and prior studies, severity splits were based on estimates derived from standard GBD analysis of the U.S. Medical Expenditure Panel Survey. For the GBD2016 study, a review of studies reporting modified Rankin scores following stroke was performed and disability weights were applied using a model of modified Rankin level by age and sex as described above.

1) Hatano S. Experience from a mulicentre stroke register: a preliminary report. Bull WHO 54, 541-553. 1976.
2) GBD 2015 Disease and Injury Incidence and Prevalence Collaborators. Global, regional, and national incidence, prevalence, and years lived with disability for 310 diseases and injuries, 1990-2015: a systematic analysis for the Global Burden of Disease Study 2015. Lancet. 2016 Oct 8;388(10053):1545-1602. doi: 10.1016/S0140-6736(16)31678-6.
3) Burstein et al. Estimating distributions of health state severity for the global burden of disease study. Population Health Metrics (2015) 13:31
4) GBD 2015 Risk Factors Collaborators. Global, regional, and national comparative risk assessment of 79 behavioural, environmental and occupational, and metabolic risks or clusters of risks, 1990-2015: a systematic analysis for the Global Burden of Disease Study 2015. Lancet. 2016 Oct 8;388(10053):1659-1724. doi: 10.1016/S0140-6736(16)31679-8.

The tables below indicate the covariates used by cause in the estimation process, as well as the beta and exponentiated beta values.

Step 1:

Cause	Variable name	Measure	beta	Exponentiated beta
Chronic ischaemic stroke	Log-transformed SEV scalar: Isch Stroke	Prevalence	0.83 (0.75 — 1.03)	2.29 (2.12 — 2.80)
Chronic ischaemic stroke	LDI (I$ per capita)	Excess mortality rate	-0.16 (-0.29 — -0.1)	0.85 (0.75 — 0.90)
Chronic haemorrhagic stroke	Log-transformed SEV scalar: Hem Stroke	Prevalence	0.79 (0.75 — 0.92)	2.21 (2.12 — 2.50)
Chronic haemorrhagic stroke	LDI (I$ per capita)	Excess mortality rate	-0.12 (-0.16 — -0.1)	0.89 (0.85 — 0.90)
First ever acute haemorrhagic stroke	Hospital data	Incidence	0.54 (0.54 – 0.54)	1.71 (1.71 – 1.72)
First ever acute haemorrhagic stroke	Any stroke	Incidence	1.27 (1.27 – 1.28)	3.57 (3.56 – 3.59)
First ever acute haemorrhagic stroke	First-ever acute stroke, ischemic or hemorrhagic	Incidence	0.52 (0.52 – 0.53)	1.69 (1.68 – 1.71)
First ever acute haemorrhagic stroke	Log-transformed age-standardized SEV scalar: hemorrhagic stroke	Incidence	0.77 (0.75 – 0.82)	2.17 (2.12 – 2.27)
First ever acute haemorrhagic stroke	Any stroke	Excess mortality rate	-0.48 (-0.66 – -0.32)	0.62 (0.52 – 0.73)
First ever acute haemorrhagic stroke	First-ever acute stroke, ischemic or hemorrhagic	Excess mortality rate	-0.081 (-0.3 – 0.16)	0.62 (0.52 – 0.73)
First ever acute ischaemic stroke	Hospital data	Incidence	0.38 (0.37 – 0.38)	1.46 (1.45 – 1.46)
First ever acute ischaemic stroke	Any stroke	Incidence	0.31 (0.29 – 0.33)	1.37 (1.34 – 1.39)
First ever acute ischaemic stroke	First-ever acute stroke, ischemic or hemorrhagic	Incidence	0.37 (0.36 – 0.38)	1.44 (1.43 – 1.46)
First ever acute ischaemic stroke	Log-transformed age-standardized SEV scalar: ischemic stroke	Incidence	1.16 (1.09 – 1.22)	3.21 (2.99 – 3.39)

Step 2:

Cause	Variable name	Measure	beta	Exponentiated beta
Chronic ischemic stroke with CSMR	Log-transformed SEV scalar: Ischaemic stroke	Prevalence	0.89 (0.75 – 1.19)	2.44 (2.13 – 3.27)
Chronic ischemic stroke with CSMR	LDI (I$ per capita)	Excess mortality rate	-0.49 (-0.5 – -0.46)	0.61 (0.61 – 0.63)
Chronic haemorrhagic stroke with CSMR	Log-transformed SEV scalar: Haemorrhagic stroke	Prevalence	0.88 (0.75 – 1.15)	2.40 (2.13 – 3.17)
Chronic haemorrhagic stroke with CSMR	LDI (I$ per capita)	Excess mortality rate	-0.48 (-0.5 – -0.44)	0.62 (0.61 – 0.64)
First-ever acute haemorrhagic stroke with CSMR	Any stroke	Incidence	1.27 (1.27 – 1.29)	3.58 (3.56 – 3.62)
First-ever acute haemorrhagic stroke with CSMR	First-ever acute stroke, ischemic or hemorrhagic	Incidence	0.52 (0.52 – 0.54)	1.69 (1.68 – 1.71)
First-ever acute haemorrhagic stroke with CSMR	Log-transformed SEV scalar: Hem stroke	Incidence	1.11 (1.01 – 1.20)	3.03 (2.74 – 3.33)
First-ever acute haemorrhagic stroke with CSMR	Any stroke	Excess mortality rate	-0.37 (-0.49 – -0.27)	0.69 (0.62 – 0.77)
First-ever acute haemorrhagic stroke with CSMR	First-ever acute stroke, ischemic or hemorrhagic	Excess mortality rate	0.023 (-0.2 – 0.23)	1.02 (0.82 – 1.25)
First-ever acute ischaemic stroke with CSMR	Any stroke	Incidence	0.32 (0.30 – 0.33)	1.38 (1.35 – 1.39)
First-ever acute ischaemic stroke with CSMR	First-ever acute stroke, ischemic or hemorrhagic	Incidence	0.37 (0.36 – 0.38)	1.44 (1.43 – 1.46)
First-ever acute ischaemic stroke with CSMR	Log-transformed age-standardized SEV scalar: Ischemic stroke	Incidence	1.11 (1.05 – 1.18)	3.04 (2.86 – 3.26)
First-ever acute ischaemic stroke with CSMR	Any stroke	Excess mortality rate	-0.34 (-0.45 – -0.24)	0.71 (0.64 – 0.79)
First-ever acute ischaemic stroke with CSMR	First-ever acute stroke, ischemic or hemorrhagic	Excess mortality rate	-0.69 (-0.82 – -0.56)	0.50 (0.44 – 0.57)

Methods Tables

Table A1: GATHER checklist of information that should be included in reports of global health estimates, with description of compliance and location of information for GBD 2016.

#	GATHER checklist item	Description of compliance	Reference
**Objectives and funding**			
1	Define the indicators, populations, and time periods for which estimates were made.	Narrative provided in paper and appendix describing indicators, definitions, and populations	Main text (Methods) and appendix
2	List the funding sources for the work.	Funding sources listed in paper	Summary (Funding)
**Data Inputs**			
For all data inputs from multiple sources that are synthesised as part of the study:			
3	Describe how the data were identified and how the data were accessed.	Narrative description of data seeking methods provided	Main text (Methods) and appendix
4	Specify the inclusion and exclusion criteria. Identify all ad-hoc exclusions.	Narrative about inclusion and exclusion criteria by data type provided; ad hoc exclusions in cause-specific write-ups	Main text (Methods) and appendix
5	Provide information on all included data sources and their main characteristics. For each data source used, report reference information or contact name/institution, population represented, data collection method, year(s) of data collection, sex and age range, diagnostic criteria or measurement method, and sample size, as relevant.	An interactive, online data source tool that provides metadata for data sources by component, geography, cause, risk, or impairment has been developed	Online data citation tools: http://ghdx.healthdata.org/gbd-2016

6	Identify and describe any categories of input data that have potentially important biases (e.g., based on characteristics listed in item 5).	Summary of known biases by cause included in appendix	Appendix

For data inputs that contribute to the analysis but were not synthesised as part of the study:

7	Describe and give sources for any other data inputs.	Included in online data source tool	http://ghdx.healthdata.org/gbd-2016

For all data inputs:

8	Provide all data inputs in a file format from which data can be efficiently extracted (e.g., a spreadsheet as opposed to a PDF), including all relevant meta-data listed in item 5. For any data inputs that cannot be shared due to ethical or legal reasons, such as third-party ownership, provide a contact name or the name of the institution that retains the right to the data.	Downloads of input data available through online tools, including data visualisation tools and data query tools; input data not available in tools will be made available upon request	Online data visualisation tools, data query tools, and the Global Health Data Exchange

Data analysis

9	Provide a conceptual overview of the data analysis method. A diagram may be helpful.	Flow diagrams of the overall methodological processes, as well as cause-specific modelling processes, have been provided	Main text (Methods) and appendix
10	Provide a detailed description of all steps of the analysis, including mathematical formulae. This description should cover, as relevant, data cleaning, data pre-processing, data adjustments and weighting of data sources, and mathematical or statistical model(s).	Flow diagrams and corresponding methodological write-ups for each cause, as well as the databases and modelling processes, have been provided	Main text (Methods) and appendix
11	Describe how candidate models were evaluated and how the final model(s) were selected.	Provided in the methodological write-ups	Appendix

12	Provide the results of an evaluation of model performance, if done, as well as the results of any relevant sensitivity analysis.	Provided in the methodological write-ups	Appendix
13	Describe methods for calculating uncertainty of the estimates. State which sources of uncertainty were, and were not, accounted for in the uncertainty analysis.	Appendix	Appendix
14	State how analytic or statistical source code used to generate estimates can be accessed.	Appendix	http://ghdx.healthdata.org/gbd-2016-code
**Results and Discussion**			
15	Provide published estimates in a file format from which data can be efficiently extracted.	GBD 2016 results are available through online data visualisation tools, the Global Health Data Exchange, and the online data query tool	Main text, and online data tools (data visualisation tools, data query tools, and the Global Health Data Exchange)
16	Report a quantitative measure of the uncertainty of the estimates (e.g. uncertainty intervals).	Uncertainty intervals are provided with all results	Main text, appendix, and online data tools (data visualisation tools, data query tools, and the Global Health Data Exchange)
17	Interpret results in light of existing evidence. If updating a previous set of estimates, describe the reasons for changes in estimates.	Discussion of methodological changes between GBD rounds provided in the narrative of the manuscript and appendix	Main text (Methods and Discussion) and appendix
18	Discuss limitations of the estimates. Include a discussion of any modelling assumptions or data limitations that affect interpretation of the estimates.	Discussion of limitations provided in the narrative of the main paper, as well as in the	Main text (Limitations) and appendix

methodological write-ups

in the appendix

Table A2: ICD Codes used in fatal and nonfatal analysis

Fatal analysis

	ICD10	ICD9
Cerebrovascular disease	G45-G46.8, I60-I63.9, I65-I66.9, I67.0-I67.3, I67.5-I67.6, I68.1-I68.2, I69.0-I69.3	430-435.9, 437.0-437.2, 437.5-437.8
Ischaemic stroke	G45-G46.8, I63-I63.9, I65-I66.9, I67.2-I67.3, I67.5-I67.6, I69.3	433-435.9, 437.0-437.1, 437.5-437.8
Hemorrhagic stroke	I60-I62.9, I67.0-I67.1, I68.1-I68.2, I69.0-I69.2	430-432.9, 437.2

Nonfatal analysis

	ICD10	ICD9
Cerebrovascular disease	I60-I63.9, I65-I66.9, I67.0-I67.3, I67.5-I67.6, I68.1-I68.2, I69.0-I69.3	430-434.9, 437.0-437.2, 437.5-437.8
Ischaemic stroke	I63-I63.9, I65-I66.9, I67.2-I67.3, I67.5-I67.6, I69.3	433-434.9, 437.0-437.1, 437.5-437.8
Hemorrhagic stroke	I60-I62.9, I67.0-I67.1, I68.1-I68.2, I69.0-I69.2	430-432.9, 437.2

Table A3: Selected covariates for CODEm models, overall stroke and subtypes

Covariate	Level	Direction, Stroke	Direction, Ischaemic stroke	Direction, Hemorrhagic stroke
Summary exposure variable	1	+	+	+
Cholesterol (total, mean per capita)	1	+	+	0
Smoking prevalence	1	+	+	+
Systolic blood pressure (mmHg)	1	+	+	+
Trans fatty acid	1	+	+	+
Mean BMI	2	+	+	+
Elevation over 1500m (proportion)	2	-	-	-
Fasting plasma glucose	2	+	+	+
Outdoor pollution ($PM_{2.5}$)	2	+	+	+
Indoor air pollution	2	+	+	+
Healthcare access and quality index	2	-	-	-
Lag distributed income per capita (I$)*	3	-	-	-
Socio-demographic Index	3	0	0	0
Omega-3 (kcal/capita, adjusted)*	3	-	-	-
Fruits (kcal/capita, adjusted)	3	-	-	-
Vegetables (kcal/capita, adjusted)	3	-	-	-
Nuts and seeds (kcal/capita, adjusted)	3	-	-	-
Whole grains (kcal/capita, adjusted)	3	-	-	-
Pulses/legumes (kcal/capita, adjusted)	3	-	-	-
PUFA adjusted (percent)	3	-	-	-
Alcohol (litres per capita)	3	0	0	0

*Variables were log-transformed

Table A4: Counts of data points used by measure and stroke model for GBD 2016

First-ever acute hemorrhagic stroke:

Region Name	Incidence	Prevalence	Remission	Mortality	Hospital Claims
East Asia	5	0	0	0	1
Southeast Asia	0	0	0	0	1
Oceania	0	0	0	0	0
Central Asia	1	0	0	1	1
Central Europe	3	0	0	1	14
Eastern Europe	5	0	0	2	5
High-income Asia Pacific	3	0	0	0	0
Australasia	9	0	0	3	4
Western Europe	29	0	0	17	31
Southern Latin America	2	0	0	2	0
High-income North America	2	0	0	0	5
Caribbean	0	0	0	0	0
Andean Latin America	0	0	0	0	0
Central Latin America	0	0	0	0	0
Tropical Latin America	0	0	0	0	0
North Africa and Middle East	8	0	0	13	2
South Asia	4	0	0	6	0
Central Sub-Saharan Africa	0	0	0	0	0
Eastern Sub-Saharan Africa	1	0	0	1	0
Southern Sub-Saharan Africa	1	0	0	0	0
Western Sub-Saharan Africa	0	0	0	0	0
**Total**	**73**	**0**	**0**	**46**	**64**

First-ever acute ischaemic stroke:

Region Name	Incidence	Prevalence	Remission	Mortality	Hospital Claims
East Asia	5	0	0	0	1
Southeast Asia	0	0	0	0	1
Oceania	0	0	0	0	0
Central Asia	1	0	0	0	1
Central Europe	3	0	0	2	14
Eastern Europe	5	0	0	1	5
High-income Asia Pacific	4	0	0	0	0
Australasia	8	0	0	0	4
Western Europe	27	0	0	6	31
Southern Latin America	2	0	0	1	0
High-income North America	1	0	0	0	5
Caribbean	0	0	0	0	0
Andean Latin America	0	0	0	0	0
Central Latin America	0	0	0	0	0
Tropical Latin America	2	0	0	0	0
North Africa and Middle East	9	0	0	7	2
South Asia	4	0	0	4	0
Central Sub-Saharan Africa	0	0	0	0	0
Eastern Sub-Saharan Africa	1	0	0	0	0
Southern Sub-Saharan Africa	1	0	0	0	0
Western Sub-Saharan Africa	0	0	0	0	0
**Total**	**73**	**0**	**0**	**21**	**64**

Chronic hemorrhagic stroke:

Region Name	Incidence	Prevalence	Remission	Mortality	Hospital Claims
East Asia	0	4	0	0	0
Southeast Asia	0	3	0	0	0
Oceania	0	0	0	0	0
Central Asia	0	0	0	0	0
Central Europe	0	0	0	1	0
Eastern Europe	0	0	0	0	0
High-income Asia Pacific	0	2	0	0	0
Australasia	0	1	0	2	0
Western Europe	0	42	0	7	0
Southern Latin America	0	1	0	0	0
High-income North America	0	39	0	0	0
Caribbean	0	2	0	0	0
Andean Latin America	0	1	0	0	0
Central Latin America	0	4	0	0	0
Tropical Latin America	0	1	0	0	0
North Africa and Middle East	0	2	0	0	0
South Asia	0	10	0	0	0
Central Sub-Saharan Africa	0	0	0	0	0
Eastern Sub-Saharan Africa	0	2	0	0	0
Southern Sub-Saharan Africa	0	0	0	0	0
Western Sub-Saharan Africa	0	4	0	0	0
**Total**	**0**	**118**	**0**	**10**	**0**

Chronic ischaemic stroke:

Region Name	Incidence	Prevalence	Remission	Mortality	Hospital Claims
East Asia	0	4	0	0	0
Southeast Asia	0	3	0	0	0
Oceania	0	0	0	0	0
Central Asia	0	0	0	0	0
Central Europe	0	0	0	1	0
Eastern Europe	0	0	0	0	0
High-income Asia Pacific	0	2	0	0	0
Australasia	0	1	0	2	0
Western Europe	0	42	0	7	0
Southern Latin America	0	1	0	0	0
High-income North America	0	39	0	0	0
Caribbean	0	2	0	0	0
Andean Latin America	0	1	0	0	0
Central Latin America	0	4	0	0	0
Tropical Latin America	0	1	0	0	0
North Africa and Middle East	0	2	0	0	0
South Asia	0	11	0	0	0
Central Sub-Saharan Africa	0	0	0	0	0
Eastern Sub-Saharan Africa	0	2	0	0	0
Southern Sub-Saharan Africa	0	0	0	0	0
Western Sub-Saharan Africa	0	4	0	0	0
**Total**	**0**	**119**	**0**	**10**	**0**

Table A5: DisMod covariates

Step 1:

Cause	Variable name	Measure	beta	Exponentiated beta
Chronic ischaemic stroke	Log-transformed SEV scalar: Isch Stroke	Prevalence	0.83 (0.75 — 1.03)	2.29 (2.12 — 2.80)
Chronic ischaemic stroke	LDI (I$ per capita)	Excess mortality rate	-0.16 (-0.29 — -0.1)	0.85 (0.75 — 0.90)
Chronic haemorrhagic stroke	Log-transformed SEV scalar: Hem Stroke	Prevalence	0.79 (0.75 — 0.92)	2.21 (2.12 — 2.50)
Chronic haemorrhagic stroke	LDI (I$ per capita)	Excess mortality rate	-0.12 (-0.16 — -0.1)	0.89 (0.85 — 0.90)
First ever acute haemorrhagic stroke	Hospital data	Incidence	0.54 (0.54 – 0.54)	1.71 (1.71 – 1.72)
First ever acute haemorrhagic stroke	Any stroke	Incidence	1.27 (1.27 – 1.28)	3.57 (3.56 – 3.59)
First ever acute haemorrhagic stroke	First-ever acute stroke, ischaemic or hemorrhagic	Incidence	0.52 (0.52 – 0.53)	1.69 (1.68 – 1.71)
First ever acute haemorrhagic stroke	Log-transformed age-standardized SEV scalar: hemorrhagic stroke	Incidence	0.77 (0.75 – 0.82)	2.17 (2.12 – 2.27)
First ever acute haemorrhagic stroke	Any stroke	Excess mortality rate	-0.48 (-0.66 – -0.32)	0.62 (0.52 – 0.73)
First ever acute haemorrhagic stroke	First-ever acute stroke, ischaemic or hemorrhagic	Excess mortality rate	-0.081 (-0.3 – 0.16)	0.62 (0.52 – 0.73)
First ever acute ischaemic stroke	Hospital data	Incidence	0.38 (0.37 – 0.38)	1.46 (1.45 – 1.46)
First ever acute ischaemic stroke	Any stroke	Incidence	0.31 (0.29 – 0.33)	1.37 (1.34 – 1.39)
First ever acute ischaemic stroke	First-ever acute stroke, ischaemic or hemorrhagic	Incidence	0.37 (0.36 – 0.38)	1.44 (1.43 – 1.46)
First ever acute ischaemic stroke	Log-transformed age-standardized SEV scalar: ischaemic stroke	Incidence	1.16 (1.09 – 1.22)	3.21 (2.99 – 3.39)

Step 2:

Cause	Variable name	Measure	beta	Exponentiated beta
Chronic ischaemic stroke with CSMR	Log-transformed SEV scalar: Ischaemic stroke	Prevalence	0.89 (0.75 – 1.19)	2.44 (2.13 – 3.27)
Chronic ischaemic stroke with CSMR	LDI (I$ per capita)	Excess mortality rate	-0.49 (-0.5 – -0.46)	0.61 (0.61 – 0.63)
Chronic haemorrhagic stroke with CSMR	Log-transformed SEV scalar: Haemorrhagic stroke	Prevalence	0.88 (0.75 – 1.15)	2.40 (2.13 – 3.17)
Chronic haemorrhagic stroke with CSMR	LDI (I$ per capita)	Excess mortality rate	-0.48 (-0.5 – -0.44)	0.62 (0.61 – 0.64)
First-ever acute haemorrhagic stroke with CSMR	Any stroke	Incidence	1.27 (1.27 – 1.29)	3.58 (3.56 – 3.62)
First-ever acute haemorrhagic stroke with CSMR	First-ever acute stroke, ischaemic or hemorrhagic	Incidence	0.52 (0.52 – 0.54)	1.69 (1.68 – 1.71)
First-ever acute haemorrhagic stroke with CSMR	Log-transformed SEV scalar: Hem stroke	Incidence	1.11 (1.01 – 1.20)	3.03 (2.74 – 3.33)
First-ever acute haemorrhagic stroke with CSMR	Any stroke	Excess mortality rate	-0.37 (-0.49 – -0.27)	0.69 (0.62 – 0.77)
First-ever acute haemorrhagic stroke with CSMR	First-ever acute stroke, ischaemic or hemorrhagic	Excess mortality rate	0.023 (-0.2 – 0.23)	1.02 (0.82 – 1.25)
First-ever acute ischaemic stroke with CSMR	Any stroke	Incidence	0.32 (0.30 – 0.33)	1.38 (1.35 – 1.39)
First-ever acute ischaemic stroke with CSMR	First-ever acute stroke, ischaemic or hemorrhagic	Incidence	0.37 (0.36 – 0.38)	1.44 (1.43 – 1.46)
First-ever acute ischaemic stroke with CSMR	Log-transformed age-standardized SEV scalar: Ischaemic stroke	Incidence	1.11 (1.05 – 1.18)	3.04 (2.86 – 3.26)
First-ever acute ischaemic stroke with CSMR	Any stroke	Excess mortality rate	-0.34 (-0.45 – -0.24)	0.71 (0.64 – 0.79)
First-ever acute ischaemic stroke with CSMR	First-ever acute stroke, ischaemic or hemorrhagic	Excess mortality rate	-0.69 (-0.82 – -0.56)	0.51 (0.44 – 0.57)

Table A6: Sequelae and disability weights for ischaemic and hemorrhagic stroke

Sequela	Health state lay description	Disability weight
Asymptomatic chronic stroke		N/A
Acute and chronic stroke, severity level 1	Has some difficulty in moving around and some weakness in one hand, but is able to walk without help	0.019 (0.01 – 0.032)
Acute and chronic stroke, severity level 2	Has some difficulty in moving around and in using the hands for lifting and holding things, dressing and grooming	0.07 (0.046 – 0.099)
Acute and chronic stroke, severity level 3	Has some difficulty in moving around, in using the hands for lifting and holding things, dressing and grooming, and in speaking. The person is often forgetful and confused.	0.316 (0.205 – 0.438)
Acute and chronic stroke, severity level 4	Is confined to bed or a wheelchair, has difficulty speaking and depends on others for feeding, toileting and dressing.	0.552 (0.376 – 0.707)
Acute and chronic stroke, severity level 5	Is confined to bed or a wheelchair, depends on others for feeding, toileting and dressing, and has difficulty speaking, thinking clearly and remembering things.	0.588 (0.411 – 0.745)

Supplementary Tables and Figures

Figure A1: DALYs attributable to risk factors; all ages by sex and stroke subtype for 1990 and 2016; risk factors are ranked by percent attributable fraction shown.

Global ischaemic stroke DALYs attributable to risk factors; all ages by sex for 1990 and 2016; risk factors are ranked by percent attributable fraction shown.

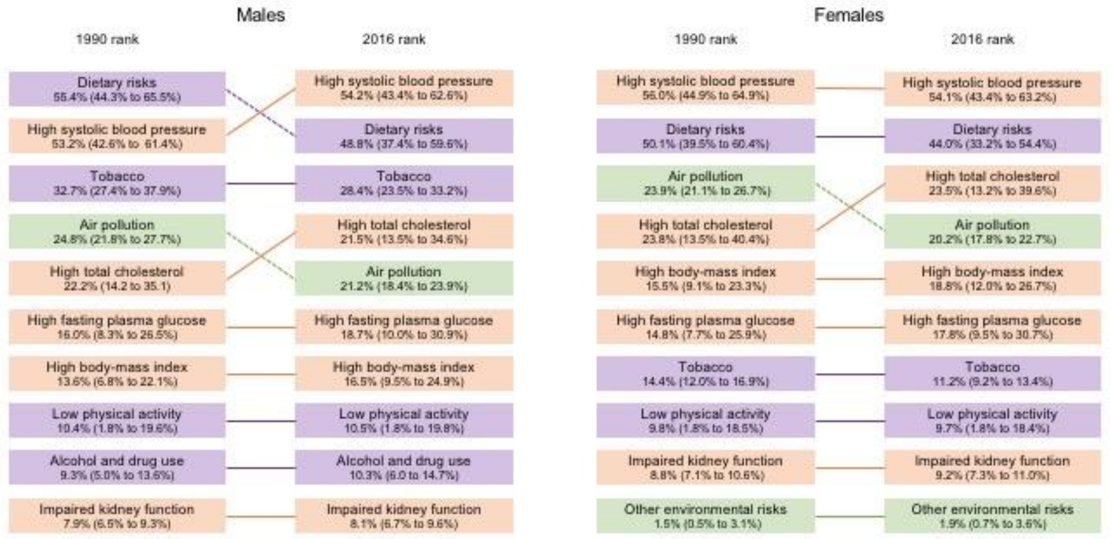

Global haemorrhagic stroke DALYs attributable to risk factors; all ages by sex for 1990 and 2016; risk factors are ranked by percent attributable fraction shown.

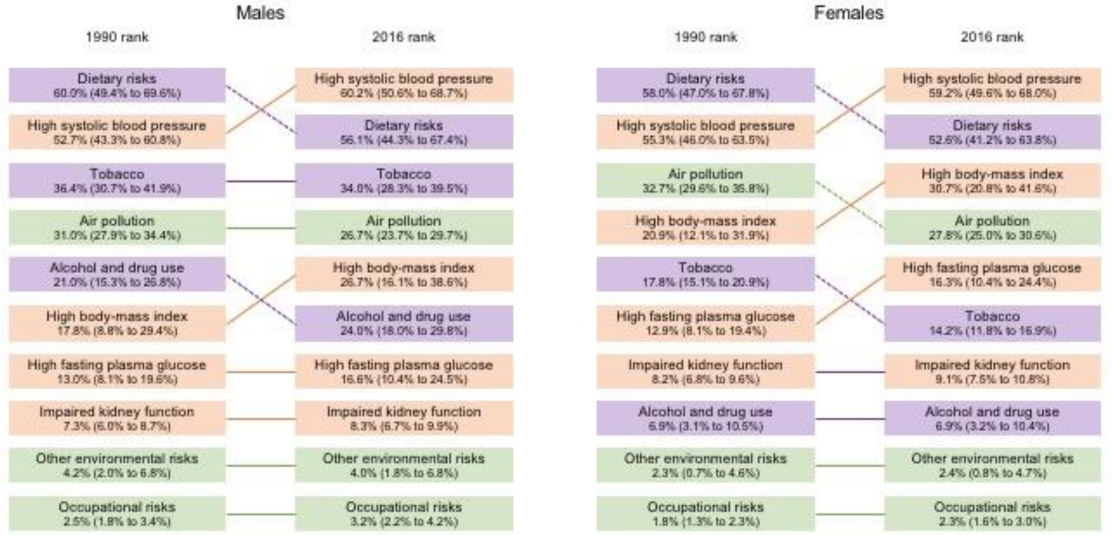

Table A7: Incidence, deaths and DALYs for 2016 and percentage change of age-standardized rates by location for Ischemic stroke

Location	Deaths (95% UI)		Incidence (95% UI)		DALYs (95% UI)	
	2016 counts	Percentage change in age-standardised rates between 1990 and 2016	2016 counts	Percentage change in age-standardised rates between 1990 and 2016	2016 counts	Percentage change in age-standardised rates between 1990 and 2016
**Global**	**2 690 170 (2 571 770 to 2 817 623)**	**-34.5 (-37.1 to -31.8)**	**9 556 443 (8 654 630 to 10 512 905)**	**-5.4 (-6.6 to -4.2)**	**51 897 437 (47 896 550 to 55 567 646)**	**-29.2 (-32.0 to -26.2)**
**High SDI**	**429 663 (399 216 to 460 964)**	**-54.6 (-56.1 to -53.0)**	**1 791 320 (1 621 796 to 1 959 587)**	**-20.3 (-21.4 to -19.1)**	**6 532 239 (5 791 718 to 7 205 027)**	**-48.9 (-51.6 to -46.2)**
**High-middle SDI**	**663 976 (599 555 to 739 854)**	**-42.9 (-48.3 to -36.4)**	**2 428 051 (2 195 540 to 2 667 827)**	**-14.3 (-15.8 to -12.9)**	**12 192 246 (10 824 134 to 13 610 902)**	**-38.1 (-43.7 to -32.1)**
**Middle SDI**	**924 336 (887 183 to 962 150)**	**-27.4 (-32.8 to -22.5)**	**3 627 545 (3 255 281 to 3 987 965)**	**9.8 (8.5 to 11.1)**	**19 247 043 (17 862 440 to 20 661 197)**	**-23.3 (-28.1 to -18.6)**
**Low-middle SDI**	**550 697 (516 112 to 587 951)**	**-15.3 (-21.6 to -7.8)**	**1 391 031 (1 246 456 to 1 542 034)**	**3.3 (2.2 to 4.4)**	**11 311 804 (10 554 602 to 12 188 078)**	**-15.8 (-21.4 to -9.1)**
**Low SDI**	**120 112 (110 946 to 129 326)**	**-17.5 (-23.3 to -9.5)**	**291 575 (258 421 to 325 203)**	**-2.3 (-3.5 to -1.1)**	**2 562 776 (2 375 815 to 2 756 203)**	**-17.8 (-23.1 to -10.7)**
**High-income North America**	**125 923 (118 280 to 134 086)**	**-25.6 (-28.6 to -23.0)**	**602 049 (546 325 to 658 957)**	**-16.7 (-17.8 to -15.7)**	**2 042 705 (1 806 894 to 2 264 918)**	**-23.9 (-26.9 to -21.0)**
Canada	12 632 (11 444 to 14 051)	-41.4 (-46.6 to -35.9)	60 288 (53 885 to 67 257)	-19.4 (-21.4 to -17.6)	185 508 (158 484 to 210 038)	-37.0 (-41.9 to -32.3)
Greenland	12 (10 to 16)	-57.7 (-64.9 to -49.2)	58 (50 to 66)	-31.7 (-33.4 to -30.0)	275 (224 to 332)	-53.0 (-60.6 to -44.6)
USA	113 279 (106 424 to 120 430)	-23.8 (-27.0 to -20.9)	541 511 (491 932 to 591 924)	-16.4 (-17.4 to -15.3)	1 856 673 (1 643 792 to 2 055 796)	-22.4 (-25.5 to -19.4)

**Australasia**	**7 833 (7 090 to 8 546)**	**-52.7 (-57.0 to -48.1)**	**31 498 (28 525 to 34 695)**	**-23.7 (-25.3 to -22.1)**	**102 555 (89 408 to 114 758)**	**-50.9 (-55.0 to -47.1)**
Australia	6 574 (5 871 to 7 254)	-54.1 (-58.6 to -49.1)	24 682 (22 030 to 27 527)	-28.9 (-30.8 to -27.2)	84 977 (73 947 to 94 953)	-51.9 (-56.3 to -47.9)
New Zealand	1 259 (1 097 to 1 430)	-45.2 (-51.5 to -38.3)	6 816 (6 072 to 7 331)	3.5 (0.4 to 7.0)	17 578 (15 228 to 20 101)	-45.7 (-50.9 to -40.2)
**High-income Asia-Pacific**	**97 510 (88 640 to 106 191)**	**-70.2 (-72.3 to -67.8)**	**311 170 (277 815 to 345 544)**	**-33.4 (-34.7 to -32.2)**	**1 378 765 (1 215 582 to 1 530 286)**	**-63.1 (-66.3 to -59.9)**
Brunei	53 (46 to 59)	-37.3 (-46.0 to -28.0)	253 (223 to 284)	-24.4 (-26.0 to -22.6)	1 208 (1 041 to 1 377)	-36.6 (-44.6 to -28.8)
Japan	74 503 (68 306 to 80 636)	-70.4 (-72.0 to -69.0)	245 001 (218 576 to 271 353)	-29.8 (-31.1 to -28.5)	1 000 405 (883 701 to 1 108 850)	-60.9 (-63.8 to -58.3)
Singapore	617 (504 to 746)	-76.4 (-80.9 to -70.8)	4 180 (3 699 to 4 699)	-37.1 (-38.9 to -35.4)	14 272 (11 800 to 16 695)	-66.9 (-72.1 to -61.1)
South Korea	22 337 (17 358 to 27 791)	-72.5 (-79.2 to -64.1)	61 736 (54 297 to 69 236)	-47.5 (-49.2 to -46.0)	362 881 (295 633 to 436 337)	-71.9 (-77.4 to -65.5)
**Western Europe**	**167 818 (152 667 to 185 524)**	**-62.6 (-64.6 to -60.4)**	**708 407 (640 914 to 777 120)**	**-22.7 (-24.1 to -21.3)**	**2 376 025 (2 094 053 to 2 643 900)**	**-56.9 (-59.8 to -54.2)**
Andorra	22 (18 to 28)	-48.0 (-59.5 to -33.4)	123 (109 to 139)	-16.9 (-18.7 to -15.4)	352 (287 to 422)	-35.4 (-45.4 to -25.1)
Austria	2 176 (1 859 to 2 551)	-78.7 (-80.9 to -76.1)	16 575 (14 924 to 18 374)	-25.3 (-27.3 to -23.3)	40 550 (33 902 to 47 281)	-68.1 (-71.9 to -64.2)
Belgium	3 889 (3 324 to 4 460)	-63.0 (-67.5 to -58.5)	19 667 (17 327 to 22 010)	-15.0 (-18.0 to -12.3)	60 551 (51 949 to 69 278)	-54.2 (-59.1 to -49.4)
Cyprus	252 (219 to 289)	-60.6 (-66.7 to -53.4)	937 (836 to 1 048)	-30.7 (-32.1 to -29.2)	3 772 (3 269 to 4 278)	-54.2 (-60.6 to -47.7)
Denmark	2 061 (1 805 to 2 345)	-46.0 (-53.3 to -38.2)	8 391 (7 457 to 9 367)	-23.8 (-25.7 to -22.0)	31 473 (27 631 to 35 389)	-44.3 (-50.2 to -38.4)
Finland	3 670 (3 133 to 4 273)	-54.1 (-59.6 to -48.0)	12 717 (11 315 to 14 133)	-20.4 (-22.7 to -18.1)	49 669 (43 163 to 56 448)	-52.2 (-56.8 to -47.3)

France	20 422 (17 986 to 23 381)	-57.9 (-61.7 to -53.7)	89 046 (79 502 to 98 908)	-20.1 (-22.1 to -18.1)	294 645 (255 187 to 331 694)	-47.6 (-52.2 to -43.3)
Germany	34 734 (30 134 to 39 765)	-64.0 (-68.1 to -59.1)	175 722 (155 872 to 196 369)	-11.3 (-14.4 to -8.3)	575 150 (496 415 to 650 334)	-55.1 (-59.4 to -50.1)
Greece	8 486 (7 482 to 9 577)	-57.1 (-61.5 to -52.5)	22 182 (19 742 to 24 707)	-26.7 (-28.7 to -24.7)	103 609 (92 021 to 115 395)	-53.2 (-57.3 to -49.1)
Iceland	87 (75 to 99)	-44.8 (-51.1 to -37.5)	431 (384 to 481)	-13.0 (-15.2 to -10.8)	1 297 (1 114 to 1 478)	-42.6 (-48.2 to -37.1)
Ireland	970 (833 to 1 112)	-65.0 (-69.6 to -59.8)	4 927 (4 377 to 5 508)	-31.9 (-33.9 to -29.9)	16 037 (13 675 to 18 400)	-58.9 (-63.7 to -54.0)
Israel	1 368 (1 151 to 1 621)	-68.0 (-73.6 to -61.6)	7 648 (6 755 to 8 551)	-31.7 (-33.5 to -29.8)	23 460 (19 722 to 27 364)	-61.1 (-66.6 to -54.8)
Italy	27 178 (23 257 to 31 879)	-65.3 (-69.0 to -60.7)	110 917 (103 644 to 117 073)	-26.2 (-28.0 to -24.6)	325 996 (281 826 to 368 135)	-63.8 (-67.3 to -59.9)
Luxembourg	163 (141 to 184)	-73.2 (-76.5 to -69.9)	723 (662 to 782)	-35.4 (-37.1 to -33.7)	2 531 (2 185 to 2 850)	-67.0 (-70.6 to -63.3)
Malta	128 (108 to 150)	-62.3 (-68.6 to -54.8)	550 (485 to 617)	-34.9 (-36.6 to -33.3)	2 140 (1 821 to 2 478)	-58.1 (-63.8 to -52.3)
Netherlands	5 859 (5 165 to 6 584)	-42.6 (-48.8 to -35.1)	23 881 (21 142 to 26 763)	-15.0 (-17.6 to -12.6)	87 451 (76 666 to 98 495)	-39.3 (-44.4 to -33.9)
Norway	1 554 (1 332 to 1 788)	-60.4 (-65.7 to -54.9)	8 377 (7 467 to 9 354)	-18.1 (-20.4 to -16.0)	23 913 (20 454 to 27 238)	-52.1 (-57.2 to -47.0)
Portugal	7 360 (6 648 to 8 147)	-73.7 (-76.0 to -71.2)	18 103 (16 315 to 20 104)	-51.0 (-52.3 to -49.8)	93 996 (84 140 to 103 548)	-71.8 (-74.2 to -69.2)
Spain	15 086 (13 154 to 17 331)	-72.2 (-75.2 to -69.0)	67 713 (60 096 to 75 653)	-33.4 (-35.2 to -31.6)	193 480 (167 212 to 218 806)	-66.8 (-70.3 to -63.6)
Sweden	4 524 (3 900 to 5 240)	-41.5 (-48.9 to -33.6)	17 353 (15 347 to 19 407)	-11.0 (-13.1 to -9.0)	60 304 (52 145 to 68 516)	-40.7 (-46.2 to -35.0)
Switzerland	2 486 (1 934 to 3 179)	-62.1 (-70.1 to -53.0)	13 241 (11 766 to 14 682)	-11.2 (-14.3 to -8.8)	37 406 (30 450 to 44 691)	-51.6 (-58.7 to -44.3)

United Kingdom	25 342 (23 509 to 27 178)	-57.1 (-58.7 to -55.5)	88 538 (79 371 to 98 324)	-28.4 (-29.7 to -27.2)	347 475 (309 704 to 382 348)	-53.6 (-56.4 to -51.2)
**Southern Latin America**	**19 157 (17 332 to 20 768)**	**-61.9 (-65.1 to -58.3)**	**66 113 (59 452 to 73 265)**	**-38.0 (-39.4 to -36.6)**	**314 511 (281 805 to 345 187)**	**-63.7 (-66.8 to -60.6)**
Argentina	11 456 (10 337 to 12 476)	-64.1 (-67.5 to -60.6)	41 210 (37 027 to 45 843)	-40.1 (-41.7 to -38.5)	195 478 (175 735 to 216 332)	-65.5 (-68.6 to -62.4)
Chile	5 481 (4 433 to 6 700)	-59.8 (-68.0 to -50.6)	19 687 (17 471 to 22 052)	-33.7 (-35.5 to -32.1)	88 525 (73 811 to 105 263)	-62.0 (-68.3 to -54.9)
Uruguay	2 220 (2 033 to 2 425)	-51.3 (-55.2 to -46.9)	5 211 (4 658 to 5 784)	-32.8 (-34.4 to -31.3)	30 502 (27 822 to 33 045)	-52.4 (-55.8 to -48.5)
**Eastern Europe**	**342 169 (281 418 to 415 791)**	**-30.0 (-42.2 to -14.8)**	**773 145 (678 501 to 865 274)**	**-15.2 (-17.5 to -13.1)**	**5 695 149 (4 784 886 to 6 821 695)**	**-27.4 (-39.0 to -13.9)**
Belarus	10 349 (8 751 to 11 971)	-24.9 (-36.3 to -13.0)	30 112 (26 751 to 33 673)	-13.0 (-15.3 to -10.7)	188 243 (161 934 to 213 457)	-24.7 (-35.2 to -14.6)
Estonia	928 (756 to 1 144)	-75.8 (-80.1 to -69.7)	3 745 (3 303 to 4 212)	-38.1 (-39.9 to -36.4)	17 403 (14 509 to 20 627)	-68.9 (-73.3 to -63.3)
Latvia	3 626 (3 171 to 4 093)	-39.9 (-47.3 to -31.1)	10 108 (8 794 to 11 387)	-11.6 (-14.7 to -8.9)	56 188 (49 639 to 63 164)	-36.6 (-43.5 to -28.2)
Lithuania	3 519 (3 174 to 3 872)	-17.2 (-25.1 to -8.4)	12 538 (11 248 to 13 768)	-1.1 (-3.3 to 1.1)	57 979 (51 961 to 63 873)	-21.1 (-27.8 to -13.8)
Moldova	3 275 (2 898 to 3 666)	-32.2 (-40.8 to -22.2)	9 707 (8 642 to 10 708)	-13.0 (-15.0 to -11.1)	66 066 (58 926 to 73 483)	-24.9 (-33.1 to -15.4)
Russia	258 005 (199 821 to 330 603)	-28.1 (-44.6 to -7.1)	543 078 (475 191 to 611 918)	-14.8 (-17.5 to -12.4)	4 228 949 (3 338 120 to 5 297 810)	-26.1 (-41.7 to -7.0)
Ukraine	62 468 (50 732 to 76 775)	-38.6 (-50.1 to -24.5)	163 857 (144 204 to 183 357)	-18.5 (-21.0 to -16.0)	1 080 320 (901 746 to 1 294 684)	-32.6 (-43.5 to -18.9)
**Central Europe**	**108 844 (101 306 to 118 239)**	**-43.0 (-46.0 to -39.7)**	**370 504 (335 727 to 403 092)**	**-10.4 (-12.1 to -8.6)**	**1 760 878 (1 609 966 to 1 909 459)**	**-40.9 (-43.9 to -37.7)**

Albania	731 (626 to 834)	-19.4 (-31.2 to -5.5)	5 040 (4 466 to 5 642)	14.8 (12.0 to 17.3)	15 582 (13 332 to 17 948)	-10.2 (-19.8 to 0.6)
Bosnia and Herzegovina	5 040 (4 373 to 5 818)	-12.9 (-27.0 to 3.0)	13 837 (12 195 to 15 343)	11.9 (9.2 to 14.7)	84 435 (74 433 to 96 153)	-5.6 (-19.1 to 9.5)
Bulgaria	12 220 (10 725 to 13 951)	-20.4 (-30.8 to -8.5)	29 108 (25 784 to 32 543)	-5.4 (-8.2 to -2.7)	189 600 (166 092 to 213 764)	-20.3 (-29.7 to -10.1)
Croatia	4 424 (3 851 to 5 037)	-39.1 (-47.3 to -30.0)	16 638 (15 367 to 17 697)	0.5 (-2.6 to 3.3)	67 867 (59 953 to 76 415)	-39.2 (-46.2 to -31.7)
Czech Republic	7 011 (6 398 to 7 658)	-69.7 (-72.3 to -66.6)	31 094 (27 572 to 34 882)	-28.6 (-31.0 to -26.6)	112 283 (99 229 to 125 964)	-66.0 (-69.2 to -62.5)
Hungary	9 606 (8 475 to 10 723)	-52.3 (-57.8 to -46.5)	32 663 (29 019 to 36 399)	-24.8 (-26.8 to -23.0)	162 489 (144 106 to 180 514)	-50.0 (-55.2 to -44.9)
Macedonia	2 574 (2 247 to 3 188)	-21.1 (-29.0 to -12.6)	6 020 (5 273 to 6 728)	-13.5 (-15.7 to -11.3)	42 298 (37 778 to 49 239)	-23.9 (-30.5 to -16.8)
Montenegro	232 (199 to 264)	7.2 (-11.2 to 28.2)	1 375 (1 210 to 1 550)	15.1 (12.4 to 17.6)	4 388 (3 752 to 5 010)	3.6 (-8.6 to 16.2)
Poland	23 872 (21 201 to 26 806)	-50.1 (-55.5 to -44.0)	102 009 (91 292 to 110 477)	-2.1 (-5.3 to 0.8)	403 913 (354 873 to 448 851)	-46.6 (-51.7 to -41.2)
Romania	26 801 (24 184 to 29 755)	-32.1 (-39.0 to -24.4)	79 643 (70 183 to 89 413)	-7.9 (-11.1 to -4.8)	409 143 (366 655 to 453 970)	-32.5 (-38.5 to -25.9)
Serbia	11 865 (10 300 to 14 724)	-25.0 (-34.2 to -14.4)	31 889 (29 971 to 33 563)	-8.6 (-11.3 to -6.3)	189 537 (167 871 to 221 840)	-25.4 (-32.9 to -16.3)
Slovakia	3 378 (2 972 to 3 793)	-43.3 (-51.1 to -34.8)	16 484 (14 589 to 18 488)	0.3 (-3.1 to 3.3)	62 069 (54 501 to 69 635)	-40.0 (-47.0 to -32.8)
Slovenia	1 091 (918 to 1 295)	-72.6 (-76.6 to -68.3)	4 704 (4 317 to 5 069)	-36.4 (-37.9 to -35.0)	17 274 (14 746 to 19 836)	-67.8 (-72.3 to -63.5)
**Central Asia**	**30 311 (28 085 to 32 853)**	**-31.2 (-35.9 to -25.5)**	**89 097 (79 841 to 98 278)**	**-12.1 (-13.6 to -10.6)**	**633 970 (576 727 to 690 893)**	**-27.7 (-32.3 to -22.4)**
Armenia	1 433 (1 273 to 1 606)	-49.2 (-55.5 to -42.5)	4 713 (4 159 to 5 247)	-20.2 (-22.3 to -18.1)	26 383 (23 319 to 29 315)	-43.8 (-49.9 to -38.0)

Azerbaijan	2 397<br>(1 966 to 2 849)	-18.9<br>(-33.1 to -2.0)	10 270<br>(8 998 to 11 591)	9.0<br>(6.2 to 11.4)	53 918<br>(45 818 to 63 455)	-15.6<br>(-28.8 to -1.5)
Georgia	3 596<br>(3 107 to 4 139)	-26.6<br>(-38.5 to -13.3)	9 095<br>(8 351 to 9 647)	-1.5<br>(-4.4 to 1.2)	58 989<br>(51 191 to 67 220)	-25.6<br>(-36.8 to -13.7)
Kazakhstan	10 420<br>(8 946 to 12 287)	-23.8<br>(-35.0 to -9.8)	24 807<br>(22 087 to 27 627)	-12.6<br>(-15.1 to -10.5)	213 439<br>(183 900 to 247 946)	-21.0<br>(-31.3 to -7.7)
Kyrgyzstan	2 270<br>(2 062 to 2 496)	-36.6<br>(-43.6 to -29.3)	5 240<br>(4 663 to 5 814)	-21.9<br>(-24.0 to -20.1)	47 974<br>(43 469 to 52 634)	-28.9<br>(-36.3 to -21.3)
Mongolia	312<br>(272 to 355)	6.8<br>(-10.4 to 27.0)	1 999<br>(1 764 to 2 244)	20.1<br>(17.6 to 22.5)	9 239<br>(8 037 to 10 597)	12.1<br>(-3.1 to 28.9)
Tajikistan	2 160<br>(1 872 to 2 515)	-1.4<br>(-14.5 to 16.1)	5 471<br>(4 865 to 6 105)	-5.6<br>(-7.7 to -3.4)	42 379<br>(36 834 to 49 177)	-4.0<br>(-16.4 to 11.4)
Turkmenistan	276<br>(246 to 309)	-27.1<br>(-36.2 to -17.5)	2 970<br>(2 586 to 3 372)	17.5<br>(15.3 to 19.9)	7 690<br>(6 461 to 8 894)	-16.4<br>(-23.8 to -8.0)
Uzbekistan	7 447<br>(6 466 to 8 651)	-34.7<br>(-43.3 to -25.9)	24 533<br>(21 708 to 27 433)	-15.9<br>(-17.9 to -13.8)	173 960<br>(151 944 to 201 102)	-32.6<br>(-40.3 to -24.3)
**Central Latin America**	**24 243<br>(22 222 to 26 076)**	**-52.5<br>(-55.1 to -49.9)**	**140 439<br>(124 060 to 157 744)**	**-11.4<br>(-13.0 to -10.0)**	**454 046<br>(416 443 to 491 199)**	**-49.8<br>(-52.6 to -46.9)**
Colombia	4 280<br>(3 700 to 4 824)	-61.4<br>(-66.5 to -56.4)	28 029<br>(24 675 to 31 831)	-20.1<br>(-22.2 to -18.1)	78 795<br>(69 054 to 88 305)	-60.8<br>(-65.3 to -56.2)
Costa Rica	434<br>(379 to 503)	-64.5<br>(-68.4 to -59.9)	3 267<br>(2 865 to 3 702)	-15.1<br>(-17.4 to -12.9)	7 939<br>(6 955 to 8 972)	-58.7<br>(-62.6 to -54.3)
El Salvador	574<br>(495 to 657)	-73.6<br>(-77.2 to -69.9)	3 513<br>(3 087 to 3 960)	-22.9<br>(-25.0 to -21.0)	10 829<br>(9 437 to 12 198)	-71.8<br>(-75.4 to -68.2)
Guatemala	1 447<br>(1 159 to 1 772)	-33.0<br>(-47.3 to -16.7)	6 659<br>(5 830 to 7 471)	-3.6<br>(-6.1 to -1.3)	27 778<br>(22 730 to 33 543)	-35.6<br>(-48.0 to -21.5)
Honduras	1 354<br>(1 048 to 1 708)	-35.6<br>(-50.0 to -16.4)	4 280<br>(3 801 to 4 816)	-4.0<br>(-6.3 to -1.7)	25 818<br>(20 298 to 32 952)	-37.3<br>(-51.1 to -18.4)
Mexico	11 374<br>(10 389 to 12 239)	-46.3<br>(-49.1 to -43.6)	70 957<br>(62 726 to 79 602)	-4.8<br>(-6.4 to -3.3)	217 213<br>(198 579 to 234 652)	-40.6<br>(-43.3 to -37.6)

Nicaragua	541 (458 to 639)	-52.1 (-59.8 to -43.6)	3 096 (2 730 to 3 479)	-12.0 (-14.2 to -9.8)	9 764 (8 356 to 11 230)	-49.6 (-56.6 to -42.2)
Panama	653 (572 to 740)	-59.0 (-64.6 to -52.5)	2 637 (2 342 to 2 948)	-25.8 (-27.7 to -23.9)	10 197 (8 989 to 11 520)	-57.7 (-63.2 to -51.5)
Venezuela	3 586 (3 046 to 4 212)	-57.4 (-63.8 to -49.3)	18 000 (15 897 to 20 300)	-20.5 (-22.6 to -18.4)	65 713 (56 355 to 76 354)	-57.2 (-63.3 to -50.0)
**Andean Latin America**	**6 582 (5 878 to 7 381)**	**-60.6 (-65.2 to -55.8)**	**34 603 (30 687 to 38 740)**	**-19.6 (-21.3 to -18.0)**	**124 819 (110 930 to 139 469)**	**-59.5 (-63.8 to -54.7)**
Bolivia	1 935 (1 544 to 2 392)	-52.4 (-61.7 to -41.0)	7 049 (6 225 to 7 916)	-15.6 (-17.7 to -13.6)	34 764 (27 918 to 42 885)	-53.3 (-62.1 to -42.8)
Ecuador	1 856 (1 664 to 2 062)	-57.5 (-61.9 to -52.4)	9 134 (8 061 to 10 185)	-22.2 (-24.3 to -20.1)	35 627 (31 925 to 39 304)	-58.6 (-62.7 to -54.6)
Peru	2 790 (2 318 to 3 347)	-66.5 (-73.1 to -58.7)	18 420 (16 341 to 20 776)	-19.8 (-22.0 to -17.9)	54 429 (45 708 to 64 017)	-62.9 (-69.2 to -55.4)
**Caribbean**	**16 393 (15 129 to 17 636)**	**-34.6 (-39.1 to -29.8)**	**42 716 (38 061 to 47 358)**	**-15.2 (-16.7 to -13.8)**	**273 637 (250 807 to 297 540)**	**-38.7 (-43.4 to -33.8)**
Antigua and Barbuda	19 (16 to 21)	-61.7 (-66.9 to -55.4)	71 (63 to 80)	-28.0 (-29.7 to -26.3)	345 (302 to 386)	-59.3 (-64.4 to -53.4)
The Bahamas	106 (90 to 122)	-40.2 (-48.3 to -31.6)	339 (300 to 380)	-18.1 (-20.1 to -16.2)	1 943 (1 689 to 2 180)	-41.6 (-47.9 to -34.0)
Barbados	132 (118 to 147)	-55.5 (-60.5 to -50.1)	370 (328 to 413)	-26.2 (-28.0 to -24.2)	1 976 (1 774 to 2 187)	-52.8 (-57.8 to -47.5)
Belize	50 (43 to 57)	-19.1 (-30.9 to -6.0)	168 (148 to 189)	-6.8 (-8.7 to -4.7)	969 (843 to 1 104)	-22.8 (-34.1 to -11.2)
Bermuda	15 (13 to 18)	-66.4 (-71.4 to -60.7)	56 (49 to 62)	-35.3 (-37.1 to -33.7)	245 (213 to 279)	-65.9 (-70.4 to -60.9)
Cuba	5 826 (5 257 to 6 440)	-21.3 (-29.1 to -12.9)	15 133 (13 457 to 16 798)	-12.2 (-14.3 to -10.2)	85 352 (77 327 to 93 828)	-28.0 (-34.5 to -21.0)
Dominica	24 (20 to 28)	-41.3 (-49.8 to -30.1)	64 (57 to 71)	-15.9 (-18.0 to -14.0)	375 (326 to 427)	-39.9 (-47.9 to -29.9)
Dominican Republic	2 490 (2 101 to 2 856)	-42.4 (-51.8 to -32.0)	7 552 (6 677 to 8 477)	-16.2 (-18.4 to -14.1)	41 018 (34 880 to 46 622)	-44.7 (-53.3 to -35.7)

Grenada	44 (38 to 49)	-43.7 (-51.9 to -35.2)	92 (82 to 102)	-22.3 (-24.1 to -20.6)	699 (614 to 792)	-44.0 (-52.1 to -35.6)
Guyana	264 (232 to 295)	-47.3 (-54.4 to -40.5)	587 (517 to 654)	-27.1 (-28.9 to -25.3)	5 660 (4 973 to 6 336)	-51.9 (-58.1 to -45.7)
Haiti	4 302 (3 427 to 5 240)	-37.5 (-48.8 to -24.7)	7 008 (6 195 to 7 817)	-21.9 (-23.4 to -20.2)	85 163 (67 786 to 104 977)	-42.1 (-53.0 to -29.8)
Jamaica	1 460 (1 249 to 1 681)	-30.8 (-42.5 to -16.7)	2 964 (2 638 to 3 305)	-16.7 (-18.6 to -14.7)	20 504 (17 613 to 23 580)	-34.9 (-45.6 to -23.0)
Puerto Rico	876 (771 to 988)	-38.5 (-45.9 to -30.9)	3 970 (3 501 to 4 439)	-1.7 (-4.0 to 0.4)	13 514 (11 876 to 15 018)	-36.7 (-43.1 to -30.3)
Saint Lucia	55 (49 to 60)	-58.4 (-63.0 to -54.1)	163 (145 to 181)	-28.8 (-30.5 to -27.1)	935 (842 to 1 022)	-56.3 (-60.7 to -52.1)
Saint Vincent and the Grenadines	41 (36 to 45)	-31.0 (-39.1 to -22.2)	96 (86 to 107)	-13.6 (-15.5 to -11.8)	692 (621 to 763)	-31.3 (-39.1 to -23.5)
Suriname	189 (170 to 208)	-21.0 (-29.7 to -11.8)	481 (428 to 532)	-10.5 (-12.6 to -8.6)	3 410 (3 090 to 3 752)	-25.2 (-33.0 to -17.4)
Trinidad and Tobago	458 (415 to 506)	-53.1 (-57.9 to -48.0)	1 399 (1 239 to 1 562)	-29.3 (-30.9 to -27.8)	8 377 (7 610 to 9 220)	-53.8 (-58.3 to -49.1)
Virgin Islands	42 (37 to 48)	-30.9 (-41.0 to -18.7)	151 (133 to 170)	-5.7 (-7.9 to -3.7)	717 (625 to 808)	-31.3 (-40.4 to -21.0)
**Tropical Latin America**	**67 260 (63 201 to 70 906)**	**-58.2 (-59.9 to -56.5)**	**188 137 (168 347 to 208 970)**	**-32.0 (-33.2 to -30.9)**	**1 137 874 (1 068 956 to 1 201 360)**	**-58.7 (-60.5 to -57.0)**
Brazil	66 270 (62 224 to 69 881)	-58.5 (-60.3 to -56.9)	184 169 (164 763 to 204 529)	-32.5 (-33.7 to -31.4)	1 122 043 (1 054 429 to 1 185 377)	-59.0 (-60.8 to -57.3)
Paraguay	989 (849 to 1 137)	-36.9 (-46.4 to -25.9)	3 969 (3 508 to 4 446)	-7.0 (-9.1 to -4.8)	15 831 (13 793 to 18 040)	-35.0 (-44.1 to -25.1)
**East Asia**	**749 124 (717 143 to 780 957)**	**-21.3 (-29.4 to -15.1)**	**3 851 577 (3 477 960 to 4 225 870)**	**17.5 (15.8 to 19.2)**	**16 663 455 (15 144 014 to 18 196 688)**	**-16.1 (-22.9 to -10.4)**
China	728 566 (697 508 to 759 228)	-20.7 (-29.0 to -14.2)	3 783 220 (3 416 264 to 4 147 506)	18.3 (16.6 to 20.0)	16 215 916 (14 726 373	-15.7 (-22.7 to -9.7)

					to 17 715 604)	
North Korea	14 920 (12 783 to 17 082)	23.2 (4.9 to 48.4)	37 246 (33 147 to 41 576)	9.9 (7.7 to 12.0)	312 332 (271 616 to 353 012)	19.5 (3.5 to 38.0)
Taiwan (Province of China)	5 638 (4 838 to 6 510)	-71.6 (-75.6 to -67.3)	31 111 (27 369 to 35 111)	-27.1 (-29.0 to -25.3)	135 208 (113 688 to 155 858)	-60.2 (-65.3 to -55.5)
**Southeast Asia**	**173 120 (160 588 to 187 248)**	**-22.3 (-28.4 to -13.6)**	**499 431 (441 311 to 556 179)**	**-1.2 (-2.3 to -0.2)**	**3 462 907 (3 149 840 to 3 773 536)**	**-17.7 (-23.1 to -10.7)**
Cambodia	3 904 (3 345 to 4 521)	-17.8 (-32.0 to 5.7)	8 439 (7 442 to 9 439)	-3.8 (-5.7 to -1.9)	78 008 (67 667 to 90 024)	-18.6 (-31.5 to 0.6)
Indonesia	70 275 (62 575 to 79 272)	6.2 (-5.6 to 23.6)	207 160 (182 420 to 231 480)	12.5 (11.2 to 13.7)	1 481 907 (1 315 216 to 1 660 088)	9.6 (-0.1 to 22.3)
Laos	1 710 (1 437 to 1 991)	-20.7 (-32.1 to -4.1)	3 709 (3 287 to 4 160)	-5.3 (-7.2 to -3.3)	35 069 (30 203 to 40 224)	-22.5 (-33.2 to -8.7)
Malaysia	4 704 (4 189 to 5 301)	-44.4 (-51.4 to -35.8)	21 268 (18 637 to 23 964)	-9.4 (-11.3 to -7.5)	109 214 (96 030 to 122 920)	-40.8 (-47.6 to -33.5)
Maldives	29 (24 to 36)	-65.2 (-73.2 to -54.8)	190 (165 to 213)	-17.5 (-19.7 to -15.3)	699 (573 to 830)	-56.6 (-64.6 to -46.9)
Mauritius	270 (236 to 310)	-65.9 (-70.3 to -60.7)	1 150 (1 009 to 1 302)	-27.9 (-29.7 to -26.2)	5 752 (5 014 to 6 551)	-61.7 (-66.3 to -56.6)
Myanmar	15 118 (13 221 to 17 494)	-31.5 (-42.4 to -16.3)	40 155 (35 397 to 45 232)	-8.4 (-10.4 to -6.7)	307 960 (268 514 to 355 569)	-30.6 (-41.1 to -17.6)
Philippines	18 137 (15 752 to 20 640)	0.6 (-12.5 to 15.4)	56 992 (50 324 to 64 369)	17.9 (14.7 to 20.7)	392 771 (342 939 to 443 419)	10.3 (-2.5 to 25.0)
Sri Lanka	6 202 (5 086 to 7 455)	-39.4 (-51.1 to -25.5)	19 149 (16 916 to 21 494)	-8.5 (-10.5 to -6.4)	114 927 (96 432 to 134 818)	-30.7 (-41.7 to -17.7)
Seychelles	17 (15 to 20)	-34.3 (-44.0 to -23.4)	79 (70 to 90)	-4.1 (-6.1 to -2.4)	376 (325 to 431)	-30.0 (-39.4 to -21.2)
Thailand	12 242 (10 816 to 13 755)	-46.9 (-53.8 to -39.2)	63 314 (55 535 to 71 295)	-6.4 (-8.3 to -4.5)	276 400 (239 954 to 312 554)	-41.6 (-48.1 to -34.9)

Timor-Leste	179 (140 to 225)	-26.3 (-44.0 to 3.6)	587 (513 to 666)	2.7 (0.7 to 4.6)	3 710 (3 010 to 4 534)	-25.0 (-42.3 to -0.1)
Vietnam	40 332 (35 787 to 45 089)	-29.4 (-40.6 to -13.4)	76 239 (68 089 to 84 310)	-14.1 (-15.9 to -12.6)	654 666 (580 765 to 734 367)	-26.9 (-38.3 to -12.9)
**Oceania**	**2 636 (2 244 to 3 036)**	**-19.8 (-29.3 to -7.3)**	**7 542 (6 707 to 8 406)**	**-4.1 (-5.6 to -2.6)**	**72 773 (62 842 to 83 649)**	**-17.7 (-27.8 to -5.2)**
American Samoa	8 (7 to 10)	-33.9 (-45.2 to -20.2)	48 (42 to 54)	-8.9 (-10.9 to -7.2)	254 (214 to 297)	-30.1 (-40.0 to -18.8)
Federated States of Micronesia	34 (27 to 42)	-19.2 (-35.9 to 0.8)	75 (66 to 84)	-5.0 (-7.0 to -3.0)	795 (633 to 976)	-17.5 (-35.4 to 2.8)
Fiji	207 (163 to 259)	-18.8 (-38.8 to 7.5)	761 (666 to 853)	-3.0 (-5.1 to -0.9)	5 500 (4 476 to 6 704)	-16.6 (-35.7 to 6.2)
Guam	56 (49 to 64)	-9.4 (-23.2 to 6.9)	194 (172 to 217)	1.7 (-0.5 to 3.8)	1 283 (1 118 to 1 454)	-7.1 (-18.6 to 6.7)
Kiribati	43 (37 to 49)	-14.1 (-26.1 to 1.0)	87 (77 to 97)	-5.2 (-7.1 to -3.3)	1 146 (1 005 to 1 325)	-12.8 (-25.7 to 2.2)
Marshall Islands	13 (11 to 15)	-21.0 (-33.4 to -5.9)	42 (38 to 47)	-1.4 (-3.6 to 0.8)	365 (309 to 429)	-17.8 (-29.8 to -2.8)
Northern Mariana Islands	6 (5 to 7)	-39.7 (-52.2 to -24.2)	49 (42 to 56)	-11.1 (-13.0 to -9.5)	224 (185 to 267)	-33.2 (-44.7 to -19.9)
Papua New Guinea	1 915 (1 546 to 2 294)	-16.1 (-30.1 to 2.7)	4 624 (4 098 to 5 170)	-3.8 (-5.6 to -1.7)	53 365 (43 244 to 63 838)	-17.1 (-30.6 to 0.3)
Samoa	47 (38 to 57)	-30.7 (-42.7 to -16.8)	143 (128 to 160)	-9.0 (-11.0 to -7.2)	1 019 (840 to 1 198)	-28.8 (-40.1 to -16.6)
Solomon Islands	185 (151 to 225)	-14.6 (-28.1 to 1.1)	368 (327 to 411)	-6.0 (-8.0 to -4.3)	4 621 (3 783 to 5 663)	-14.7 (-28.7 to 2.1)
Tonga	23 (19 to 26)	-22.6 (-36.2 to -5.2)	73 (65 to 81)	-2.5 (-4.3 to -0.6)	461 (398 to 530)	-20.5 (-33.4 to -6.8)
Vanuatu	99 (79 to 120)	-15.4 (-29.7 to 2.5)	207 (184 to 231)	-10.5 (-12.3 to -8.8)	2 452 (1 981 to 3 026)	-15.8 (-30.6 to 1.7)
**North Africa and Middle East**	**136 922 (124 993 to 149 093)**	**-20.8 (-27.2 to -11.0)**	**439 579 (392 288 to 489 787)**	**-3.2 (-4.5 to -2.0)**	**2 847 125 (2 584 804**	**-20.4 (-26.2 to -12.3)**

					**to 3 104 675)**	
Afghanistan	10 024 (7 929 to 12 180)	4.6 (-8.7 to 23.0)	18 620 (16 596 to 20 866)	0.5 (-1.8 to 2.6)	238 376 (190 067 to 292 571)	1.0 (-12.5 to 17.2)
Algeria	9 724 (8 231 to 11 274)	-27.0 (-36.2 to -15.3)	33 300 (29 477 to 37 460)	-9.8 (-11.7 to -8.0)	174 455 (150 221 to 200 299)	-29.4 (-37.7 to -20.0)
Bahrain	72 (59 to 88)	-43.4 (-54.5 to -30.0)	717 (618 to 819)	-10.6 (-12.7 to -8.6)	2 160 (1 820 to 2 536)	-41.4 (-50.9 to -30.5)
Egypt	30 884 (26 430 to 36 182)	-25.1 (-35.9 to -9.5)	80 581 (71 381 to 90 082)	2.6 (0.1 to 4.9)	641 552 (556 526 to 747 062)	-17.2 (-29.0 to -1.0)
Iran	22 499 (19 152 to 26 353)	-28.1 (-42.2 to -9.4)	67 201 (59 503 to 74 827)	-17.7 (-19.5 to -16.0)	449 904 (384 777 to 525 440)	-30.2 (-43.3 to -13.9)
Iraq	8 644 (7 194 to 10 231)	-15.6 (-32.7 to 4.1)	24 283 (21 684 to 27 257)	-6.4 (-8.6 to -4.4)	199 425 (166 739 to 235 586)	-15.3 (-32.4 to 3.6)
Jordan	1 426 (1 125 to 1 797)	-40.4 (-53.1 to -24.5)	4 938 (4 390 to 5 533)	-18.1 (-20.3 to -16.2)	28 268 (23 050 to 34 848)	-41.5 (-54.1 to -27.4)
Kuwait	280 (211 to 361)	21.1 (-8.1 to 57.9)	2 037 (1 761 to 2 333)	16.9 (14.0 to 19.4)	8 245 (6 573 to 10 200)	13.1 (-9.4 to 39.6)
Lebanon	748 (568 to 984)	-65.8 (-75.4 to -52.2)	5 255 (4 617 to 5 957)	-15.2 (-17.6 to -13.0)	15 917 (12 703 to 19 409)	-62.8 (-71.3 to -52.5)
Libya	1 091 (877 to 1 368)	-2.9 (-18.2 to 14.8)	4 880 (4 304 to 5 470)	12.4 (9.8 to 14.9)	24 016 (19 809 to 29 166)	-3.1 (-16.1 to 11.1)
Morocco	9 063 (7 206 to 11 238)	-20.9 (-33.9 to 2.6)	32 025 (28 277 to 35 996)	-0.7 (-2.7 to 1.4)	171 547 (141 020 to 204 519)	-21.8 (-33.2 to -4.4)
Palestine	1 439 (1 325 to 1 555)	302.0 (244.2 to 377.4)	2 680 (2 404 to 2 969)	79.5 (75.3 to 83.5)	25 774 (23 836 to 27 890)	225.3 (181.2 to 279.2)
Oman	110 (95 to 127)	-29.4 (-46.2 to -6.8)	2 015 (1 730 to 2 312)	10.1 (8.1 to 12.2)	3 855 (3 186 to 4 533)	-19.3 (-32.7 to -3.9)
Qatar	38 (28 to 52)	-58.9 (-68.7 to -46.0)	951 (807 to 1 107)	-9.1 (-11.4 to -6.7)	1 931 (1 508 to 2 381)	-50.7 (-60.0 to -39.8)

Saudi Arabia	5 442 (4 911 to 6 017)	-17.2 (-29.9 to 0.5)	19 917 (17 580 to 22 454)	-4.2 (-5.4 to -3.1)	111 774 (100 290 to 123 667)	-16.8 (-28.5 to -2.6)
Sudan	8 733 (7 048 to 10 411)	-19.3 (-29.3 to -7.1)	23 810 (21 235 to 26 549)	-4.1 (-6.2 to -2.1)	191 799 (155 508 to 228 228)	-22.2 (-31.5 to -12.6)
Syria	2 447 (2 179 to 2 758)	-41.7 (-50.5 to -30.5)	10 853 (9 622 to 12 157)	-12.3 (-14.3 to -10.4)	58 090 (51 828 to 65 805)	-40.1 (-48.3 to -30.6)
Tunisia	3 581 (2 831 to 4 389)	-28.0 (-41.7 to -12.3)	12 130 (10 680 to 13 606)	-1.9 (-3.9 to 0.0)	59 300 (48 626 to 71 097)	-26.8 (-39.4 to -12.7)
Turkey	13 551 (11 304 to 16 127)	-12.2 (-30.7 to 12.1)	72 844 (64 174 to 81 856)	8.0 (5.9 to 10.1)	261 808 (224 220 to 302 780)	-18.3 (-31.2 to -2.2)
United Arab Emirates	1 071 (839 to 1 376)	-22.3 (-40.2 to 2.8)	5 536 (4 804 to 6 378)	-19.5 (-21.2 to -17.9)	38 954 (30 911 to 49 723)	-22.4 (-40.1 to -0.3)
Yemen	6 054 (5 026 to 7 115)	-13.9 (-25.9 to 2.3)	14 612 (12 942 to 16 326)	-6.9 (-9.0 to -5.1)	139 540 (117 884 to 162 588)	-17.5 (-28.8 to -3.1)
**South Asia**	**493 871 (454 668 to 537 483)**	**-14.7 (-22.5 to -5.3)**	**1 059 418 (942 765 to 1 177 770)**	**1.2 (0.1 to 2.3)**	**10 094 867 (9 308 582 to 10 988 007)**	**-15.6 (-22.3 to -8.0)**
Bangladesh	66 570 (57 603 to 76 545)	-21.5 (-33.5 to -6.7)	109 129 (97 262 to 121 102)	-5.8 (-7.8 to -3.8)	1 254 444 (1 070 559 to 1 476 947)	-17.8 (-30.3 to -2.5)
Bhutan	149 (122 to 179)	-40.1 (-51.5 to -26.3)	398 (352 to 447)	-11.2 (-13.1 to -9.5)	2 715 (2 212 to 3 242)	-39.8 (-51.5 to -27.2)
India	359 456 (328 175 to 392 557)	-16.4 (-24.1 to -7.2)	819 601 (727 687 to 911 905)	2.6 (1.5 to 3.6)	7 530 757 (6 891 541 to 8 262 600)	-17.2 (-23.6 to -9.6)
Nepal	7 679 (6 506 to 9 040)	-17.5 (-31.9 to 2.8)	17 454 (15 462 to 19 521)	1.2 (-0.6 to 3.1)	151 678 (130 202 to 176 772)	-20.1 (-33.1 to -3.7)
Pakistan	60 016 (50 269 to 70 905)	-4.6 (-22.4 to 18.2)	112 836 (99 777 to 126 560)	-2.6 (-4.7 to -0.5)	1 155 273 (975 285 to 1 351 624)	-5.0 (-22.2 to 15.1)
**Southern sub-Saharan Africa**	**13 814 (12 914 to 14 691)**	**0.6 (-7.9 to 11.9)**	**42 080 (37 575 to 46 844)**	**5.5 (4.3 to 6.7)**	**256 993 (237 894 to 274 440)**	**-1.9 (-9.9 to 7.4)**

Botswana	372 (190 to 521)	-22.7 (-58.6 to 9.0)	1 082 (949 to 1 226)	-5.2 (-7.2 to -3.2)	7 435 (4 205 to 10 251)	-21.4 (-55.8 to 9.7)
Lesotho	637 (468 to 824)	22.5 (-12.1 to 64.4)	1 014 (893 to 1 140)	14.5 (12.2 to 17.0)	10 926 (8 275 to 14 242)	24.4 (-10.4 to 64.5)
Namibia	378 (254 to 495)	-43.5 (-60.7 to -27.8)	1 081 (954 to 1 216)	-20.7 (-22.6 to -18.9)	7 143 (5 092 to 9 093)	-44.6 (-59.9 to -29.9)
South Africa	10 382 (9 611 to 11 112)	11.7 (1.3 to 24.3)	33 158 (29 663 to 36 915)	7.0 (5.7 to 8.2)	193 250 (179 104 to 207 060)	5.1 (-3.8 to 15.0)
Swaziland	239 (156 to 332)	-19.3 (-43.2 to 8.6)	563 (492 to 639)	2.0 (-0.5 to 4.1)	4 557 (3 073 to 6 224)	-18.4 (-41.4 to 9.2)
Zimbabwe	1 805 (1 404 to 2 213)	-30.4 (-47.6 to 5.5)	5 182 (4 526 to 5 841)	-0.6 (-2.7 to 1.4)	33 682 (26 944 to 40 431)	-21.0 (-40.6 to 17.0)
**Western sub-Saharan Africa**	**41 489 (37 158 to 45 415)**	**-19.7 (-27.8 to -9.8)**	**134 878 (119 064 to 151 988)**	**-3.0 (-4.5 to -1.6)**	**912 350 (822 421 to 1 000 638)**	**-21.0 (-29.2 to -12.1)**
Benin	1 534 (1 267 to 1 791)	-10.1 (-22.9 to 3.9)	3 938 (3 458 to 4 421)	-2.5 (-4.4 to -0.4)	33 737 (27 944 to 39 261)	-11.0 (-23.3 to 2.3)
Burkina Faso	1 464 (1 130 to 1 807)	10.7 (-8.1 to 34.4)	5 115 (4 470 to 5 848)	11.2 (8.8 to 13.6)	33 138 (26 435 to 40 150)	5.8 (-10.6 to 26.0)
Cameroon	3 563 (2 644 to 4 570)	-5.5 (-27.4 to 19.2)	8 208 (7 218 to 9 229)	-1.1 (-3.2 to 1.1)	71 163 (53 117 to 90 538)	-4.1 (-25.5 to 19.3)
Cape Verde	95 (81 to 108)	-33.6 (-43.8 to -22.0)	284 (251 to 319)	-8.9 (-11.0 to -7.1)	1 630 (1 404 to 1 833)	-32.7 (-42.3 to -21.7)
Chad	1 578 (1 241 to 1 911)	-15.5 (-29.3 to -0.2)	4 474 (3 916 to 5 050)	-0.7 (-2.9 to 1.2)	33 671 (26 652 to 40 297)	-15.6 (-28.7 to -1.7)
Cote d'Ivoire	3 738 (3 175 to 4 332)	-11.1 (-23.1 to 4.1)	9 119 (8 062 to 10 268)	-6.6 (-8.7 to -4.7)	85 876 (73 322 to 99 926)	-11.5 (-23.2 to 2.7)
The Gambia	179 (144 to 218)	-20.7 (-35.5 to -4.1)	649 (569 to 730)	-2.9 (-4.8 to -1.0)	4 026 (3 312 to 4 786)	-21.3 (-35.5 to -6.4)
Ghana	6 627 (5 612 to 7 599)	-5.5 (-22.7 to 14.9)	12 382 (11 006 to 13 841)	-1.6 (-3.7 to 0.4)	130 207 (110 531 to 148 979)	-8.0 (-25.2 to 11.3)

Guinea	2 285<br>(1 901 to 2 675)	-4.1<br>(-20.9 to 14.0)	4 894<br>(4 347 to 5 507)	0.5<br>(-1.5 to 2.4)	48 606<br>(40 916 to 56 794)	-4.9<br>(-21.2 to 12.7)
Guinea-Bissau	415<br>(356 to 481)	-15.4<br>(-29.2 to 2.6)	829<br>(735 to 931)	-6.1<br>(-8.2 to -4.0)	9 326<br>(7 962 to 10 772)	-17.1<br>(-30.6 to -0.4)
Liberia	622<br>(538 to 710)	-2.5<br>(-17.5 to 16.1)	1 702<br>(1 502 to 1 911)	2.5<br>(0.5 to 4.5)	13 378<br>(11 667 to 15 224)	-3.8<br>(-18.1 to 13.4)
Mali	1 912<br>(1 481 to 2 450)	-33.7<br>(-47.0 to -17.2)	5 329<br>(4 682 to 6 027)	-11.0<br>(-12.9 to -9.2)	41 625<br>(32 813 to 52 749)	-35.8<br>(-48.5 to -20.1)
Mauritania	536<br>(386 to 720)	-42.6<br>(-54.9 to -27.0)	1 704<br>(1 514 to 1 918)	-18.7<br>(-20.6 to -17.0)	11 514<br>(8 730 to 14 996)	-44.5<br>(-56.3 to -30.6)
Niger	2 227<br>(1 485 to 2 876)	-13.1<br>(-31.9 to 7.9)	6 674<br>(5 851 to 7 585)	-0.2<br>(-2.3 to 1.8)	50 467<br>(35 006 to 64 601)	-13.6<br>(-30.7 to 7.1)
Nigeria	10 616<br>(8 409 to 13 114)	-41.0<br>(-53.1 to -26.5)	58 403<br>(50 797 to 66 346)	-5.7<br>(-8.1 to -3.6)	252 724<br>(207 316 to 303 922)	-39.9<br>(-51.8 to -26.8)
Sao Tome and Principe	41<br>(35 to 48)	-1.7<br>(-19.7 to 17.9)	84<br>(75 to 94)	-4.2<br>(-6.3 to -2.2)	717<br>(604 to 832)	-4.3<br>(-21.8 to 14.7)
Senegal	2 127<br>(1 791 to 2 488)	-4.9<br>(-18.1 to 10.0)	5 876<br>(5 212 to 6 607)	0.3<br>(-1.8 to 2.3)	45 063<br>(38 184 to 52 611)	-6.7<br>(-18.8 to 6.7)
Sierra Leone	878<br>(747 to 1 011)	-0.2<br>(-15.0 to 17.3)	2 466<br>(2 158 to 2 766)	5.0<br>(2.7 to 7.1)	21 556<br>(18 603 to 24 700)	-0.1<br>(-15.2 to 16.0)
Togo	1 052<br>(847 to 1 247)	-12.9<br>(-26.2 to 2.3)	2 741<br>(2 409 to 3 084)	-2.7<br>(-4.7 to -0.8)	23 920<br>(19 718 to 28 135)	-12.5<br>(-25.1 to 3.1)
**Eastern sub-Saharan Africa**	**46 002<br>(39 968 to 51 920)**	**-32.4<br>(-39.8 to -22.8)**	**124 296<br>(109 567 to 139 265)**	**-9.0<br>(-10.2 to -7.7)**	**925 823<br>(811 263 to 1 036 606)**	**-33.0<br>(-40.2 to -24.9)**
Burundi	1 416<br>(1 141 to 1 752)	-48.5<br>(-59.5 to -34.1)	3 402<br>(2 991 to 3 828)	-24.6<br>(-26.3 to -23.1)	28 934<br>(23 466 to 35 292)	-51.0<br>(-61.3 to -38.2)
Comoros	79<br>(63 to 96)	-44.5<br>(-54.9 to -31.7)	257<br>(225 to 290)	-20.9<br>(-22.8 to -19.0)	1 676<br>(1 367 to 2 010)	-46.2<br>(-55.9 to -34.6)
Djibouti	125<br>(88 to 162)	-23.2<br>(-41.0 to -0.7)	397<br>(348 to 446)	-5.0<br>(-7.0 to -3.1)	2 572<br>(1 853 to 3 292)	-23.4<br>(-41.1 to -4.7)

Eritrea	682 (520 to 845)	-41.1 (-51.0 to -29.2)	1 518 (1 323 to 1 718)	-16.8 (-18.6 to -14.9)	14 072 (10 981 to 17 123)	-44.2 (-53.2 to -33.4)
Ethiopia	12 020 (9 609 to 15 111)	-40.4 (-52.5 to -23.5)	33 000 (29 027 to 37 231)	-10.9 (-13.0 to -8.8)	243 761 (200 104 to 300 568)	-40.4 (-52.0 to -25.7)
Kenya	3 616 (2 920 to 4 381)	-15.9 (-27.0 to -1.1)	14 128 (12 347 to 15 927)	0.8 (-0.2 to 1.9)	73 330 (59 879 to 87 483)	-14.9 (-25.3 to -2.7)
Madagascar	5 229 (4 096 to 6 387)	-15.9 (-34.4 to 3.8)	9 662 (8 529 to 10 810)	-11.8 (-13.9 to -9.9)	109 727 (87 027 to 134 234)	-17.4 (-35.6 to 0.6)
Malawi	2 087 (1 596 to 2 643)	-21.6 (-39.3 to 2.2)	5 946 (5 222 to 6 707)	-4.0 (-6.0 to -2.0)	40 902 (32 159 to 51 061)	-20.7 (-38.8 to 0.7)
Mozambique	4 638 (3 704 to 5 755)	-32.1 (-44.5 to -15.9)	11 116 (9 803 to 12 459)	-6.8 (-9.0 to -4.7)	92 277 (74 601 to 114 866)	-30.9 (-43.6 to -16.0)
Rwanda	1 083 (785 to 1 430)	-57.0 (-67.4 to -44.1)	3 376 (2 950 to 3 812)	-26.1 (-27.9 to -24.3)	21 197 (15 371 to 27 572)	-59.1 (-69.2 to -47.7)
Somalia	1 832 (1 481 to 2 254)	-24.7 (-37.1 to -8.8)	3 705 (3 244 to 4 156)	-9.5 (-11.4 to -7.7)	37 140 (30 286 to 45 374)	-26.6 (-38.6 to -11.2)
South Sudan	1 683 (1 242 to 2 162)	-15.6 (-32.6 to 7.7)	4 726 (4 154 to 5 364)	-5.2 (-7.3 to -3.5)	33 948 (25 464 to 42 874)	-16.5 (-33.9 to 5.6)
Tanzania	5 640 (4 552 to 6 784)	-25.9 (-38.6 to -9.6)	17 747 (15 578 to 20 004)	-2.0 (-4.1 to 0.1)	111 041 (89 512 to 133 806)	-25.4 (-38.4 to -10.6)
Uganda	3 781 (2 988 to 4 569)	-38.4 (-50.4 to -23.9)	9 849 (8 618 to 11 144)	-14.8 (-16.7 to -13.0)	73 386 (59 007 to 88 159)	-40.3 (-52.2 to -27.3)
Zambia	2 090 (1 596 to 2 624)	4.4 (-21.5 to 40.1)	5 372 (4 730 to 6 060)	3.7 (1.7 to 5.6)	41 769 (32 600 to 51 604)	8.7 (-18.3 to 41.1)
**Central sub-Saharan Africa**	**17 763 (14 734 to 20 438)**	**-18.3 (-28.7 to -5.2)**	**39 764 (34 993 to 44 723)**	**-7.4 (-8.9 to -5.8)**	**345 734 (293 024 to 393 121)**	**-20.0 (-30.0 to -8.8)**
Angola	2 696 (2 039 to 3 600)	-25.7 (-43.0 to -2.7)	7 778 (6 848 to 8 791)	-10.4 (-12.2 to -8.4)	56 513 (43 654 to 73 621)	-27.5 (-44.3 to -5.9)
Central African Republic	1 584 (1 177 to 2 000)	-6.2 (-24.6 to 13.2)	2 389 (2 108 to 2 673)	-2.7 (-4.7 to -0.6)	30 518 (23 480 to 38 132)	-6.5 (-24.9 to 13.2)

Congo (Brazzaville)	876<br>(689 to 1 086)	-39.7<br>(-52.7 to -24.0)	1 962<br>(1 722 to 2 202)	-20.4<br>(-22.1 to -18.4)	16 591<br>(13 263 to 20 263)	-41.6<br>(-54.7 to -27.5)
DR Congo	12 177<br>(9 717 to 14 501)	-12.7<br>(-26.4 to 3.9)	26 422<br>(23 161 to 29 745)	-4.1<br>(-6.1 to -2.1)	234 372<br>(190 889 to 274 684)	-14.0<br>(-27.4 to 0.8)
Equatorial Guinea	73<br>(47 to 105)	-68.2<br>(-78.9 to -53.6)	352<br>(307 to 399)	-21.2<br>(-23.2 to -19.4)	1 606<br>(1 108 to 2 222)	-67.7<br>(-78.2 to -54.8)
Gabon	357<br>(282 to 434)	-36.7<br>(-49.5 to -21.6)	861<br>(759 to 973)	-20.3<br>(-22.0 to -18.6)	6 134<br>(4 964 to 7 365)	-38.3<br>(-50.1 to -24.6)

Table A8: Incidence, deaths and DALYs for 2016 and percentage change of age-standardized rates by location for Haemorrhagic stroke

Location	Deaths (95% UI)		Incidence (95% UI)		DALYs (95% UI)	
	2016 counts	Percentage change in age-standardised rates between 1990 and 2016	2016 counts	Percentage change in age-standardised rates between 1990 and 2016	2016 counts	Percentage change in age-standardised rates between 1990 and 2016
**Global**	**2 838 061<br>(2 748 568 to 2 934 055)**	**-37.8<br>(-41.7 to -34.9)**	**4 120 318<br>(3 763 946 to 4 508 361)**	**-14.0<br>(-15.1 to -12.9)**	**64 547 699<br>(62 622 343 to 66 497 593)**	**-37.9<br>(-41.3 to -35.2)**
**High SDI**	**291 386<br>(273 987 to 310 091)**	**-48.0<br>(-49.9 to -46.3)**	**704 823<br>(646 876 to 762 326)**	**-20.3<br>(-21.4 to -19.3)**	**4 896 000<br>(4 640 190 to 5 153 933)**	**-49.9<br>(-51.6 to -48.2)**

**High-middle SDI**	**418 416 (390 452 to 451 306)**	**-47.4 (-51.2 to -43.2)**	**789 958 (718 670 to 867 704)**	**-20.6 (-21.9 to -19.3)**	**8 694 262 (8 099 022 to 9 350 778)**	**-47.4 (-51.4 to -43.1)**
**Middle SDI**	**1 304 666 (1 261 170 to 1 350 095)**	**-44.6 (-50.1 to -40.7)**	**1 767 307 (1 614 289 to 1 934 319)**	**-14.7 (-15.8 to -13.6)**	**29 305 540 (28 390 404 to 30 220 498)**	**-44.5 (-49.2 to -40.8)**
**Low-middle SDI**	**631 012 (592 885 to 669 333)**	**-29.2 (-34.9 to -24.0)**	**671 264 (609 117 to 736 888)**	**-13.7 (-14.6 to -12.7)**	**16 271 025 (15 374 226 to 17 161 858)**	**-30.5 (-35.7 to -25.7)**
**Low SDI**	**190 889 (176 800 to 206 045)**	**-23.3 (-28.3 to -16.4)**	**153 831 (139 253 to 169 327)**	**-17.6 (-18.6 to -16.5)**	**5 323 598 (4 939 799 to 5 698 179)**	**-26.1 (-30.8 to -20.0)**
**High-income North America**	**69 738 (66 487 to 73 097)**	**-12.4 (-15.9 to -8.9)**	**210 236 (192 256 to 228 382)**	**-5.5 (-6.6 to -4.3)**	**1 409 270 (1 342 473 to 1 480 726)**	**-19.0 (-22.2 to -16.2)**
Canada	5 801 (5 308 to 6 366)	-31.5 (-38.2 to -23.9)	20 395 (18 266 to 22 442)	-9.9 (-11.9 to -7.9)	102 919 (94 456 to 111 619)	-37.6 (-42.7 to -32.1)
Greenland	20 (16 to 26)	-52.3 (-60.7 to -41.6)	29 (26 to 33)	-36.7 (-38.2 to -35.2)	484 (387 to 609)	-53.2 (-62.5 to -42.2)
USA	63 916 (61 077 to 67 101)	-10.3 (-13.9 to -6.4)	189 745 (173 341 to 206 083)	-4.9 (-6.1 to -3.8)	1 305 812 (1 247 144 to 1 371 159)	-17.0 (-20.4 to -14.1)
**Australasia**	**8 237 (7 409 to 9 049)**	**-44.6 (-49.5 to -39.6)**	**15 235 (13 955 to 16 650)**	**-22.9 (-24.4 to -21.5)**	**104 244 (96 070 to 112 297)**	**-51.2 (-55.0 to -47.3)**
Australia	6 906 (6 178 to 7 642)	-44.0 (-49.6 to -38.4)	12 409 (11 223 to 13 674)	-24.5 (-26.1 to -22.7)	85 985 (78 641 to 93 331)	-50.6 (-54.9 to -45.9)
New Zealand	1 331 (1 176 to 1 506)	-46.9 (-52.8 to -40.1)	2 826 (2 652 to 3 001)	-15.5 (-17.4 to -13.6)	18 259 (16 281 to 20 256)	-53.7 (-58.5 to -48.4)
**High-income Asia-Pacific**	**63 100 (57 893 to 68 358)**	**-59.8 (-63.1 to -56.1)**	**137 683 (124 197 to 151 005)**	**-32.5 (-33.7 to -31.2)**	**1 111 207 (1 016 004 to 1 212 988)**	**-59.9 (-63.4 to -55.8)**
Brunei	78 (64 to 92)	-44.3 (-53.8 to -33.5)	140 (125 to 156)	-31.3 (-32.7 to -29.9)	2 235 (1 816 to 2 683)	-46.9 (-57.0 to -35.4)
Japan	47 529 (44 147 to 50 754)	-52.8 (-55.1 to -50.6)	108 550 (98 118 to 119 061)	-26.0 (-27.3 to -24.8)	797 303 (747 403 to 848 266)	-50.3 (-52.6 to -48.0)
Singapore	545 (444 to 657)	-71.6 (-77.2 to -64.9)	1 735 (1 549 to 1 916)	-39.4 (-40.8 to -38.0)	12 844 (10 742 to 15 393)	-69.4 (-75.1 to -62.8)

South Korea	14 948 (11 322 to 19 000)	-75.5 (-81.4 to -67.6)	27 257 (24 200 to 30 373)	-53.6 (-54.8 to -52.4)	298 824 (228 946 to 378 874)	-76.9 (-82.7 to -69.7)
**Western Europe**	**142 194 (131 181 to 154 821)**	**-52.7 (-55.1 to -50.3)**	**328 031 (300 320 to 353 758)**	**-22.7 (-23.9 to -21.5)**	**1 973 987 (1 850 079 to 2 108 737)**	**-55.9 (-57.8 to -53.9)**
Andorra	26 (21 to 32)	-36.0 (-51.0 to -16.7)	65 (58 to 73)	-13.4 (-15.1 to -11.7)	348 (282 to 421)	-35.7 (-50.9 to -18.3)
Austria	1 712 (1 489 to 1 962)	-68.5 (-71.5 to -65.0)	7 123 (6 427 to 7 846)	-34.3 (-35.9 to -32.6)	28 283 (25 376 to 31 165)	-65.7 (-69.0 to -62.6)
Belgium	3 936 (3 447 to 4 466)	-44.3 (-50.8 to -37.5)	8 418 (8 006 to 8 908)	-21.9 (-23.6 to -20.3)	55 789 (49 846 to 62 026)	-48.7 (-54.4 to -42.8)
Cyprus	248 (217 to 278)	-59.4 (-65.3 to -52.4)	636 (574 to 700)	-23.0 (-24.7 to -21.2)	3 749 (3 387 to 4 132)	-58.7 (-64.2 to -52.5)
Denmark	1 952 (1 708 to 2 236)	-46.8 (-54.2 to -38.6)	4 149 (3 756 to 4 574)	-25.0 (-26.8 to -23.4)	28 543 (25 238 to 32 190)	-53.3 (-59.7 to -46.9)
Finland	1 460 (1 276 to 1 674)	-52.1 (-58.1 to -45.5)	4 711 (4 224 to 5 257)	-11.2 (-13.6 to -9.0)	25 378 (22 553 to 28 518)	-57.8 (-62.5 to -52.8)
France	18 135 (16 246 to 20 337)	-53.9 (-58.3 to -49.5)	42 370 (38 259 to 46 387)	-23.2 (-24.9 to -21.5)	254 100 (233 965 to 275 039)	-53.4 (-57.1 to -49.5)
Germany	22 983 (20 219 to 26 164)	-59.6 (-64.0 to -54.2)	66 774 (59 836 to 73 835)	-21.1 (-23.8 to -18.7)	350 996 (316 094 to 388 449)	-61.1 (-64.9 to -56.8)
Greece	7 405 (6 594 to 8 289)	-51.9 (-56.7 to -47.2)	11 968 (10 821 to 13 134)	-28.8 (-30.5 to -27.2)	96 933 (87 981 to 106 542)	-50.1 (-54.7 to -45.6)
Iceland	76 (66 to 86)	-40.5 (-47.9 to -33.0)	172 (156 to 189)	-22.5 (-24.2 to -20.7)	1 044 (927 to 1 156)	-49.9 (-55.2 to -44.2)
Ireland	945 (820 to 1 081)	-55.7 (-61.8 to -48.5)	2 535 (2 285 to 2 790)	-27.3 (-28.9 to -25.6)	15 616 (13 720 to 17 760)	-57.9 (-63.2 to -52.1)
Israel	1 371 (1 156 to 1 628)	-57.7 (-65.5 to -49.2)	3 742 (3 350 to 4 115)	-29.4 (-31.1 to -27.7)	21 662 (18 337 to 25 590)	-60.6 (-67.5 to -52.8)
Italy	25 149 (21 951 to 29 134)	-47.4 (-53.0 to -41.4)	55 098 (51 708 to 58 323)	-12.9 (-14.7 to -11.1)	315 409 (284 274 to 352 269)	-53.0 (-57.4 to -48.4)
Luxembourg	171 (149 to 193)	-63.6 (-67.9 to -58.9)	351 (321 to 386)	-41.9 (-43.3 to -40.5)	2 435 (2 169 to 2 729)	-66.3 (-70.1 to -62.1)

Malta	123 (103 to 144)	-58.5 (-65.4 to -50.6)	342 (307 to 379)	-23.1 (-25.0 to -21.2)	1 965 (1 677 to 2 294)	-60.2 (-66.1 to -53.2)
Netherlands	5 274 (4 709 to 5 889)	-40.1 (-46.3 to -32.7)	11 504 (10 352 to 12 651)	-19.0 (-20.9 to -17.2)	74 656 (67 807 to 82 617)	-46.4 (-51.6 to -40.5)
Norway	1 393 (1 206 to 1 589)	-55.8 (-61.4 to -49.9)	3 877 (3 486 to 4 256)	-19.6 (-22.0 to -17.6)	19 294 (17 081 to 21 591)	-57.2 (-62.3 to -51.5)
Portugal	6 752 (6 124 to 7 408)	-64.0 (-67.2 to -60.7)	9 344 (8 512 to 10 222)	-50.8 (-51.9 to -49.8)	93 022 (85 467 to 100 997)	-65.7 (-68.5 to -62.6)
Spain	14 561 (12 959 to 16 255)	-52.0 (-56.6 to -47.3)	34 132 (30 876 to 37 500)	-25.8 (-27.5 to -24.3)	195 811 (178 528 to 212 508)	-57.4 (-60.9 to -53.7)
Sweden	3 285 (2 819 to 3 795)	-37.6 (-45.1 to -29.2)	7 454 (6 613 to 8 264)	-12.5 (-14.6 to -10.4)	42 822 (37 604 to 48 278)	-44.2 (-50.7 to -37.0)
Switzerland	1 953 (1 527 to 2 475)	-61.7 (-69.6 to -51.5)	6 524 (5 819 to 7 216)	-16.0 (-18.5 to -13.7)	26 005 (21 016 to 31 823)	-62.4 (-69.5 to -54.4)
United Kingdom	23 286 (21 754 to 24 718)	-47.1 (-49.1 to -45.1)	46 441 (42 081 to 50 584)	-23.8 (-25.0 to -22.6)	319 917 (302 293 to 336 975)	-51.7 (-53.4 to -50.0)
**Southern Latin America**	**16 200 (14 800 to 17 672)**	**-37.4 (-43.2 to -30.7)**	**29 137 (26 395 to 31 832)**	**-19.7 (-21.1 to -18.2)**	**352 112 (322 245 to 384 926)**	**-41.0 (-46.2 to -34.6)**
Argentina	10 555 (9 536 to 11 567)	-37.8 (-44.0 to -30.7)	18 398 (16 669 to 20 104)	-21.7 (-23.4 to -20.1)	239 271 (216 796 to 261 108)	-41.7 (-47.3 to -35.1)
Chile	4 388 (3 500 to 5 453)	-36.8 (-49.5 to -21.0)	8 725 (7 837 to 9 649)	-15.1 (-17.1 to -13.0)	90 597 (71 533 to 113 137)	-39.3 (-52.4 to -23.9)
Uruguay	1 257 (1 150 to 1 365)	-32.9 (-39.1 to -26.0)	2 012 (1 830 to 2 206)	-19.8 (-21.5 to -18.1)	22 242 (20 444 to 24 088)	-36.5 (-42.5 to -30.2)
**Eastern Europe**	**119 249 (96 696 to 146 686)**	**-26.0 (-40.1 to -8.8)**	**189 417 (170 050 to 211 551)**	**-15.7 (-17.7 to -13.5)**	**2 540 743 (2 058 925 to 3 145 177)**	**-18.6 (-34.6 to 1.4)**
Belarus	4 088 (3 425 to 4 782)	-30.9 (-42.8 to -18.2)	7 827 (7 056 to 8 694)	-16.5 (-18.4 to -14.5)	93 408 (78 514 to 109 873)	-30.6 (-43.1 to -18.0)
Estonia	272 (221 to 337)	-68.9 (-74.6 to -61.4)	864 (771 to 964)	-33.6 (-35.6 to -31.8)	5 775 (4 741 to 6 964)	-67.4 (-72.9 to -61.0)

Latvia	887 (771 to 1 017)	-62.6 (-67.8 to -56.8)	2 080 (1 846 to 2 354)	-30.8 (-33.4 to -28.6)	16 910 (14 757 to 19 454)	-57.2 (-63.3 to -50.3)
Lithuania	916 (827 to 1 005)	-29.0 (-37.2 to -19.9)	2 497 (2 219 to 2 841)	-1.9 (-5.2 to 0.7)	19 237 (17 403 to 21 221)	-30.5 (-38.0 to -21.5)
Moldova	2 316 (2 048 to 2 597)	-36.4 (-44.9 to -26.3)	3 218 (2 890 to 3 569)	-27.7 (-29.3 to -26.2)	53 290 (47 073 to 59 988)	-34.6 (-43.2 to -24.5)
Russia	87 856 (66 274 to 115 435)	-21.6 (-41.0 to 3.8)	133 768 (119 450 to 149 396)	-13.7 (-16.2 to -11.2)	1 853 779 (1 393 393 to 2 447 712)	-12.5 (-35.9 to 17.5)
Ukraine	22 914 (18 573 to 28 504)	-35.6 (-49.0 to -18.0)	39 161 (35 135 to 43 769)	-20.9 (-22.8 to -19.0)	498 344 (401 626 to 619 748)	-28.8 (-44.1 to -9.7)
**Central Europe**	**68 623 (64 741 to 73 437)**	**-44.9 (-47.7 to -41.8)**	**96 693 (90 605 to 103 015)**	**-28.1 (-28.9 to -27.4)**	**1 209 781 (1 148 974 to 1 285 770)**	**-48.6 (-51.2 to -45.6)**
Albania	4 020 (3 470 to 4 529)	-6.5 (-18.8 to 5.1)	3 395 (3 086 to 3 722)	-14.8 (-16.6 to -12.9)	58 336 (50 576 to 65 629)	-13.5 (-24.6 to -3.0)
Bosnia and Herzegovina	1 407 (1 194 to 1 629)	-68.9 (-74.1 to -62.6)	2 850 (2 553 to 3 159)	-37.1 (-38.4 to -35.7)	27 678 (23 664 to 32 164)	-67.5 (-73.0 to -60.8)
Bulgaria	8 238 (7 129 to 9 450)	-47.4 (-55.3 to -38.8)	9 259 (8 330 to 10 188)	-34.0 (-35.5 to -32.4)	138 022 (118 756 to 158 546)	-49.1 (-57.0 to -40.7)
Croatia	3 162 (2 732 to 3 576)	-47.8 (-55.2 to -39.5)	3 831 (3 619 to 4 098)	-37.0 (-38.3 to -35.6)	50 981 (44 521 to 56 958)	-53.1 (-59.7 to -45.6)
Czech Republic	3 158 (2 885 to 3 443)	-72.3 (-75.0 to -69.4)	7 865 (7 012 to 8 808)	-34.5 (-36.4 to -32.8)	52 913 (48 487 to 57 421)	-72.4 (-74.9 to -69.6)
Hungary	3 583 (3 159 to 4 025)	-62.8 (-67.3 to -57.6)	7 340 (6 619 to 8 141)	-30.5 (-32.1 to -29.0)	70 289 (62 359 to 78 539)	-62.6 (-67.2 to -57.6)
Macedonia	2 022 (1 786 to 2 407)	-23.7 (-31.9 to -15.0)	2 127 (1 926 to 2 353)	-19.0 (-20.8 to -17.2)	37 422 (33 844 to 42 337)	-28.1 (-35.9 to -20.4)
Montenegro	1 268 (1 113 to 1 408)	-14.9 (-26.4 to -1.9)	972 (880 to 1 065)	-25.2 (-26.7 to -23.8)	18 752 (16 513 to 20 772)	-23.3 (-33.2 to -12.1)
Poland	11 942 (10 716 to 13 339)	-47.1 (-53.0 to -40.5)	22 531 (21 751 to 23 525)	-24.5 (-26.2 to -22.6)	249 417 (223 945 to 277 791)	-49.7 (-55.5 to -43.5)

Romania	22 240 (20 060 to 24 668)	-20.1 (-28.1 to -11.6)	23 460 (21 333 to 25 656)	-20.8 (-22.5 to -19.2)	367 655 (333 095 to 405 808)	-28.4 (-35.7 to -20.4)
Serbia	5 228 (4 579 to 6 334)	-48.6 (-55.4 to -40.9)	7 486 (7 203 to 7 785)	-31.6 (-33.2 to -30.0)	94 912 (84 925 to 109 089)	-50.2 (-56.6 to -43.6)
Slovakia	1 678 (1 460 to 1 911)	-58.6 (-64.9 to -51.9)	4 076 (3 732 to 4 439)	-17.9 (-19.9 to -16.3)	33 180 (28 911 to 37 811)	-58.6 (-64.6 to -52.1)
Slovenia	676 (574 to 791)	-58.1 (-64.5 to -51.2)	1 500 (1 413 to 1 568)	-27.2 (-28.9 to -25.7)	10 225 (8 862 to 11 640)	-64.0 (-69.4 to -58.4)
**Central Asia**	**42 839 (40 369 to 45 827)**	**-20.7 (-25.2 to -15.5)**	**52 616 (47 979 to 57 839)**	**-17.5 (-18.8 to -16.2)**	**972 551 (913 583 to 1 040 458)**	**-21.1 (-25.7 to -16.1)**
Armenia	922 (817 to 1 026)	-47.6 (-54.4 to -39.6)	1 926 (1 737 to 2 149)	-21.2 (-22.9 to -19.6)	18 664 (16 565 to 20 810)	-46.0 (-52.5 to -38.7)
Azerbaijan	5 624 (4 721 to 6 665)	-23.8 (-36.2 to -10.3)	6 951 (6 234 to 7 758)	-16.9 (-18.9 to -15.0)	117 538 (97 623 to 139 582)	-26.3 (-39.1 to -12.1)
Georgia	5 382 (4 618 to 6 153)	-27.9 (-39.5 to -15.4)	5 134 (4 685 to 5 623)	-23.3 (-24.8 to -21.7)	87 424 (74 386 to 101 711)	-31.8 (-43.0 to -19.7)
Kazakhstan	7 279 (6 175 to 8 737)	-28.4 (-39.7 to -13.3)	10 995 (9 912 to 12 185)	-20.8 (-22.7 to -19.0)	176 148 (148 436 to 210 473)	-28.0 (-39.4 to -12.8)
Kyrgyzstan	2 319 (2 097 to 2 546)	-34.5 (-41.9 to -26.3)	2 873 (2 610 to 3 174)	-29.5 (-31.2 to -28.0)	59 265 (53 410 to 65 291)	-31.6 (-39.3 to -23.5)
Mongolia	3 026 (2 639 to 3 446)	65.1 (40.7 to 95.4)	2 495 (2 265 to 2 738)	24.3 (22.2 to 26.3)	80 287 (69 095 to 92 565)	50.8 (26.1 to 79.5)
Tajikistan	2 642 (2 281 to 3 100)	-17.6 (-29.7 to -2.0)	3 320 (2 990 to 3 684)	-18.7 (-20.4 to -17.0)	60 607 (51 903 to 71 559)	-22.3 (-34.2 to -7.2)
Turkmenistan	3 869 (3 589 to 4 141)	-10.5 (-17.4 to -3.2)	3 880 (3 527 to 4 272)	-10.6 (-12.4 to -8.8)	101 298 (94 167 to 108 291)	-10.7 (-17.6 to -3.2)
Uzbekistan	11 776 (10 268 to 13 740)	-17.6 (-28.2 to -6.9)	15 042 (13 596 to 16 674)	-13.4 (-15.1 to -11.7)	271 321 (235 517 to 318 244)	-16.9 (-28.0 to -5.8)
**Central Latin America**	**36 444 (34 192 to 38 668)**	**-32.2 (-36.2 to -28.0)**	**69 681 (62 958 to 76 459)**	**-17.7 (-18.9 to -16.4)**	**916 646 (865 956 to 965 724)**	**-38.6 (-42.1 to -35.3)**

Colombia	7 551 (6 615 to 8 470)	-48.4 (-55.2 to -41.1)	14 248 (12 834 to 15 740)	-33.9 (-35.3 to -32.6)	170 869 (151 958 to 189 981)	-56.4 (-62.2 to -50.5)
Costa Rica	555 (495 to 625)	-32.8 (-40.3 to -24.2)	1 429 (1 293 to 1 576)	-10.4 (-12.2 to -8.7)	12 057 (10 878 to 13 347)	-34.5 (-41.5 to -27.1)
El Salvador	756 (665 to 858)	-59.4 (-64.8 to -53.3)	1 596 (1 434 to 1 774)	-30.0 (-31.7 to -28.5)	18 737 (16 527 to 21 293)	-66.5 (-70.7 to -61.6)
Guatemala	1 949 (1 524 to 2 429)	-4.8 (-26.2 to 21.3)	3 348 (3 021 to 3 660)	-0.6 (-2.7 to 1.5)	59 936 (47 178 to 74 330)	-19.5 (-38.0 to 3.2)
Honduras	1 343 (1 078 to 1 681)	-43.6 (-55.6 to -26.2)	2 003 (1 803 to 2 215)	-28.4 (-30.0 to -26.9)	55 507 (44 423 to 69 617)	-52.7 (-62.9 to -38.8)
Mexico	16 364 (15 344 to 17 261)	-26.2 (-29.9 to -22.8)	33 920 (30 814 to 37 226)	-10.2 (-11.5 to -8.8)	409 477 (389 565 to 428 025)	-29.8 (-33.2 to -26.8)
Nicaragua	793 (670 to 938)	-20.1 (-32.9 to -5.0)	1 545 (1 391 to 1 696)	-10.1 (-12.2 to -8.1)	18 692 (15 800 to 22 015)	-32.1 (-42.9 to -18.8)
Panama	796 (696 to 901)	-29.7 (-40.1 to -17.9)	1 360 (1 234 to 1 483)	-21.9 (-23.4 to -20.4)	16 708 (14 598 to 19 169)	-35.8 (-45.6 to -24.8)
Venezuela	6 337 (5 384 to 7 528)	-15.4 (-28.9 to 2.0)	10 234 (9 221 to 11 333)	-13.4 (-15.3 to -11.5)	154 663 (131 110 to 185 276)	-23.2 (-35.9 to -7.0)
**Andean Latin America**	**7 540 (6 726 to 8 438)**	**-47.5 (-54.0 to -40.2)**	**15 367 (13 885 to 16 852)**	**-22.7 (-24.1 to -21.3)**	**205 196 (184 630 to 230 872)**	**-55.1 (-61.0 to -48.5)**
Bolivia	2 279 (1 823 to 2 848)	-46.8 (-57.6 to -31.8)	3 300 (2 970 to 3 645)	-23.3 (-24.9 to -21.7)	61 719 (49 218 to 77 457)	-53.3 (-63.3 to -40.8)
Ecuador	2 179 (1 987 to 2 392)	-38.1 (-44.2 to -30.9)	4 175 (3 784 to 4 584)	-20.3 (-22.0 to -18.6)	57 662 (52 368 to 63 120)	-45.9 (-51.4 to -39.8)
Peru	3 082 (2 533 to 3 733)	-52.7 (-61.9 to -40.1)	7 892 (7 079 to 8 737)	-23.6 (-25.3 to -21.9)	85 816 (70 261 to 104 255)	-60.4 (-68.6 to -50.1)
**Caribbean**	**16 904 (15 427 to 18 368)**	**-20.7 (-27.1 to -13.8)**	**20 742 (18 890 to 22 732)**	**-16.3 (-17.6 to -15.0)**	**385 717 (344 936 to 428 532)**	**-31.7 (-39.2 to -24.7)**
Antigua and Barbuda	27 (24 to 31)	-39.6 (-48.1 to -29.8)	39 (35 to 43)	-25.0 (-26.7 to -23.5)	585 (512 to 663)	-41.8 (-50.0 to -32.4)

The Bahamas	140 (120 to 157)	-10.0 (-21.1 to 2.0)	177 (160 to 195)	-10.1 (-11.9 to -8.2)	3 105 (2 693 to 3 503)	-20.7 (-30.7 to -10.3)
Barbados	140 (126 to 156)	-22.8 (-31.6 to -12.3)	183 (165 to 201)	-15.4 (-17.0 to -13.7)	2 452 (2 222 to 2 731)	-26.0 (-34.5 to -15.8)
Belize	68 (59 to 78)	9.4 (-6.6 to 26.6)	88 (79 to 97)	-7.6 (-9.4 to -6.1)	1 693 (1 472 to 1 944)	-9.4 (-22.7 to 5.1)
Bermuda	15 (13 to 18)	-52.4 (-59.8 to -44.4)	25 (23 to 28)	-34.8 (-36.2 to -33.4)	271 (235 to 313)	-59.5 (-65.8 to -52.5)
Cuba	3 858 (3 480 to 4 215)	-25.2 (-33.4 to -16.7)	6 283 (5 704 to 6 898)	-15.9 (-17.7 to -14.2)	75 673 (68 569 to 83 022)	-33.1 (-40.4 to -25.3)
Dominica	26 (22 to 30)	-9.9 (-24.0 to 6.2)	33 (30 to 36)	-7.2 (-9.2 to -5.4)	508 (437 to 583)	-12.1 (-25.3 to 3.0)
Dominican Republic	2 906 (2 428 to 3 363)	-15.4 (-29.9 to -1.3)	3 813 (3 454 to 4 207)	-16.0 (-17.8 to -14.3)	60 762 (51 237 to 70 214)	-30.5 (-42.6 to -18.9)
Grenada	48 (42 to 54)	-14.0 (-27.2 to 0.1)	49 (45 to 54)	-11.0 (-13.0 to -9.2)	987 (854 to 1 123)	-20.0 (-33.7 to -5.6)
Guyana	424 (375 to 474)	-30.3 (-39.5 to -21.1)	366 (331 to 402)	-26.0 (-27.6 to -24.4)	10 902 (9 629 to 12 211)	-39.8 (-47.9 to -32.0)
Haiti	5 642 (4 412 to 7 014)	-24.7 (-39.1 to -7.4)	3 974 (3 596 to 4 397)	-25.0 (-26.7 to -23.4)	161 684 (124 701 to 203 268)	-35.0 (-48.2 to -19.7)
Jamaica	1 561 (1 330 to 1 808)	0.2 (-16.7 to 20.4)	1 604 (1 456 to 1 761)	-9.0 (-11.0 to -7.1)	27 535 (23 270 to 31 894)	-18.7 (-33.2 to -2.6)
Puerto Rico	1 041 (933 to 1 158)	10.5 (-2.2 to 23.9)	1 899 (1 712 to 2 090)	4.0 (2.0 to 5.9)	17 429 (15 650 to 19 276)	-6.8 (-17.6 to 5.0)
Saint Lucia	68 (61 to 74)	-36.5 (-43.1 to -29.6)	89 (81 to 98)	-21.3 (-22.8 to -19.7)	1 396 (1 274 to 1 516)	-39.9 (-46.2 to -33.7)
Saint Vincent and the Grenadines	50 (45 to 55)	2.6 (-10.3 to 16.2)	54 (49 to 59)	-3.0 (-4.8 to -1.3)	1 082 (976 to 1 188)	-6.2 (-17.5 to 5.7)
Suriname	293 (265 to 321)	22.7 (10.2 to 36.8)	294 (268 to 324)	-3.1 (-4.9 to -1.3)	6 668 (6 055 to 7 298)	3.2 (-7.6 to 15.1)
Trinidad and Tobago	544 (494 to 599)	-30.8 (-37.9 to -22.8)	718 (649 to 791)	-21.0 (-22.7 to -19.5)	11 591 (10 493 to 12 741)	-36.4 (-42.9 to -28.9)

Virgin Islands	53 (46 to 61)	-19.9 (-32.3 to -5.7)	73 (66 to 82)	-12.8 (-14.5 to -11.2)	958 (819 to 1 094)	-28.4 (-39.5 to -15.8)
**Tropical Latin America**	**43 838 (41 676 to 45 854)**	**-49.9 (-52.1 to -47.7)**	**76 724 (69 439 to 84 297)**	**-27.6 (-28.7 to -26.6)**	**1 169 071 (1 115 344 to 1 221 059)**	**-53.9 (-55.8 to -51.9)**
Brazil	41 386 (39 281 to 43 382)	-51.0 (-53.2 to -48.8)	73 852 (66 806 to 81 171)	-27.8 (-28.9 to -26.7)	1 114 697 (1 061 996 to 1 166 155)	-54.8 (-56.7 to -52.8)
Paraguay	2 452 (2 146 to 2 773)	-12.5 (-24.1 to 0.7)	2 871 (2 614 to 3 153)	-16.4 (-18.2 to -14.6)	54 374 (48 577 to 61 108)	-17.2 (-27.8 to -4.9)
**East Asia**	**1 099 808 (1 060 910 to 1 141 084)**	**-51.9 (-57.3 to -47.9)**	**1 767 939 (1 616 849 to 1 939 605)**	**-14.6 (-15.8 to -13.2)**	**23 267 943 (22 477 274 to 24 100 845)**	**-51.7 (-56.7 to -48.0)**
China	1 061 467 (1 023 168 to 1 101 973)	-52.4 (-57.9 to -48.4)	1 727 057 (1 579 068 to 1 894 941)	-14.4 (-15.7 to -13.1)	22 407 649 (21 645 283 to 23 221 152)	-52.5 (-57.4 to -48.7)
North Korea	31 305 (27 451 to 35 612)	27.1 (7.7 to 51.1)	26 847 (24 417 to 29 618)	10.9 (8.8 to 13.0)	708 781 (620 583 to 799 742)	31.4 (11.5 to 54.8)
Taiwan (Province of China)	7 037 (5 971 to 8 098)	-68.4 (-73.2 to -62.9)	14 035 (12 621 to 15 516)	-39.9 (-41.3 to -38.6)	151 512 (129 205 to 174 985)	-65.7 (-70.9 to -60.2)
**Southeast Asia**	**331 402 (314 980 to 347 885)**	**-21.9 (-27.8 to -15.3)**	**312 080 (284 279 to 342 274)**	**-15.3 (-16.3 to -14.2)**	**8 230 360 (7 841 393 to 8 612 603)**	**-21.1 (-26.8 to -15.4)**
Cambodia	7 887 (7 067 to 8 721)	-28.2 (-39.6 to -10.2)	6 164 (5 613 to 6 785)	-23.2 (-24.6 to -21.8)	197 129 (176 422 to 219 008)	-31.9 (-43.0 to -17.5)
Indonesia	142 688 (133 365 to 152 970)	6.3 (-4.4 to 19.1)	127 135 (115 332 to 139 666)	3.2 (1.9 to 4.5)	3 693 542 (3 476 360 to 3 925 592)	4.6 (-4.6 to 14.4)
Laos	3 124 (2 690 to 3 492)	-31.9 (-41.3 to -18.5)	2 533 (2 289 to 2 782)	-22.9 (-24.3 to -21.6)	90 235 (77 191 to 103 025)	-36.0 (-45.5 to -25.3)
Malaysia	9 599 (8 713 to 10 540)	-46.9 (-53.1 to -40.3)	12 360 (11 172 to 13 595)	-26.8 (-28.2 to -25.3)	228 722 (207 772 to 250 134)	-49.1 (-54.6 to -43.4)
Maldives	54 (43 to 67)	-65.3 (-73.6 to -55.1)	94 (84 to 104)	-36.9 (-38.4 to -35.3)	1 241 (1 003 to 1 530)	-70.0 (-78.4 to -61.0)
Mauritius	549 (476 to 625)	-62.2 (-67.5 to -57.0)	688 (621 to 762)	-45.2 (-46.3 to -43.9)	12 187 (10 559 to 13 947)	-62.8 (-68.1 to -57.3)

Myanmar	26 256 (23 237 to 29 810)	-40.2 (-48.9 to -29.5)	24 087 (21 756 to 26 559)	-28.1 (-29.5 to -26.7)	645 862 (568 991 to 745 880)	-42.5 (-51.5 to -31.8)
Philippines	49 026 (42 645 to 56 041)	22.4 (6.7 to 40.9)	43 301 (39 274 to 47 733)	11.4 (9.1 to 13.5)	1 333 884 (1 155 697 to 1 526 225)	18.1 (2.1 to 36.2)
Sri Lanka	4 807 (3 874 to 5 935)	-29.9 (-45.1 to -11.7)	8 301 (7 389 to 9 244)	-8.1 (-10.0 to -6.1)	106 187 (85 734 to 130 863)	-24.7 (-41.8 to -5.0)
Seychelles	30 (26 to 35)	-39.7 (-49.1 to -28.9)	43 (39 to 48)	-19.4 (-21.1 to -17.7)	733 (627 to 860)	-41.6 (-51.9 to -30.2)
Thailand	24 577 (21 909 to 27 598)	-53.7 (-59.6 to -47.2)	35 185 (31 679 to 38 668)	-33.2 (-34.5 to -32.1)	598 135 (525 330 to 676 529)	-49.6 (-56.3 to -42.3)
Timor-Leste	392 (299 to 509)	-35.3 (-51.5 to -8.4)	378 (336 to 424)	-20.6 (-22.2 to -18.8)	9 373 (6 822 to 12 257)	-40.2 (-56.5 to -18.3)
Vietnam	62 413 (55 228 to 70 138)	-35.2 (-46.0 to -20.8)	51 194 (46 491 to 56 378)	-29.8 (-31.1 to -28.5)	1 312 693 (1 142 609 to 1 511 735)	-37.6 (-49.0 to -23.8)
**Oceania**	**6 039 (5 071 to 7 076)**	**-19.9 (-31.2 to -6.8)**	**5 019 (4 568 to 5 504)**	**-13.7 (-14.9 to -12.5)**	**199 832 (167 249 to 237 618)**	**-18.6 (-31.4 to -4.1)**
American Samoa	19 (16 to 23)	-32.9 (-44.5 to -19.7)	28 (25 to 31)	-17.7 (-19.4 to -16.1)	576 (479 to 684)	-34.7 (-46.6 to -20.6)
Federated States of Micronesia	65 (51 to 84)	-16.4 (-34.5 to 6.5)	48 (43 to 53)	-14.2 (-15.9 to -12.6)	1 777 (1 367 to 2 287)	-16.9 (-37.7 to 8.0)
Fiji	362 (283 to 456)	-23.0 (-42.4 to 4.4)	415 (370 to 463)	-12.1 (-14.0 to -10.2)	10 033 (7 832 to 12 594)	-22.3 (-43.6 to 6.0)
Guam	65 (57 to 75)	-8.7 (-24.3 to 8.9)	100 (90 to 111)	1.4 (-0.5 to 3.2)	1 620 (1 396 to 1 876)	-5.3 (-21.6 to 12.9)
Kiribati	71 (61 to 82)	-14.0 (-26.9 to 1.3)	52 (47 to 57)	-14.2 (-15.8 to -12.4)	2 295 (1 964 to 2 674)	-13.3 (-26.9 to 3.1)
Marshall Islands	29 (24 to 35)	-17.1 (-30.3 to -2.0)	27 (24 to 29)	-11.4 (-13.0 to -9.8)	947 (775 to 1 134)	-15.5 (-30.0 to 0.8)
Northern Mariana Islands	19 (16 to 24)	-35.1 (-48.7 to -16.6)	31 (28 to 35)	-19.2 (-20.8 to -17.7)	681 (540 to 837)	-34.2 (-49.9 to -15.4)
Papua New Guinea	4 705 (3 748 to 5 731)	-19.2 (-33.5 to -1.1)	3 243 (2 941 to 3 557)	-14.8 (-16.2 to -13.3)	160 764 (128 950 to 197 513)	-20.4 (-35.6 to -2.2)

Samoa	88 (71 to 105)	-28.0 (-40.8 to -14.8)	91 (82 to 100)	-16.0 (-17.6 to -14.3)	2 113 (1 708 to 2 526)	-29.9 (-42.6 to -16.9)
Solomon Islands	393 (324 to 482)	-12.2 (-26.2 to 7.3)	257 (233 to 281)	-12.5 (-14.1 to -10.9)	12 118 (9 863 to 15 031)	-11.6 (-27.9 to 10.5)
Tonga	34 (29 to 39)	-21.2 (-35.8 to -2.7)	42 (38 to 46)	-8.8 (-10.3 to -7.1)	796 (671 to 920)	-22.2 (-37.3 to -4.6)
Vanuatu	188 (152 to 234)	-12.1 (-27.8 to 6.8)	138 (125 to 151)	-13.1 (-14.6 to -11.6)	5 774 (4 649 to 7 251)	-11.2 (-29.2 to 10.2)
**North Africa and Middle East**	**101 824 (92 222 to 112 158)**	**-28.3 (-33.5 to -22.0)**	**146 502 (132 330 to 161 357)**	**-13.7 (-14.8 to -12.6)**	**2 808 514 (2 573 765 to 3 098 537)**	**-33.3 (-37.9 to -28.1)**
Afghanistan	13 108 (10 862 to 15 845)	-1.0 (-14.2 to 14.7)	8 422 (7 617 to 9 301)	-11.4 (-12.9 to -9.8)	412 503 (334 089 to 510 326)	-3.4 (-16.9 to 11.5)
Algeria	6 959 (5 729 to 8 312)	-36.7 (-45.3 to -26.6)	11 290 (10 164 to 12 515)	-21.0 (-22.5 to -19.5)	158 399 (131 940 to 191 644)	-45.0 (-53.1 to -35.6)
Bahrain	67 (54 to 82)	-63.4 (-70.7 to -54.1)	220 (193 to 251)	-32.3 (-33.7 to -30.9)	2 053 (1 682 to 2 539)	-65.7 (-72.6 to -57.0)
Egypt	21 210 (17 430 to 25 333)	-30.0 (-40.6 to -16.7)	27 273 (24 484 to 30 257)	-11.2 (-12.9 to -9.6)	552 204 (456 233 to 657 727)	-31.7 (-41.8 to -19.7)
Iran	6 287 (5 285 to 7 466)	-28.5 (-45.0 to -6.0)	15 315 (13 568 to 17 077)	-5.7 (-7.3 to -4.1)	171 103 (143 217 to 203 563)	-30.8 (-47.2 to -9.0)
Iraq	8 435 (6 874 to 10 226)	-17.3 (-36.1 to 2.8)	9 107 (8 188 to 10 000)	-14.9 (-16.6 to -13.2)	258 093 (205 776 to 318 386)	-20.2 (-39.8 to 0.3)
Jordan	565 (439 to 724)	-46.9 (-59.2 to -30.4)	1 364 (1 218 to 1 515)	-17.7 (-19.4 to -16.1)	14 648 (11 478 to 18 784)	-49.4 (-62.2 to -33.3)
Kuwait	165 (123 to 219)	-10.8 (-34.3 to 18.4)	618 (535 to 702)	7.2 (4.8 to 9.5)	6 108 (4 657 to 7 947)	-9.1 (-32.3 to 17.6)
Lebanon	426 (315 to 572)	-71.6 (-79.5 to -60.4)	1 490 (1 314 to 1 674)	-30.4 (-32.0 to -28.8)	9 918 (7 799 to 12 589)	-74.6 (-81.5 to -66.1)
Libya	823 (658 to 1 022)	-25.5 (-38.1 to -10.8)	1 646 (1 478 to 1 826)	-4.6 (-6.5 to -2.6)	24 535 (20 063 to 29 940)	-45.1 (-60.5 to -30.7)
Morocco	6 667 (5 042 to 8 515)	-31.4 (-41.5 to -13.9)	10 752 (9 680 to 11 945)	-13.1 (-14.7 to -11.5)	157 201 (121 879 to 201 933)	-39.0 (-47.4 to -29.1)

Palestine	801<br>(734 to 876)	-52.6<br>(-59.6 to -43.9)	901<br>(816 to 995)	-43.1<br>(-44.4 to -41.9)	19 017<br>(17 332 to 21 250)	-52.5<br>(-59.9 to -43.4)
Oman	644<br>(570 to 719)	-49.6<br>(-60.3 to -36.7)	1 034<br>(925 to 1 142)	-26.6<br>(-28.1 to -25.2)	19 529<br>(17 245 to 21 955)	-63.2<br>(-72.1 to -53.4)
Qatar	87<br>(63 to 117)	-67.3<br>(-76.1 to -55.0)	336<br>(291 to 388)	-33.8<br>(-35.4 to -32.3)	3 769<br>(2 844 to 4 929)	-66.5<br>(-75.5 to -54.6)
Saudi Arabia	3 097<br>(2 775 to 3 452)	-37.0<br>(-49.0 to -21.3)	6 335<br>(5 617 to 7 062)	-18.6<br>(-19.6 to -17.7)	76 034<br>(68 345 to 84 885)	-42.0<br>(-52.5 to -30.0)
Sudan	7 840<br>(6 372 to 9 256)	-33.9<br>(-41.9 to -23.8)	8 972<br>(8 089 to 9 895)	-19.7<br>(-21.0 to -18.1)	260 053<br>(210 699 to 318 628)	-39.8<br>(-47.4 to -31.7)
Syria	2 858<br>(2 558 to 3 191)	-48.3<br>(-55.7 to -39.2)	4 210<br>(3 801 to 4 633)	-29.1<br>(-30.5 to -27.7)	90 507<br>(81 007 to 101 569)	-54.8<br>(-61.8 to -47.5)
Tunisia	2 379<br>(1 831 to 3 032)	-35.8<br>(-48.7 to -20.7)	3 946<br>(3 537 to 4 411)	-16.1<br>(-17.9 to -14.3)	48 882<br>(38 232 to 62 399)	-42.3<br>(-53.7 to -29.3)
Turkey	11 944<br>(10 010 to 14 153)	-23.9<br>(-38.3 to -5.5)	25 194<br>(22 616 to 27 944)	-8.6<br>(-10.5 to -6.7)	275 805<br>(234 292 to 326 061)	-37.1<br>(-49.9 to -23.1)
United Arab Emirates	1 342<br>(1 020 to 1 746)	-30.3<br>(-49.3 to -3.7)	2 123<br>(1 860 to 2 406)	-22.8<br>(-24.2 to -21.3)	52 135<br>(39 726 to 68 346)	-30.4<br>(-50.6 to -4.3)
Yemen	6 121<br>(5 037 to 7 217)	-29.9<br>(-41.3 to -15.3)	5 817<br>(5 222 to 6 412)	-22.1<br>(-23.6 to -20.6)	195 929<br>(159 741 to 234 952)	-37.4<br>(-47.4 to -25.5)
**South Asia**	**461 021<br>(422 318 to 503 462)**	**-29.9<br>(-37.7 to -23.0)**	**468 903<br>(422 534 to 519 230)**	**-16.0<br>(-17.0 to -15.0)**	**12 125 315<br>(11 216 203 to 13 108 598)**	**-30.3<br>(-37.0 to -24.5)**
Bangladesh	59 799<br>(50 982 to 68 907)	-33.0<br>(-44.1 to -19.3)	52 581<br>(47 755 to 57 499)	-21.6<br>(-23.2 to -19.9)	1 616 636<br>(1 396 376 to 1 856 714)	-25.9<br>(-38.2 to -11.2)
Bhutan	152<br>(125 to 185)	-53.9<br>(-63.0 to -43.8)	180<br>(162 to 200)	-31.8<br>(-33.2 to -30.3)	3 628<br>(2 928 to 4 478)	-55.9<br>(-65.1 to -45.8)
India	334 688<br>(302 445 to 372 243)	-31.5<br>(-39.1 to -24.0)	356 177<br>(320 207 to 395 467)	-14.7<br>(-15.7 to -13.7)	8 824 016<br>(8 072 477 to 9 677 563)	-32.6<br>(-39.3 to -25.9)
Nepal	7 237<br>(6 183 to 8 374)	-38.7<br>(-49.8 to -25.7)	7 853<br>(7 047 to 8 719)	-19.5<br>(-20.8 to -18.0)	178 310<br>(152 636 to 205 223)	-41.4<br>(-51.9 to -30.1)

Pakistan	59 146 (49 838 to 69 411)	-19.2 (-34.2 to -0.4)	52 112 (46 736 to 57 665)	-20.5 (-22.2 to -19.0)	1 502 725 (1 266 268 to 1 775 050)	-20.2 (-35.2 to -1.9)
**Southern sub-Saharan Africa**	**19 731 (18 454 to 20 976)**	**-7.2 (-15.1 to 2.2)**	**20 016 (18 237 to 21 870)**	**-5.1 (-6.2 to -4.0)**	**516 264 (478 602 to 553 123)**	**-10.2 (-17.9 to -1.1)**
Botswana	539 (264 to 777)	-27.3 (-62.4 to 6.6)	483 (428 to 540)	-20.8 (-22.5 to -19.1)	13 270 (6 335 to 19 657)	-27.1 (-64.1 to 9.9)
Lesotho	1 115 (817 to 1 456)	12.7 (-17.6 to 53.5)	596 (537 to 661)	-8.2 (-10.2 to -6.3)	25 293 (18 482 to 33 387)	18.4 (-16.2 to 61.8)
Namibia	558 (368 to 739)	-44.1 (-62.3 to -26.8)	507 (453 to 566)	-36.3 (-37.9 to -34.7)	12 684 (8 323 to 16 917)	-48.8 (-65.9 to -32.5)
South Africa	13 523 (12 673 to 14 451)	-8.9 (-16.5 to 0.1)	15 101 (13 733 to 16 533)	-7.1 (-8.3 to -6.0)	317 788 (296 655 to 340 840)	-19.3 (-26.4 to -11.7)
Swaziland	342 (221 to 491)	-25.9 (-48.3 to 1.4)	260 (231 to 291)	-21.9 (-23.6 to -20.2)	8 160 (5 279 to 11 776)	-25.8 (-49.9 to 4.3)
Zimbabwe	3 654 (3 000 to 4 412)	11.6 (-17.0 to 77.3)	3 069 (2 793 to 3 363)	18.7 (16.3 to 21.0)	139 069 (115 749 to 166 299)	39.8 (2.2 to 122.6)
**Western sub-Saharan Africa**	**64 450 (58 752 to 70 023)**	**-16.8 (-25.5 to -7.4)**	**67 770 (61 096 to 74 667)**	**-11.2 (-12.4 to -10.0)**	**1 978 272 (1 796 108 to 2 157 207)**	**-20.0 (-28.4 to -11.6)**
Benin	2 528 (2 170 to 2 915)	0.0 (-13.4 to 16.3)	2 244 (2 028 to 2 461)	-4.3 (-6.1 to -2.5)	75 702 (64 913 to 87 263)	-5.4 (-18.2 to 9.2)
Burkina Faso	2 850 (2 411 to 3 329)	11.6 (-6.4 to 37.6)	2 870 (2 554 to 3 177)	7.1 (5.1 to 9.1)	84 779 (71 725 to 99 474)	4.7 (-12.4 to 27.3)
Cameroon	5 528 (4 130 to 7 165)	-1.8 (-25.2 to 26.3)	4 592 (4 135 to 5 076)	-7.9 (-9.9 to -6.1)	163 763 (120 979 to 211 417)	1.2 (-24.6 to 29.0)
Cape Verde	121 (102 to 140)	-36.9 (-47.3 to -25.5)	142 (129 to 155)	-23.4 (-24.9 to -21.9)	2 842 (2 357 to 3 334)	-41.1 (-51.4 to -29.2)
Chad	2 681 (2 223 to 3 190)	-7.2 (-21.8 to 10.1)	2 473 (2 228 to 2 725)	-4.5 (-6.4 to -2.6)	87 638 (72 620 to 104 963)	-8.9 (-23.3 to 7.8)
Cote d'Ivoire	7 050 (5 940 to 8 239)	2.4 (-12.0 to 19.1)	5 382 (4 852 to 5 921)	-8.6 (-10.3 to -6.9)	220 676 (184 566 to 261 984)	0.7 (-14.0 to 18.5)

The Gambia	298<br>(251 to 348)	-7.0<br>(-23.2 to 10.6)	344<br>(309 to 380)	-3.2<br>(-5.0 to -1.6)	9 057<br>(7 687 to 10 498)	-11.0<br>(-26.3 to 4.4)
Ghana	7 295<br>(6 217 to 8 426)	-21.4<br>(-36.1 to -3.0)	6 365<br>(5 740 to 6 996)	-16.5<br>(-18.0 to -14.9)	204 005<br>(175 536 to 234 599)	-21.5<br>(-36.3 to -3.3)
Guinea	3 489<br>(2 942 to 4 102)	8.3<br>(-10.1 to 28.4)	2 750<br>(2 477 to 3 048)	-1.7<br>(-3.5 to 0.1)	104 956<br>(89 128 to 123 276)	2.8<br>(-14.8 to 21.7)
Guinea-Bissau	730<br>(608 to 865)	-6.7<br>(-22.1 to 11.8)	494<br>(446 to 545)	-10.3<br>(-11.8 to -8.8)	22 368<br>(18 656 to 26 584)	-10.3<br>(-25.6 to 7.3)
Liberia	1 025<br>(893 to 1 156)	2.4<br>(-11.9 to 20.2)	947<br>(856 to 1 045)	-2.4<br>(-4.1 to -0.8)	29 495<br>(25 831 to 33 168)	-5.3<br>(-18.9 to 9.8)
Mali	3 256<br>(2 631 to 3 989)	-29.6<br>(-43.5 to -11.3)	3 016<br>(2 721 to 3 346)	-19.7<br>(-21.3 to -18.1)	108 141<br>(87 947 to 131 046)	-33.5<br>(-47.7 to -17.1)
Mauritania	576<br>(419 to 760)	-37.1<br>(-50.6 to -21.2)	740<br>(662 to 828)	-19.7<br>(-21.2 to -18.1)	16 656<br>(12 275 to 21 516)	-41.5<br>(-54.9 to -26.4)
Niger	4 088<br>(3 045 to 5 258)	-6.8<br>(-25.6 to 17.3)	3 605<br>(3 212 to 4 016)	-7.0<br>(-8.7 to -5.3)	129 658<br>(96 513 to 164 221)	-14.0<br>(-31.6 to 8.9)
Nigeria	16 415<br>(12 903 to 19 812)	-37.8<br>(-51.0 to -22.0)	25 793<br>(22 943 to 28 713)	-17.4<br>(-19.1 to -15.7)	518 362<br>(426 418 to 615 860)	-41.2<br>(-54.2 to -26.7)
Sao Tome and Principe	45<br>(37 to 53)	-6.5<br>(-24.7 to 13.2)	43<br>(39 to 47)	-11.4<br>(-13.3 to -9.6)	1 204<br>(974 to 1 464)	-17.7<br>(-34.3 to 0.3)
Senegal	3 131<br>(2 685 to 3 652)	4.9<br>(-8.9 to 21.8)	3 089<br>(2 803 to 3 399)	-0.1<br>(-2.0 to 1.9)	91 111<br>(77 944 to 105 253)	1.2<br>(-11.7 to 16.9)
Sierra Leone	1 621<br>(1 388 to 1 852)	11.6<br>(-5.1 to 31.6)	1 404<br>(1 266 to 1 554)	4.2<br>(2.2 to 5.9)	54 811<br>(46 811 to 62 990)	3.9<br>(-11.8 to 21.2)
Togo	1 723<br>(1 426 to 2 076)	-4.2<br>(-19.6 to 12.9)	1 472<br>(1 322 to 1 616)	-6.1<br>(-8.1 to -4.3)	53 049<br>(43 967 to 63 671)	-5.2<br>(-19.7 to 11.5)
**Eastern sub-Saharan Africa**	**87 523<br>(78 640 to 99 403)**	**-37.6<br>(-43.7 to -28.3)**	**67 823<br>(61 077 to 74 894)**	**-27.9<br>(-28.8 to -26.9)**	**2 263 201<br>(2 041 787 to 2 541 233)**	**-40.5<br>(-46.6 to -32.9)**
Burundi	3 005<br>(2 407 to 3 672)	-51.8<br>(-61.7 to -39.6)	2 024<br>(1 817 to 2 244)	-39.9<br>(-41.2 to -38.7)	81 809<br>(65 906 to 98 843)	-55.6<br>(-65.5 to -44.3)

Comoros	139 (112 to 181)	-50.5 (-60.0 to -37.9)	131 (117 to 145)	-36.8 (-38.1 to -35.4)	3 787 (3 024 to 4 895)	-54.8 (-63.9 to -43.5)
Djibouti	201 (141 to 280)	-35.6 (-51.8 to -17.4)	191 (171 to 213)	-21.9 (-23.5 to -20.5)	5 246 (3 693 to 7 446)	-38.2 (-54.7 to -22.0)
Eritrea	1 268 (985 to 1 616)	-48.3 (-56.6 to -38.2)	841 (749 to 935)	-36.7 (-38.1 to -35.5)	34 298 (26 883 to 43 739)	-52.3 (-60.2 to -42.4)
Ethiopia	26 333 (21 815 to 31 005)	-46.2 (-55.8 to -30.8)	19 547 (17 577 to 21 621)	-33.5 (-34.8 to -32.2)	643 859 (533 164 to 756 663)	-49.6 (-59.4 to -36.1)
Kenya	6 955 (5 604 to 8 765)	-22.0 (-32.8 to -6.0)	6 979 (6 181 to 7 793)	-12.9 (-13.8 to -12.0)	175 029 (141 326 to 219 720)	-22.5 (-33.1 to -9.4)
Madagascar	11 632 (8 975 to 14 710)	-14.9 (-33.1 to 3.7)	6 325 (5 733 to 6 976)	-26.6 (-28.1 to -25.0)	338 801 (264 142 to 429 870)	-18.1 (-35.8 to 0.3)
Malawi	2 993 (2 305 to 3 767)	-26.9 (-44.3 to -3.7)	2 758 (2 453 to 3 090)	-19.3 (-20.9 to -17.6)	73 727 (56 985 to 92 126)	-30.2 (-48.2 to -8.7)
Mozambique	7 429 (6 018 to 9 192)	-37.9 (-49.8 to -23.1)	5 670 (5 099 to 6 299)	-29.3 (-31.0 to -27.8)	184 079 (150 332 to 225 352)	-39.9 (-51.8 to -26.0)
Rwanda	1 945 (1 486 to 2 422)	-60.8 (-70.4 to -49.0)	1 675 (1 480 to 1 866)	-45.9 (-47.1 to -44.7)	48 977 (37 521 to 61 548)	-64.8 (-74.6 to -53.5)
Somalia	3 415 (2 728 to 4 287)	-30.4 (-42.1 to -15.6)	2 119 (1 898 to 2 336)	-26.3 (-27.7 to -24.9)	92 985 (74 439 to 116 909)	-33.6 (-45.3 to -19.1)
South Sudan	3 091 (2 267 to 4 008)	-20.4 (-38.8 to 2.7)	2 481 (2 227 to 2 749)	-17.7 (-19.2 to -16.2)	80 313 (59 062 to 104 318)	-23.7 (-43.0 to -0.7)
Tanzania	9 006 (7 257 to 10 875)	-33.6 (-47.2 to -17.7)	8 810 (7 844 to 9 803)	-20.2 (-21.9 to -18.6)	233 955 (189 636 to 281 682)	-35.3 (-48.6 to -20.6)
Uganda	6 628 (5 410 to 7 909)	-39.2 (-50.3 to -25.1)	5 510 (4 899 to 6 134)	-28.1 (-29.4 to -26.9)	174 112 (143 232 to 207 851)	-42.8 (-54.1 to -28.9)
Zambia	3 482 (2 649 to 4 384)	1.5 (-28.2 to 44.0)	2 709 (2 419 to 3 005)	-11.4 (-13.2 to -9.5)	92 208 (70 509 to 116 912)	5.7 (-27.7 to 47.9)
**Central sub-Saharan Africa**	**29 664 (25 202 to 33 547)**	**-16.2 (-25.7 to -6.0)**	**22 704 (20 452 to 25 006)**	**-17.6 (-18.9 to -16.2)**	**770 847 (671 847 to 869 560)**	**-20.8 (-29.6 to -11.1)**

Angola	4 960 (3 674 to 6 639)	-31.4 (-48.3 to -8.0)	4 319 (3 849 to 4 797)	-24.3 (-26.0 to -22.6)	134 233 (99 018 to 180 838)	-35.5 (-53.2 to -13.4)
Central African Republic	2 527 (1 943 to 3 136)	-8.5 (-27.2 to 10.9)	1 383 (1 249 to 1 534)	-18.9 (-20.7 to -17.2)	67 017 (51 452 to 83 752)	-9.1 (-29.1 to 11.1)
Congo (Brazzaville)	1 263 (984 to 1 560)	-43.3 (-56.3 to -28.5)	1 016 (913 to 1 120)	-33.2 (-34.7 to -31.9)	31 422 (24 238 to 39 303)	-46.9 (-60.4 to -32.4)
DR Congo	20 320 (16 617 to 23 820)	-6.0 (-19.3 to 9.7)	15 393 (13 859 to 16 980)	-12.3 (-14.1 to -10.5)	524 844 (443 077 to 609 391)	-10.9 (-23.1 to 2.4)
Equatorial Guinea	117 (67 to 179)	-72.6 (-83.4 to -58.0)	157 (139 to 178)	-46.6 (-47.9 to -45.3)	2 947 (1 686 to 4 592)	-76.6 (-86.4 to -63.8)
Gabon	477 (383 to 583)	-38.7 (-51.7 to -21.5)	436 (392 to 483)	-30.5 (-31.8 to -29.2)	10 384 (8 178 to 12 875)	-42.6 (-56.0 to -26.4)
